# Migratory movements of fin whales from the Gulf of St. Lawrence challenge our understanding of the Northwest Atlantic stock structure

**DOI:** 10.1038/s41598-024-62173-1

**Published:** 2024-05-20

**Authors:** Christian Ramp, Veronique Lesage, Angélique Ollier, Marie Auger-Méthé, Richard Sears

**Affiliations:** 1 Fisheries and Oceans Canada, Maurice Lamontagne Institute, 850 Route de la mer,, Mont Joli, QC G5H 3Z4 Canada; 2 Institute for the Oceans and Fisheries, AERL, 2202 Main Mall, University of British Columbia, Vancouver, V6T 1Z4 Canada; 3 Department of Statistics, ESB, 2207 Main Mall, University of British Columbia, Vancouver, V6T 1Z4 Canada; 4 Mingan Island Cetacean Study, 285 rue Green, St Lambert, QC J4P 1T3 Canada

**Keywords:** Ecology, Ecology, Ocean sciences

## Abstract

Fin whales, *Balenoptera physalus*, are capital breeders, having the potential to separate breeding and feeding both spatially and temporally. Fin whales occur throughout the Northwest Atlantic, but stock structure and seasonal movements remain unclear. By deploying satellite transmitters on 28 individuals, we examine movement patterns within and beyond the Gulf of St. Lawrence (GSL), Canada, and challenge the current understanding of stock structure. Eight individuals left the GSL in autumn, with five tags persisting into January. Migration patterns of these whales showed considerable variation in timing and trajectory, with movements extending south to 24°N, and thus beyond the assumed distribution limit of the species in the Northwest Atlantic. A rapid return to the Scotian Shelf or Gulf of Maine was observed from several whales after incursions in southern waters, suggesting that fin whales in the Northwest Atlantic may not have a common winter destination that fits the definition of a breeding ground. Area-restricted search (ARS) behavior dominated fin whale activities during summer (92%) and fall (72%), with persistence into the winter (56%); ARS occurred at multiple locations in the GSL, Scotian Shelf and Shelf edge, and near seamounts of the North Atlantic, having characteristics consistent with foraging areas.

## Introduction

The traditional view of baleen whale migration predicts that animals benefit from high latitude productivity during summer to build up energy reserves and then migrate to warmer, lower latitude waters for breeding and calving^1,2^. This spatial separation of feeding from breeding areas is typical of capital breeders. It is enabled in baleen whales by their enormous body size, which allows them to store large energy reserves^3–5^, and results in low mass-specific metabolic rate and low cost of transport^5–7^. These large-scale north-south seasonal movements apply to most humpback (*Megaptera novaeangliae*), gray (*Eschrichtius robustus*) and right whale (*Eubalena spp)* populations, which have clearly defined coastal breeding and calving grounds^8–10^. However, recent research indicates that there exists a continuum of migration strategies among all baleen whale species and populations that involve different degrees of seasonal movement and foraging^11–14^. These observations raised questions about the reasons for migration in baleen whales, e.g.^15,16^.

Several factors that are not mutually exclusive can influence the timing and extent of seasonal movements of baleen whales and other marine species: whales could migrate to lower latitudes during the breeding season to find a mate, to reduce heat loss for calves or to reduce killer whale (*Orcinus Orca*) predation; they could also be forced out of foraging areas by a reduced productivity or food access due to increased sea ice extent in search for more accessible feeding grounds^6,15–18^. If migration is related exclusively to mating and calving, we would expect some interindividual variability according to body condition and reproductive status. If migration is related to sea ice and food access, climate variability would be expected to induce similarly variable migratory patterns, with lesser movements during poor-year ice, particularly in non-reproducing whales. Whether cultural inheritance of migration routes and migratory patterns interplays with these other biological or environmental factors is unknown.

Fin whales (*Balaenoptera physalus*) are the second largest baleen whale species after the blue whale (*B. musculus*) and have an almost cosmopolitan distribution outside the tropics^19,20^, although two fin whale subspecies exist in the northern hemisphere (*Balaenoptera physalus physalus*) and southern hemisphere (*Balaenoptera physlau quoyi*)^21^. Most studies report a hiatus in their distribution between 20-30 degrees North and 20 degrees South^19–22^. While acoustic studies detected fin whales south of 20°N in the Atlantic^23,24^, the long distance over which 20 Hz pulse vocalization from fin whales can propagate^23^ makes their presence at these low latitudes unconfirmed. Given their body shape and low cost of locomotion^6,7^, fin whales seem predestined to undergo large-scale migrations. However, no distinct breeding or nursing ground has been identified in this species, suggesting a migratory behavior atypical of a true capital breeder.

Indeed, recent studies indicate that seasonal migration may take many forms in this species^11,13,25^. For instance, while fin whales in the central and eastern North Atlantic undergo large seasonal migrations^12,25^, fin whales in the Mediterranean Sea and the Gulf of California exhibit only limited seasonal movements^13,26–29^. The lack of migration out of these semi-enclosed seas seems to have persisted long enough to make the latter two populations genetically distinct^30–32^.

A year-round presence of whales is observed throughout the species range in the North Atlantic and North Pacific. Although densities shift northward in the spring and southward in the fall/winter^11,23,33–35^, there is a continuous presence of individuals at higher latitudes during winter in the North Atlantic^25,36–39^. Whether the described seasonal shifts in distribution represent movements of individual whales over the entire species’ range or cascading latitudinal movements of distinct populations, as suggested in early studies^1,22,40^, is unknown. Indeed, the stock structure for fin whales in the North Atlantic (and the North Pacific) is uncertain, with the most likely existence of multiple populations^30,31,41–44^. In the Northwest Atlantic, the International Whaling Commission (IWC) recognizes three populations: Nova Scotia-New England (Gulf of Maine), Newfoundland-Labrador, and western Greenland^45^.

Fin whales occur in the Estuary and Gulf of St. Lawrence (Canada) and are thought to use this area mainly for foraging purposes^38,46–48^. Early studies regarded fin whales in the Gulf of St. Lawrence (GSL) as a separate group^22,48^, while song types suggest a connection between these whales and those from the eastern Scotian Shelf and distinct from the whales from New England (Gulf of Maine) and southern Newfoundland^49^. However, using temporal changing song types to distinguish stocks reliably is still debated.

This study examined the seasonal movements and habitat use of fin whales from the GSL using satellite telemetry data in a state-space model framework to identify potential breeding areas, migration routes and important foraging zones. We will provide a perspective on the species’ stock structure in the Northwest Atlantic and increase our understanding of the role of migration in this species in a changing environment.

## Results

### Summary of deployments

We deployed 28 satellite transmitters, eight in the Gulf during the summer and 20 in the Estuary during the fall. Tags transmitted locations for 7 to 115 days (average: 34 d; median: 24 d; Table A Supplement 2, online) and provided a daily average of 1–21 positions (GPS and ARGOS combined) (mean = 9.3, median = 8.3, Table A Supplement 2, online). Most ARGOS positions were of the poorest quality class (Class B; Table A Supplement 2, online). The performance of the GPS was generally low, with, on average, only 10% of the collected positions transmitted via the ARGOS system.

**Figure 1:**
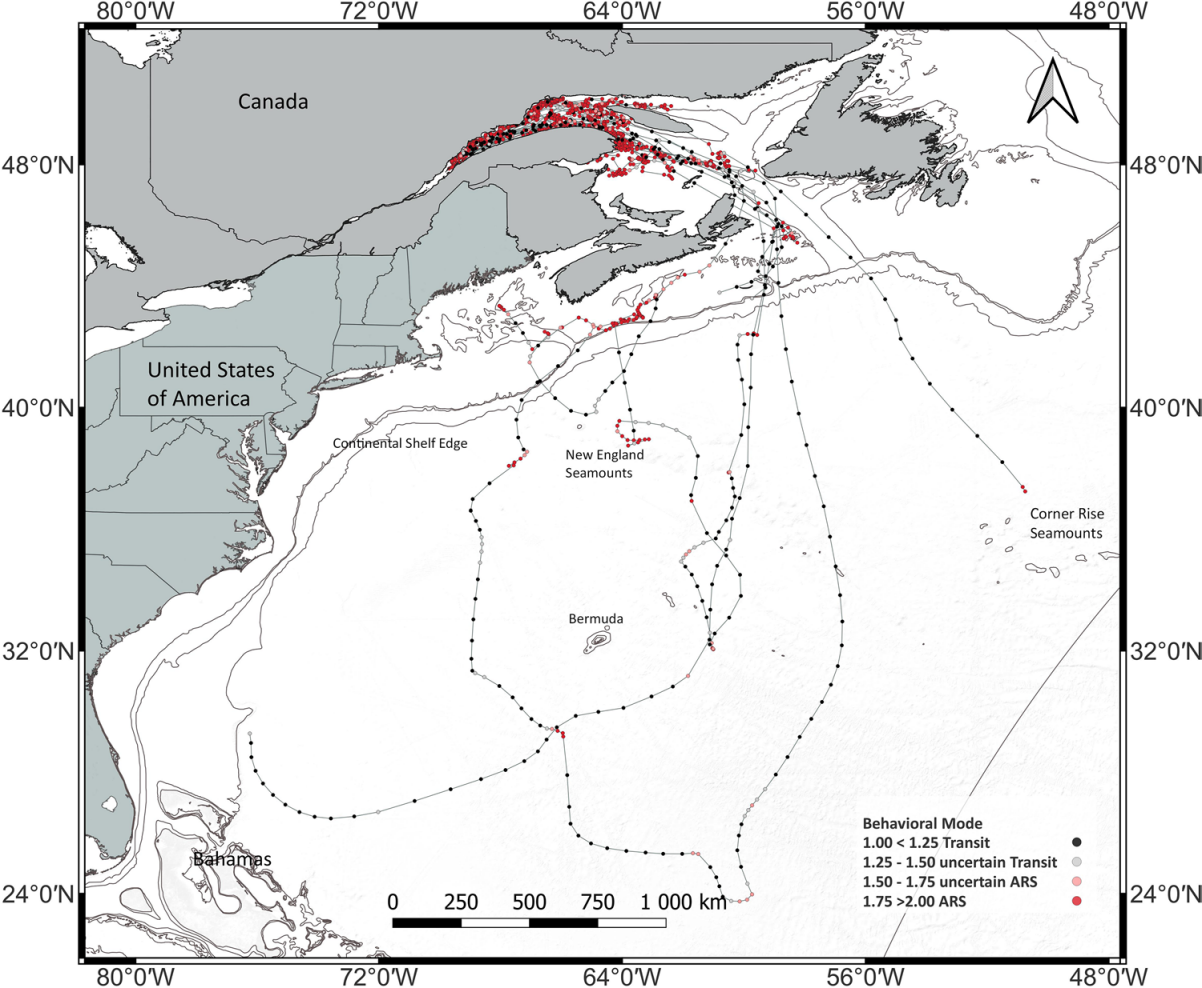
Behavioral states (transit or area-restricted search, ARS) and associated locations as predicted by the hierarchical switching state-space model using all 28 tags/data.

A Bayesian hierarchical switching state-space model (hSSSM) distinguished between two discrete states, fast and straight transiting versus slow tortious area-restricted-search (ARS) based on estimated subsequent locations of fin whales using the original 7773 satellite-derived positions. The model predicted 1656 states/locations in a 12 h step for 28 animals, most of which (71%) were in ARS mode (Table 1, Fig. 1). Animals in transit had a much higher autocorrelation (γ) in speed and direction than animals engaged in ARS (Fig. III Supplement 2, online). The average speed within ARS mode was 0.52 km/h and did not vary with seasons (Table 1). When in transit, fin whales moved up to 10 times faster than when in ARS, with an average speed of 5.7 km/h, and this was true regardless of season (Table 1).

**Table 1:** Number of predicted locations in transit or area-restricted search (ARS) behavior, with average speed (and standard deviation, SD) based on the hierarchical Switching State Space Model (hSSSM) using the entire data set.

	All data	Summer	Fall	Winter
State				
ARS	1176	349	505	322
Uncertain ARS	127	21	56	50
Uncertain transit	79	6	34	39
Transit	274	5	106	163
Percent time in ARS	71%	91.6%	72.0%	56.1%
Speed in km/h				
ARS mean (SD)	0.5 (0.7)	0.5 (0.6)	0.5 (0.7)	0.5 (0.6)
Transit mean (SD)	5.7 (2.5)	4.9 (1.6)	5.1 (1.9)	6.0 (2.8)
Max speed	12.5	10.7	11.4	12.5

We split the data into three parts (summer, fall, and winter) to reduce potential biases of move persistency from long-distance migrations. The analysis led to similar results as when analyzing the entire data set; predicted areas of ARS behavior overlapped almost entirely with those identified based on the whole data set (Figs. IV–VI, Supplement 2, online).

### Movements and migration

Covered distance varied among individual whales from 36 km to 6,993 km (mean 1,300 km, median 681 km, Table A, Supplement 2, online) and was highly dependent on the deployment date and duration and on the date of initiation of the migration (Table A, Supplement 2, online). Of the 20 individuals tagged in the St. Lawrence Estuary in the fall, six stopped providing positions while still in the Estuary, with the latest transmission received from this area on December 13th. The other 14 whales moved east towards the Gulf of St. Lawrence between October 1st and December 19th. Six of these 14 whales stopped transmitting while still in the Gulf, with the latest transmission from this area being received on January 13th (Table 2). The other eight whales eventually exited the GSL and thus fulfilled our definition of migration. All eight individuals exited the Gulf via Cabot Strait, crossing that line between October 30th and January 11th. No fin whale used the northeastern GSL or left the Gulf through the Strait of Belle Isle (Fig. 5). Overall, movements in the fall were unidirectional toward the East, then eventually south. However, two whales tagged in the Estuary during the fall of 2020 left the Estuary, where they were in ARS mode, for a 3–4 day and 300 km journey to the western GSL before returning to the Estuary. One of them conducted that round-trip twice.

**Table 2:** Migration dates for 14 animals leaving the St. Lawrence Estuary in the fall.

Year and Tag	Deploy. date		Leaving estuary	Leaving GSL	Last Trans	Location last Trans	Furthest S point
2016_100386	18-Nov		20-Nov	NA	13-Jan	GSL	
2016_100388	20-Oct		28-Oct	NA	09-Nov	GSL	
2014_106740	04-Nov		07-Nov	27-Dec	05-Jan	Off NS	45 deg N
2015_141348	18-Nov		19-Dec	NA	04-Jan	GSL	
2020_173514	28-Nov		09-Dec	15-Dec	29-Jan	GoM	32 deg N
2020_173515	05-Oct		10-Dec	11-Jan	29-Jan	S. of NS	39 deg N
2020_173518	05-Oct		10-Oct	11-Nov	14-Jan	Off NS	23 deg N
2021_197194	21-Oct		27-Oct	01-Nov	04-Nov	Off NS	43 deg N
2021_197195	28-Nov		11-Dec	15-Dec	05-Jan	Off Florida	26 deg N
2021_197196	21-Oct		26-Oct	NA	06-Nov	GSL	
2021_197202	17-Nov		21-Nov	04-Dec	12-Dec	CRSM	37 deg N
2021_158389	04-Oct		27-Oct	30-Oct	04-Nov	off NS	43 deg N
2021_197177	04-Oct		12-Oct	NA	17-Oct	GSL	
2021_197182	29-Sep	01-Oct		NA	25-Oct	GSL	

*GSL* Gulf of St. Lawrence, *Trans.* Transmission, *NS* Nova Scotia, *GoM* Gulf of Maine, *CRSM* Corner Rise Seamounts, *Furthest S point* Furthest Southern point of migration, *NA* not applicable.

**Figure 2:**
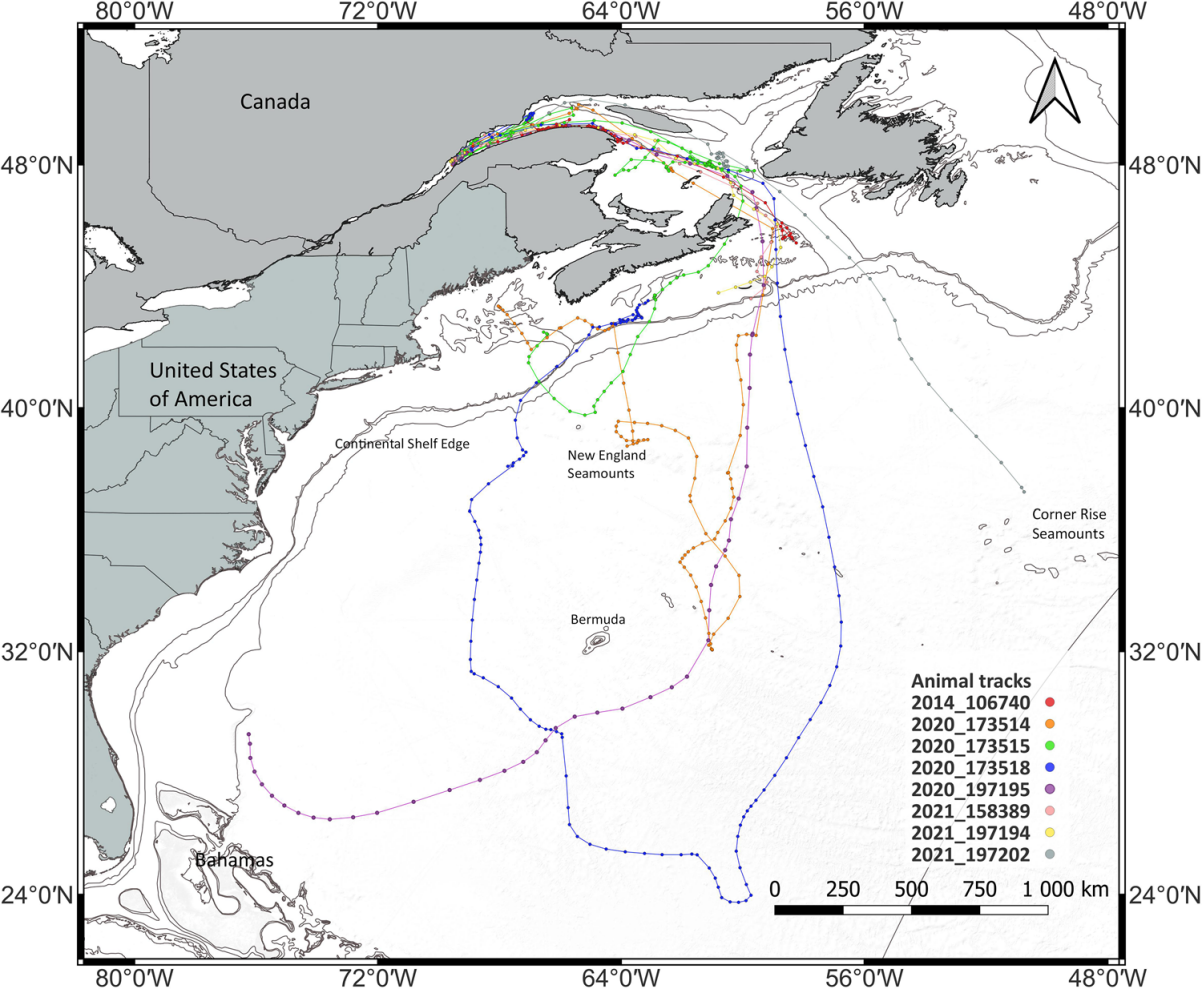
Tracks of the eight fin whales leaving the Gulf of St. Lawrence.

Three of the eight migrating whales stopped transmitting shortly after passing Cabot Strait while still on the eastern Scotian Shelf (Figs. 1 and 2, Table 2). The five remaining whales showed variable migratory patterns, but all went to areas with sea surface temperatures of over 22 degrees Celsius (Fig. VII, Supplement 2, online). One whale moved west on the Scotian Shelf, offshore the Canadian continental shelf, and then back towards the Gulf of Maine. Three other whales headed almost straight south to the East of Bermuda, which they reached in 17–20 days. One of them doubled down on its track, showing some ARS behavior near the New England Seamounts and again along the Scotian Shelf edge before going to the Gulf of Maine. Another circumvented Bermuda and started moving North at the level of the northern Bahamas. This tag stopped transmitting when the whale reached the US continental Shelf, approximately 450 km off Florida. The third whale moved south to 23 degrees latitude North and turned around without a break, heading north again, west of Bermuda. By the end of January, this whale was back on the Scotian Shelf edge off Nova Scotia, exhibiting some ARS behavior. The eighth whale left the GSL and Canadian continental shelf on an approximately south-south-east trajectory and stopped transmitting some 1,300 km away from Cabot Strait in the vicinity of the Corner Rise Seamounts (Fig. 2), with a short episode of ARS behavior before the tag stopped transmitting. The four animals moving the farthest to the south travelled fast and, at times, maintained speeds of over 10 km/h over multiple days.

### Area restricted search (ARS)

There was a decreasing trend in ARS behavior from 92% of the states/locations during summer to 72% during fall and 56% in winter. Conversely, only 1% of the behavioral states during summer were transit versus 15% and 28% in fall and winter, respectively (Table 1). The hSSSM predicted ARS locations in several areas, generally clustered along topographical features.

**Figure 3:**
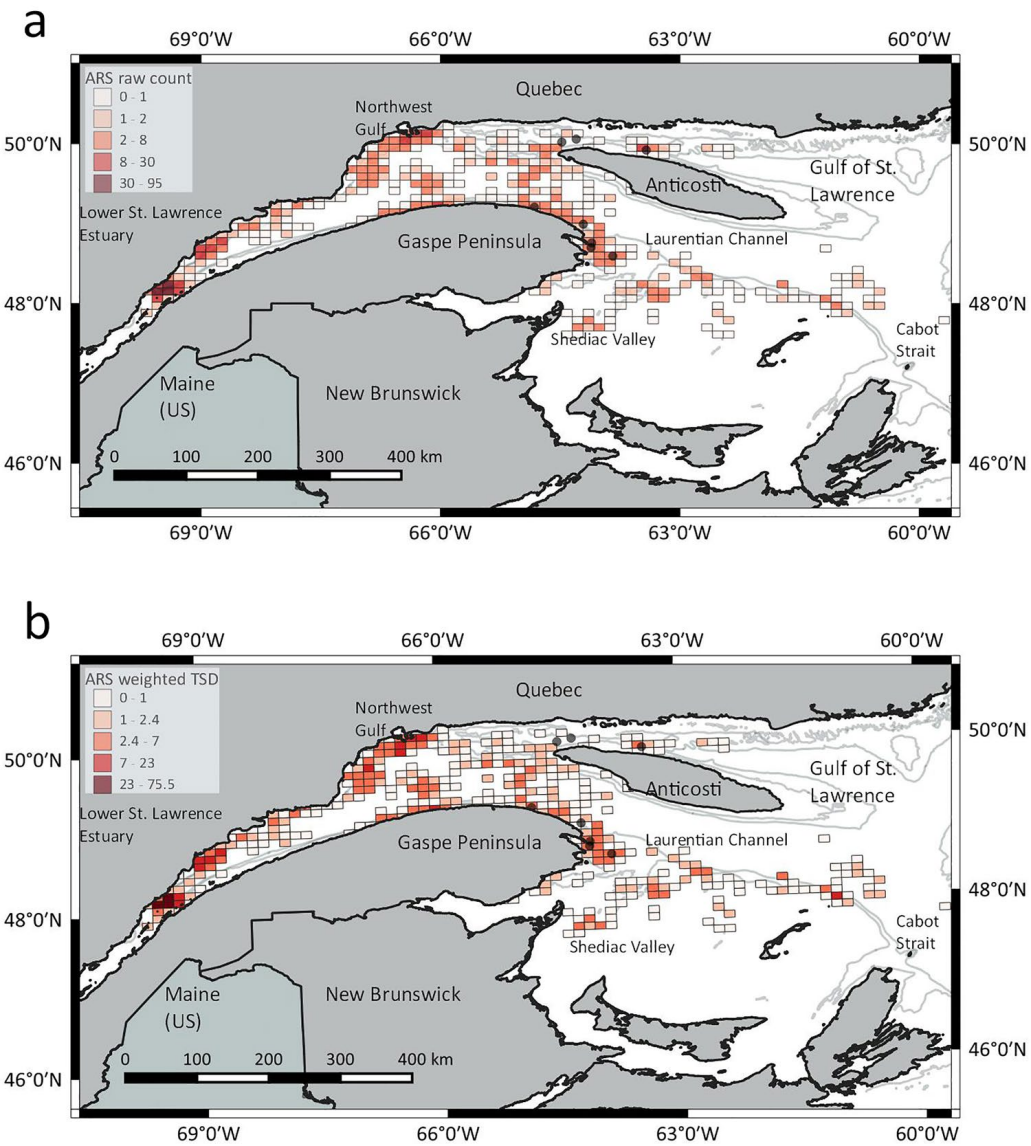
Total area-restricted search (ARS) behavior predicted in a 10*10 km grid over the Gulf of St. Lawrence, using all 28 tags and a 36-hour gap. Representation is based on (a) raw counts and (b) the sum of ARS states weighted for the time since deployment (TSD). The gray dots represent the 8 summer deployment locations. The 20 fall deployments occurred in a very small area of the Lower Estuary and would mask the grid cells. The small scale Fig. 4 of the Estuary shows them.

**Figure 4:**
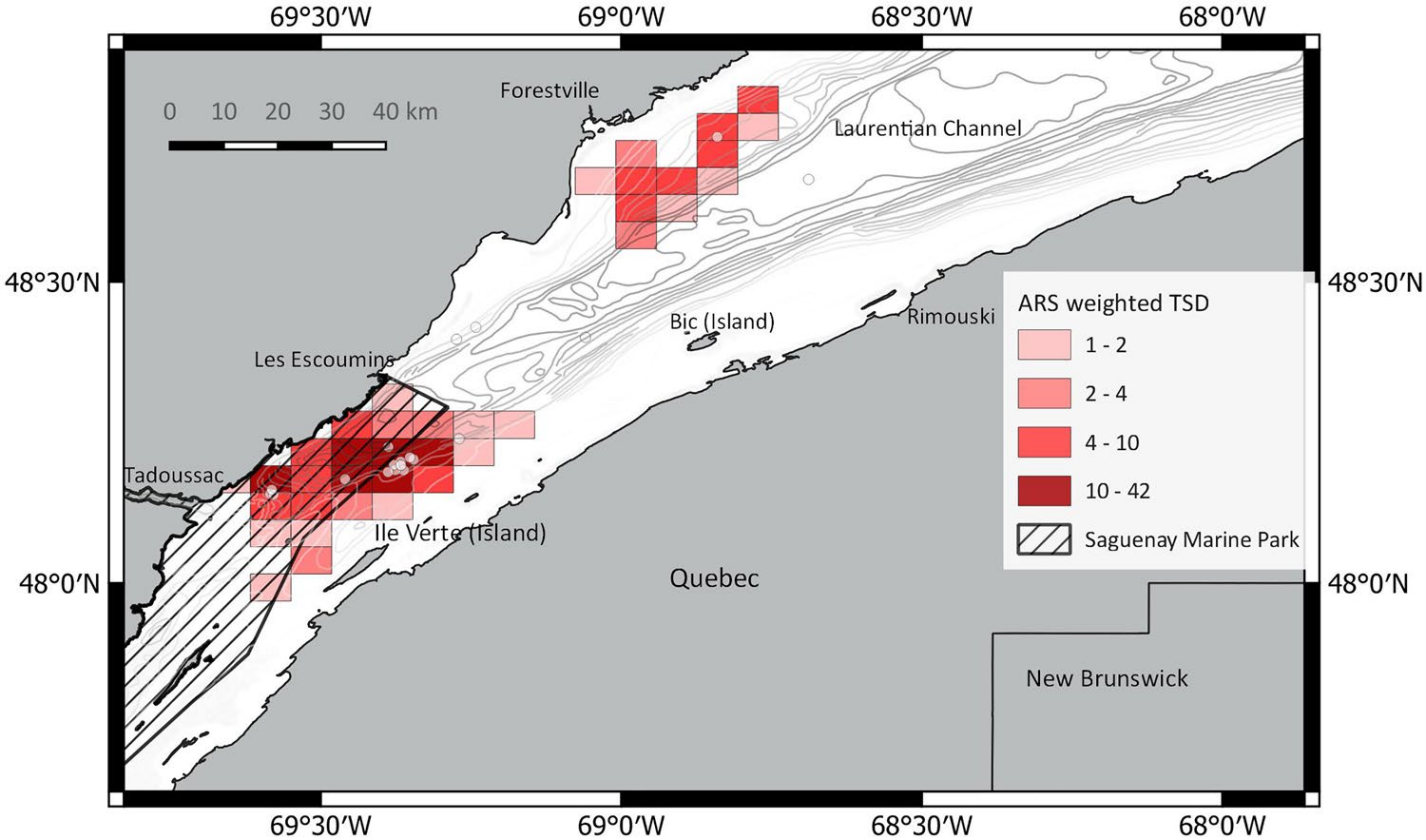
Area-restricted search (ARS; weighted TSD) count in the St. Lawrence Estuary, including the Saguenay St. Lawrence Marine Park (crossed line). ARS counts were made using a 5*5 km grid; counts ≤ 1 are not shown for clarity. White dots represent deployment sites (N=20).

In the Estuary and GSL, they included the head and slopes of the Laurentian Channel and the Shediac Valley (Figs. 3 and 4), whereas elsewhere, they included the continental slope and underwater seamounts (Fig. 4, Figs. IV–VI, Supplement 2, online).

Noteworthy is that ten whales conducted ARS in the northwestern Gulf to the West of Anticosti during summer, fall or winter, but we did not deploy any tag in that region. Weighing or not the ARS states using the time elapsed since deployment did not change the results (Fig. 3a versus b). Location or densities of predicted ARS states were also robust to the inclusion or exclusion of specific tags from the analysis (e.g., the five individuals leaving the shelf; results not shown).

The density of transit points along the south slope of the Laurentian Channel, both in the Estuary and GSL, and the occurrence of ARS behavior along this topographic feature suggest that this may represent a transiting corridor between the two areas (Fig. IX, Supplement 2, online).

## Discussion

We reported on habitat use by fin whales from the western North Atlantic and provided the first record of seasonal migratory movements of individual whales from this region. Movements of individual whales span the entire range of the species in the western North Atlantic, thereby challenging the current understanding of population structure. Our results also indicate high individual variability in migratory patterns over space and time and provide no support for the existence of a common migratory destination. In addition, the high occurrence of ARS behavior during early winter and its association with oceanographic (e.g., upwelling) and topographical features (e.g., near seamounts or the continental shelf edge) suggest continued foraging activity, at least by some individuals at and outside summer foraging grounds.

The International Whaling Commission (IWC) proposed separating fin whales in the North Atlantic into seven stocks^45^. This structure was based mainly on morphological measurements from whaling and captures of tagged whales and might represent more management units than biological entities^45^. The data now dates 100+ years, raising the question of its application to modern times, especially with recent environmental changes. Genetic studies distinguish between the Mediterranean Sea and the eastern and western North Atlantic populations but detected no substructure within the western North Atlantic^30,32^. The east-west divide was confirmed by early acoustic work^37^, leading to the suggestion that fin whale song structure might be a useful tool for assessing population structure at a finer scale^50,51^. A recent study provided evidence for the song structure of fin whales being different among three regions and stable over at least a few years: (1) the Gulf of Maine/Bay of Fundy/western Scotian Shelf, (2) the Gulf of St. Lawrence/eastern Scotian Shelf, and (3) the Grand Banks/Southern Labrador Shelf^49^. However, another study indicates that individual fin whales can vary their song structure within a season^52^, thereby shedding doubts on the reliability of song structure for distinguishing populations or tracking their seasonal movements^53^. Among our 28 fin whales satellite tagged in the GSL, none moved into the eastern GSL or Newfoundland and Labrador (NL) waters, providing support for fin whales in these waters representing a separate stock^1,22,45,48^.

Tracking seasonal movements of fin whales in the western North Atlantic has remained challenging, even with the most recent technology. The interpretation of latitudinal changes in call density obtained by passive acoustic monitoring (PAM) large-scale networks is limited by confounding factors such as seasonal changes in call rates see Ref.^35^. Acoustic detection of fin whales is based on detecting a 20-Hz pulse on arrays of acoustic recorders^33,35,39,54^. These calls are produced exclusively by males^55^. While they occur year-round, they are more common between August and April in the Northern Hemisphere, which coincides with the breeding season^33,54^, and become scarcer in the spring and summer^34,35,38^. While fin whale presence can be detected with PAM systems, current knowledge remains limited to distinguish among potential stocks, and assess their respective north-south movements.

Early studies relying on catch records and discovery tags suggested a synchronized movement among neighbouring sub-populations of fin whales in eastern Canada, cascading each other south during the fall and winter and in the opposite direction in the spring^1,22^. There was also a belief that fin whales from the GSL wintered outside but close to the GSL until ice disintegrated in the GSL the following spring^48^. PAM networks data tend to dismiss this hypothesis as they indicate a year-round presence of fin whales from the highest latitudes off western Greenland south to the Southeastern US, including the GSL^35,38,39^.

Our satellite telemetry data also does not support this hypothesis, as they show southerly movements by individual fin whales over and beyond the known distribution for the western North Atlantic population, reaching the proposed hiatus south of 30 degrees North^19,20,35^. These observations suggest that if a sub-structure existed within the western North Atlantic population, various sub-populations would likely co-occur at the same latitudes during their winter breeding/mating season. Three of our individuals tracked while migrating south in early winter returned to the shelf waters of Nova Scotia after 10–43 days. These observations would be consistent with the hypothesis of the GSL individuals remaining close to the GSL during winter. However, whether these whales eventually returned south in February or March cannot be ascertained. Such short excursions south in early winter, followed later by a second and more prolonged movement south, have been documented in blue whales from the Gulf of St. Lawrence^14^.

Our results suggest that the Scotian Shelf, including part of the Gulf of Maine, are an important wintering area for fin whales summering in the GSL. The occurrence of ARS behavior along topographical features suggests this area is likely used for foraging outside their traditional feeding season. These observations are consistent with earlier studies reporting fin whales feeding on herring (*Clupea harengus*) and Euphausiids off Nova Scotia from December to May^6^. Detecting the 20 Hz calls in this area during winter suggests possible mating and social interactions^35,39^. However, whether different sub-populations co-occur during winter is unknown; examining song structure or genetic distinction might help elucidate this question in the future.

Our study underscored a high variability in migratory patterns among individuals, with some animals remaining in northern waters and others undertaking more extensive seasonal movements. These findings are consistent with studies challenging the traditional view that, in fin whales and other baleen whales, all (most) individuals undergo a north-south migration between foraging and breeding grounds^11,25,35,36,56^. The lack of information on the sex, reproductive status and body condition of individuals and the absence of complete track records for the winter period for fin whales in our study made inferences about the reasons behind the observed variability in movement patterns among individuals challenging. However, the seasonal relative occurrence of transit and ARS behavior in specific geographic locations may provide insights into the motivations or objectives behind individual movement patterns.

The predicted ARS behavior decreased progressively from summer to fall, then winter, but still accounted for more than half the predicted behavior in December and January. However, the distinction between ARS and transiting in winter appeared more spatial than temporal. Animals still in the GSL and on the Continental Shelf at that time of year conducted mostly ARS, whereas animals that had ventured into the deeper Ocean exhibited almost exclusively transit behavior, except near underwater seamounts. Similar behaviors near these underwater structures were also observed in other species^14,57,58^. ARS behavior is often used as a synonym for foraging^12,25^, i.e. Ref.^59^, but see Ref.^60^, and the association of ARS behavior with topographical and oceanographic features or other areas of higher productivity, such as seamounts^14,57,58^ strongly support the occurrence of foraging in these areas. However, the slower speed and higher turning angle characterizing ARS can also represent social behaviors, such as mating and calving^57,61^. Therefore, we cannot exclude that mating and calving or other social interactions also occurred in these areas.

The high occurrence and persistence of these breeding calls at all latitudes in the western North Atlantic during the fall and winter suggest that a considerable proportion of western North Atlantic fin whales may be mating in the continental waters of eastern Canada and the northeastern United States^35,39^, including the southern GSL^38^. Singing fin whales were absent from the Caribbean Sea and the continental shelf in the southeast US^35^ but were recorded further offshore around Bermuda^37^.

Our telemetry study provides evidence for movements into these southern latitudes. However, the high variability in migratory patterns observed among individuals in time and space does not suggest any common destination, such as a distinct mating or calving ground. In humpback whales, ARS behavior is associated with breeding and occurs over scales of a few hundred square kilometres^58^. A similar study of fin whales in the northeastern Atlantic tracked the movements of a single individual from Svalbard south to Portugal and reported ARS behavior over a scale of a few thousand km^2^, over periods of generally less than a week^25^. We did not detect ARS patterns indicative of breeding activity in our study, similar to a blue whale study where GSL individuals ventured south in the same general sector as our fin whales^14^. The almost constant transiting state and high speed (at 6 to 10 km/h per day) of the four animals reaching these latitudes makes it unlikely that these few animals gave birth while in these waters. Whether mating occurred at these travelling speeds cannot, however, be ruled out, but it seems less likely.

Durban and Pittman^62^ suggested a less traditional reason for rapid large-scale movements in a study where they reported a fast turn-around trip from the Antarctic to subtropical waters by killer whales (*Orcinus Orca*). They suggested these incursions were associated with physiological maintenance (skin regeneration) and were conducted in warmer waters to avoid heat loss. In other species, such as pinnipeds and beluga (*Delphinapterus leucas*), an increase in skin temperature was shown to stimulate the moult^63,64^. Diatoms and ectoparasites grow and disappear on the skin of baleen whales in different habitats and temperatures^5,65,66^. However, it remains unknown whether skin regeneration could trigger the large-scale and short-duration southerly movements observed in some individuals during early winter in our study.

The southerly excursions documented in early winter past Bermuda and into the West Indies are similar to the general flow of movements for this species in the western North Atlantic^37^. PAM data confirm that fin and blue whales are present throughout the Canadian and US continental shelf south to 35°N from November to April and are rare beyond these latitudes^35,39^.

Our study confirmed the importance of the Estuary and GSL as foraging areas for fin whales. While our data was inadequate to assess the importance of these areas in spring, they suggest that foraging could occur in these areas until late fall and, in some years, into at least early January. The persistence of fin whales in the same restricted area in the Estuary for several weeks /months, combined with the short excursions out in the northwestern Gulf in some of the tagged whales, with a return to the tagging site, and the absence of fin whale aggregation elsewhere in the western GSL that year, indicate that in some years, adequate food supply may be limited to only a few areas. Human activity interfering with foraging in these areas might strongly impact the whales’ capacity to accumulate energy reserves, as demonstrated by blue whales in the St. Lawrence Estuary^67^.

Whether the persistence of fin whales in the Estuary and GSL, and at other high latitude foraging sites in the western North Atlantic e.g., Refs.^35,39^ is a consequence of recent warming conditions or is a population trait is unknown. Fin whales in our study were all tagged post-2010, i.e., after persistent record warming was observed each year for the Estuary and GSL^68^. In previous decades, fin whales would have been likely displaced out of the GSL by the extended pack ice coverage see Ref.^48,68^. Since 2010, a decline has also been noted in the abundance of fin whales in the GSL, concurrent with a decline in recruitment until at least 2016^69^. As in North Atlantic Right Whales, a decrease in body condition may increase the calving interval^70,71^. In conditions with limited food supply, migratory patterns of all population segments might be affected.

The comparison with previous studies poses a new challenge: how animals adapt or alter their migration strategies in a changing environment. Parts of the western North Atlantic are among the fastest-warming bodies of water in the world^72–74^. The resulting sea ice loss is already associated with an earlier arrival of 30 days (over 27 years) of fin whales in the GSL. The departure shifted forward less drastically, the animals spending more than two weeks longer in the northern GSL than three decades ago^75^. Fin whales have been recorded year-round in Davis Strait between Greenland and Canada during recent PAM studies^35^, and satellite telemetry data suggests a similar pattern from fin whales near Svalbard^25^. These two areas were likely ice-covered in winter during the peak whaling period, which raises the question about the current and future need of fin whale to migrate from these high-Arctic regions. These observations might suggest that the adaptation of large predators to climate change is likely already affecting individual and population-wide migration strategies, with potential incidence on population structure. Thus, even if the historically proposed stock structure based on whaling data were correct, its applicability under the new climate regime is questionable. What will happen if the GSL becomes ice-free year-round remains unknown. The need to migrate due to sea ice might cease while, at the same time, the availability of prey might also change and force whales to forage further afar. While fin whales are known to vary greatly in their individual migration strategies, and our study provides further evidence of this, likely as a result of reproductive and physiological requirements, continued change in migratory patterns are expected in response to environmental changes, especially in regions where ice as a physical barrier will disappear. This flexibility in migration is making fin whales somewhat resilient towards the current environmental changes.

## Material and methods

### Fieldwork and tag programming

**Figure 5:**
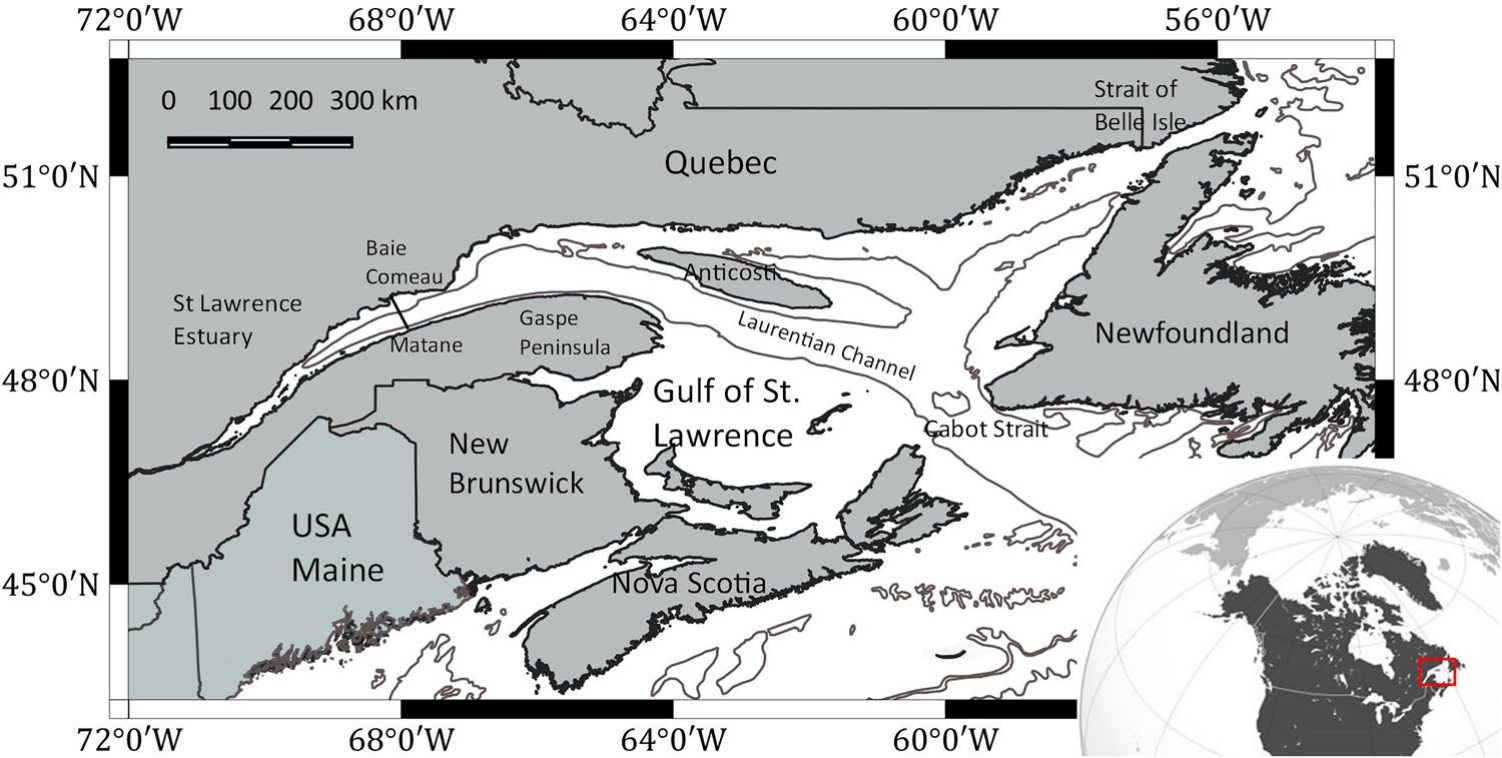
The study area, the Gulf of St. Lawrence, and its location within North America. Fall deployments were exclusively done in the Estuary (black dots, N=20), while summer deployments occurred in the Gulf (white dots, N=8).

We deployed 25 SPOT (2014 to 2017, 2020, 2021) and 7 SPLASH (2021) satellite-linked platform terminal transmitters (PTT or tags, Wildlife Computers, Redmond, WA, US) on fin whales from different locations in the Estuary and the Gulf of St. Lawrence. Four tags provided less than 10 positions, leaving 28 PTTs for data analyses (Fig. 1, Table A Supplements 2, online). Twenty of these 28 PTTs were deployed in the St. Lawrence Estuary during the fall (September to November), with eight other PTTs being deployed during summer (June to August) in the northwestern Gulf of St. Lawrence (Fig. 5). We shot tags at the base of the dorsal fin using a CO_2_-powered (21—23 bars) rifle (DanInject, JM Special 25 model, Børkop, Denmark) from 6—9 m rigid-hulled inflatable boats and a distance of 5—7 m from the whale using the best practice approach by Andrews et al.^76^. Both tag types used the LIMPET anchoring system consisting of two titanium barbs of 68 mm, each fitted with six backward-facing petals (Wildlife Computers, Redmond, WA).

The SPOT tags very short transmissions provide a position via the Doppler effect using the ARGOS system. In contrast, the SPLASH tags transmissions are longer and contain information such as GPS positions and behavior while still using the Doppler effect to estimate positions using the ARGOS system. We allowed a maximum of 250 daily ARGOS uplinks for the SPOT tags but increased the daily allowance to 500 for SPLASH tags to obtain the additional data. We programmed the GPS to acquire two positions per hour and adapted transmission hours to ARGOS satellite coverage (Fig. II, Supplement 2, online) to preserve battery energy. We used a repetition rate for successive transmissions of 15 s to maximize transmission opportunities during a satellite pass.

ARGOS categorizes the estimated positions according to the number of uplinks received by satellites and the time elapsed between messages/uplinks. ARGOS positions have 7 location classes (lower to higher precision): Z, B, A, O, 1, 2, and 3. Following other studies, we deleted class Z locations^14,25,77^.

### Model

We applied a Bayesian hierarchical switching state-space model (hSSSM) to our ARGOS plus GPS satellite data using the R (4.1.1. R-Core Team 2021) package ‘bsam’^78–80^. The hSSSM couples two stochastic equations to estimate hidden behavioral states and movement parameters from animal movement data^79^. The first (process) equation uses a first-difference correlated random walk approach (DCRW) to estimate the future state of an individual, given its current and previous state^78^. The model applies a user-defined time step, which should correspond to the resolution of the raw data, i.e. the time between successive positions^78^. The DCRW can switch between two discrete behavioral modes based on the parameter move persistency (γ, autocorrelation in speed and direction). The two behavioral states are transiting and area-restricted search (ARS). Transiting animals are expected to move at higher speeds and display small movement angles (directional movement) compared to animals in ARS, which should typically show lower speeds and higher turning angles. As a result, the autocorrelation (γ) among successive locations should be closer to one for transiting individuals and closer to zero for animals in ARS^78,79^. The second (observation) equation relates the unobserved location states predicted by the process equation to the observed positional data (ARGOS/GPS) over the same time step.

The SSSM assesses only two behavioral modes (1 = transiting and 2 = ARS), but averaging the MCMC samples across chains returns continuous values between 1 and 2. Following numerous studies^12,14,59,79^, we considered a mean behavioral mode > 1.75 to represent ARS behavior and a mean value < 1.25 to represent transiting. We regarded values between 1.25 and 1.75 as uncertain.

We simultaneously fitted the SSSM as a single hierarchical (hSSSM) model to all tracks^80^. The tracks included ARGOS and GPS positions because we did not receive many GPS positions (Table 1). We treated GPS positions as ARGOS class 3 positions. The hierarchical framework assumes that individuals are samples from the same population and that their movement and behavioral parameters come from a common distribution. Additionally, hSSSM enables parameter estimation of shorter tracks by pooling them with other tracks.

Data span the period from June to January. Movement between foraging sites during the summer might be slower and less direct than during the generally assumed north-south migration in fall-winter, resulting in different estimates of γ (autocorrelation). Specifically, rapid winter migration may skew the persistency of transiting behavior upward and thus could lead to some summer directional movements not being recognized. To examine this potential bias, we divided the dataset into three seasonal components: summer (June, July, August), fall (September, October, November) and winter (December, January). We ran the hSSSM model on each part separately and compared the results with the model using the entire dataset.

The package ‘bsam’ fits the hSSSM by running two parallel Markov Chain Monte Carlo (MCMC) chains via JAGS (Just Another Gibbs Sampler, https://mcmc.-jags.souceforge.net), each comprised of 180,000 iterations. We discarded the first 100,000 as a burn-in for each chain and retained every 16^th^ sample of the remaining 80,000 of each chain to reduce autocorrelation, resulting in a posterior distribution of 5000 samples. We visually assessed plots for MCMC chain convergence, posterior density function and autocorrelation of each estimated parameter (Supplement 3, online).

### Time-step and time-gap

The chosen time step of the model must match the average time difference between actual locations^78^. Plots of time elapsed between locations and the number of locations per day for each tag revealed two larger gaps and two peaks in ARGOS satellite coverage over the Gulf of St. Lawrence (Supplement 1, Figs. I and II Supplement 2, online). Many intervals were around 12 hours, followed by multiple positions in rapid succession during peak satellite coverage. Based on these observations, we chose 12 hours as the appropriate time step for our dataset and not the mean or median time difference because the latter would infer a finer resolution than justified by the data.

ARGOS transmissions can include gaps of multiple days between two successive locations. The model estimates behavioral modes and locations according to the chosen time step, regardless of the gap length. One approach to avoid overfitting is to split an animal track into multiple segments and treat them as separate tracks^14,25^. To keep a maximum of information while reducing the uncertainty for the days without position data, tracks with data gaps > 3 days were split into two or more sub-tracks, leading to 36 tracks from the 28 individual whales to identify ARS behavior in the Estuary and Gulf of St. Lawrence, i.e., prior to the departure date (see below). For the estimation of migration parameters (see below), we did not split the tracks.

### Departure date (beginning of migration)

Departure from the Estuary and Gulf of St.Lawrence was determined as the first estimated transiting state/location that crossed a line between Baie Comeau and Matane and between Nova Scotia and Newfoundland in the Cabot Strait, respectively (Fig. 5). We defined the start of the migration behavior as for blue whales from the Gulf of St. Lawrence^14^, i.e., > 48 h of consecutive transiting behavior (b < 1.25), but we altered it to the first 48 h after the last ARS site (location) within the GSL.

We calculated the mean swimming speed (km/h) for each behavior mode across all animals by dividing the distance (in km) between two successive estimated hSSSM locations of the same state by the time step (12 h). We also calculated the total distance travelled per individual, per day, and behavioral state. We used the R package ‘Geosphere’ to calculate the distance between two predicted states/locations^81^.

### Areas of restricted search

We plotted the ARS states provided by the hSSSM model using QGIS 3.22.0 (Coordinated Reference System WSG 84, EPSG 4326). We could not follow several studies defining ARS patches as three consecutive locations in ARS behavior mode^14,25,59,77^ due to our prolonged (12-h) time step between estimated locations. Limiting ARS patches to areas where fin whales spent 36 hours (3 * time step) would potentially miss many potential foraging areas.

We applied a 10*10 km grid over our study area and tallied the ARS states of all tracks per grid cell. Grid cells near the deployment sites were likely to include more ARS states than grid cells at greater distances, potentially underestimating the importance of some areas. We conducted a sensitivity analysis and weighted each ARS state from the first ten days following the deployment by the time since deployment (TSD). Based on the average swim speeds of fin whales, we assumed that an animal could easily move from one grid cell to another in a single time step (12 h) or exit the Estuary or wider foraging regions in the GSL within ten days. Thus, we assumed that predicted ARS states later in the deployment were increasingly less biased towards the deployment site. We weighted ARS locations of the first day by a factor of 0.1, the second day by 0.2, etc. and counted ARS states after nine full days. We reduced the grid to 5*5 km cells for the St. Lawrence Estuary to investigate fine-scale movements within this area.

### Ethics statement

The study was conducted under research permits from the Department of Fisheries and Oceans Canada (DFO) and Parcs Canada. The Canadian Animal Care Council sub-committee of the Maurice Lamontagne Institute (DFO), Mont-Joli, QC, approved tagging procedures per all national guidelines and regulations. All authors complied with the ARRIVE guidelines.

### Supplementary Information

Supplementary Information 1.

Supplementary Information 2.

Supplementary Information 3.

Supplementary Information 4.

Supplementary Information 5.

Supplementary Information 6.

## Data Availability

All data generated or analysed during this study are included in this published article [and its supplementary information files].
