# Supplementary material for: Migratory movements of fin whales from the Gulf of St. Lawrence challenge our understanding of the Northwest Atlantic stock structure
Source: Sci Rep. 2024 May 20;14:11472. doi: 10.1038/s41598-024-62173-1 (PMC11106244; doi:10.1038/s41598-024-62173-1)
Supplement: Supplementary file 6 — Supplementary Information 6. [file 41598_2024_62173_MOESM6_ESM.pdf]

Supplement material for:

Migratory movements of fin whales from the Gulf of St. Lawrence, Canada, challenge our understanding of the Northwest Atlantic stock structure.

Supplement 1

Number of satellite-derived positions over the 24 hours and time difference between subsequent positions for all tags.

C Distribution of the locations per hour

2014_106740

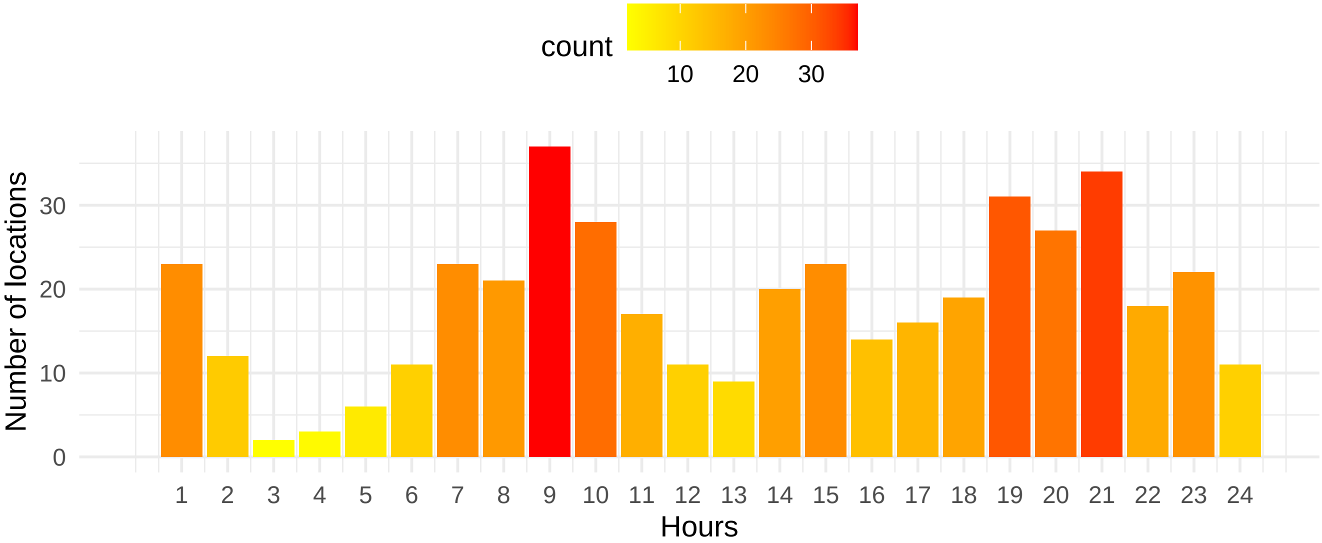

D Time interval between locations since deployment

2014_106740

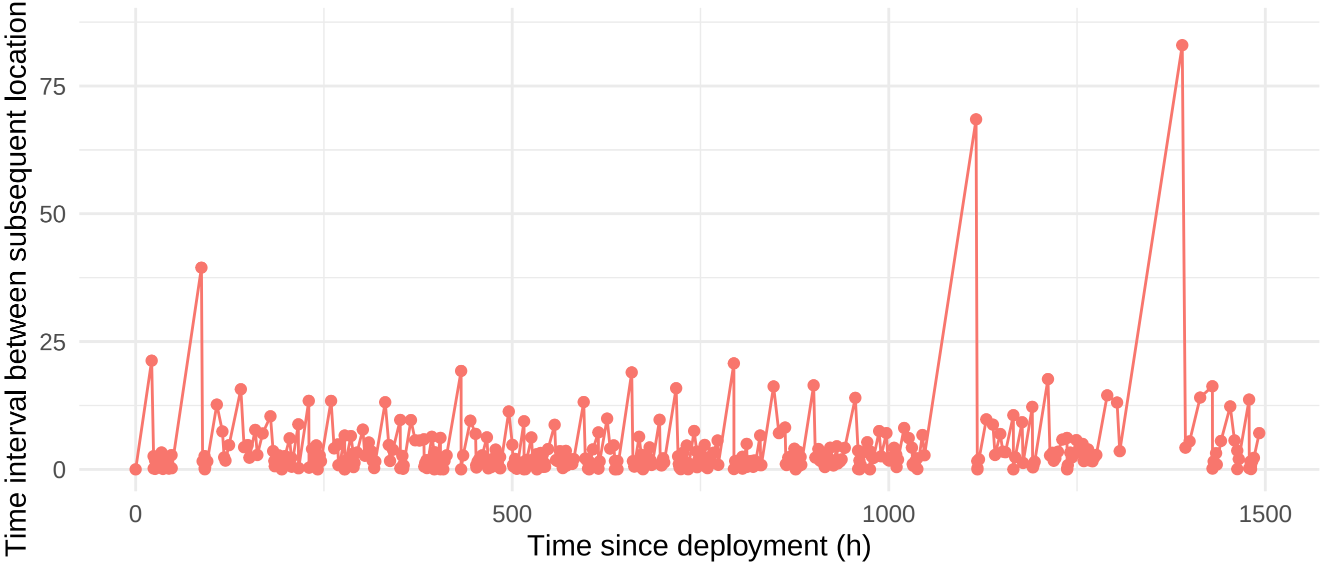

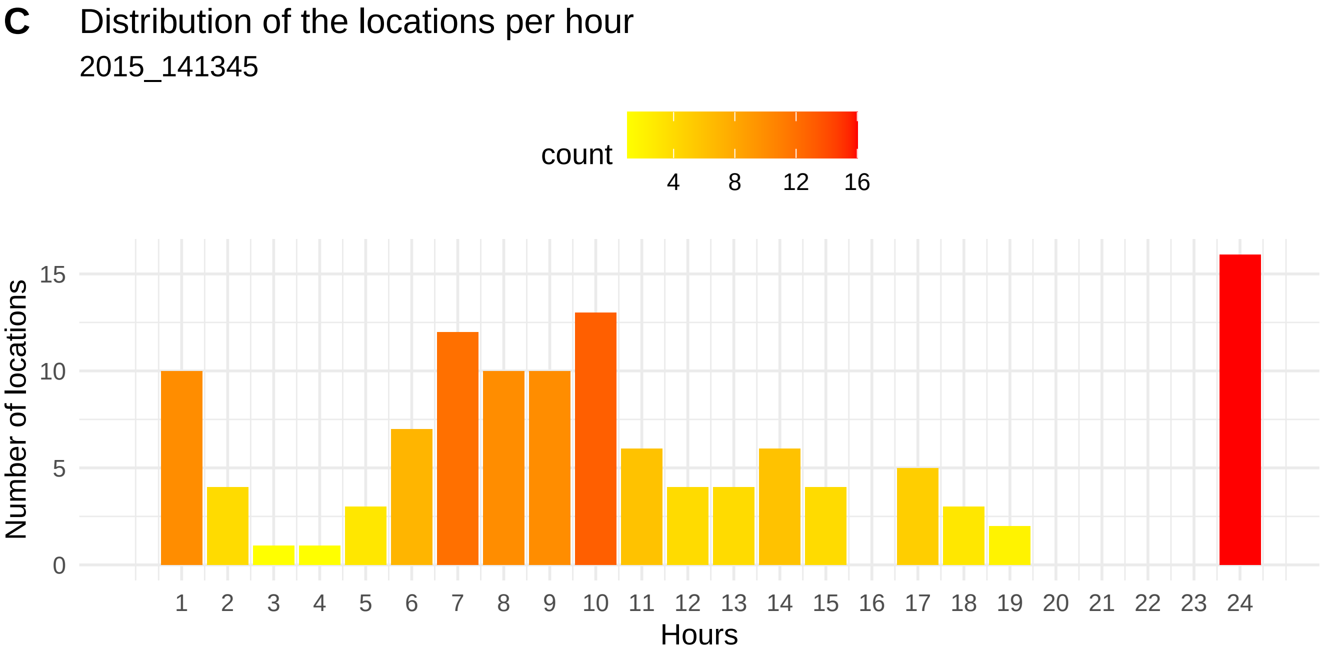
C
Distribution of the locations per hour
2015_141345
count
4
8
12
16
Number of locations
0
5
10
15
1
2
3
4
5
6
7
8
9
10
11
12
13
14
15
16
17
18
19
20
21
22
23
24
Hours

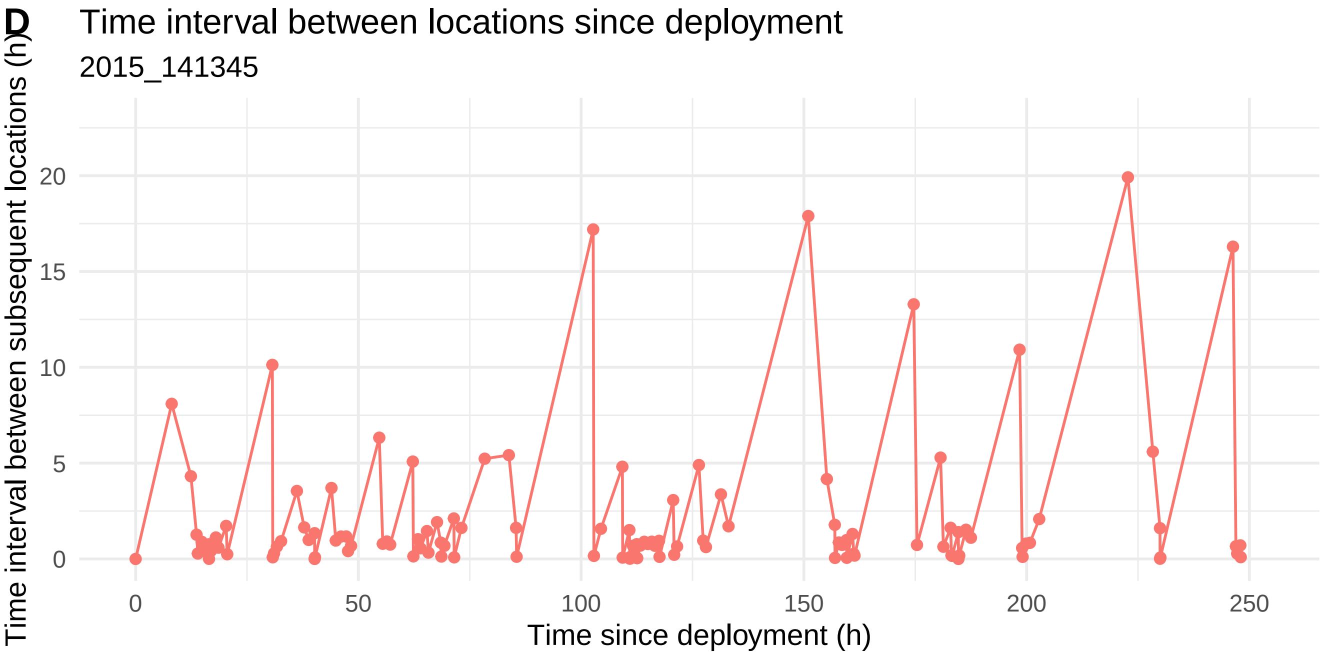
D
Time interval between locations since deployment
2015_141345
Time interval between subsequent locations (h)
0
5
10
15
20
0
50
100
150
200
250
Time since deployment (h)

C Distribution of the locations per hour

2015_141347

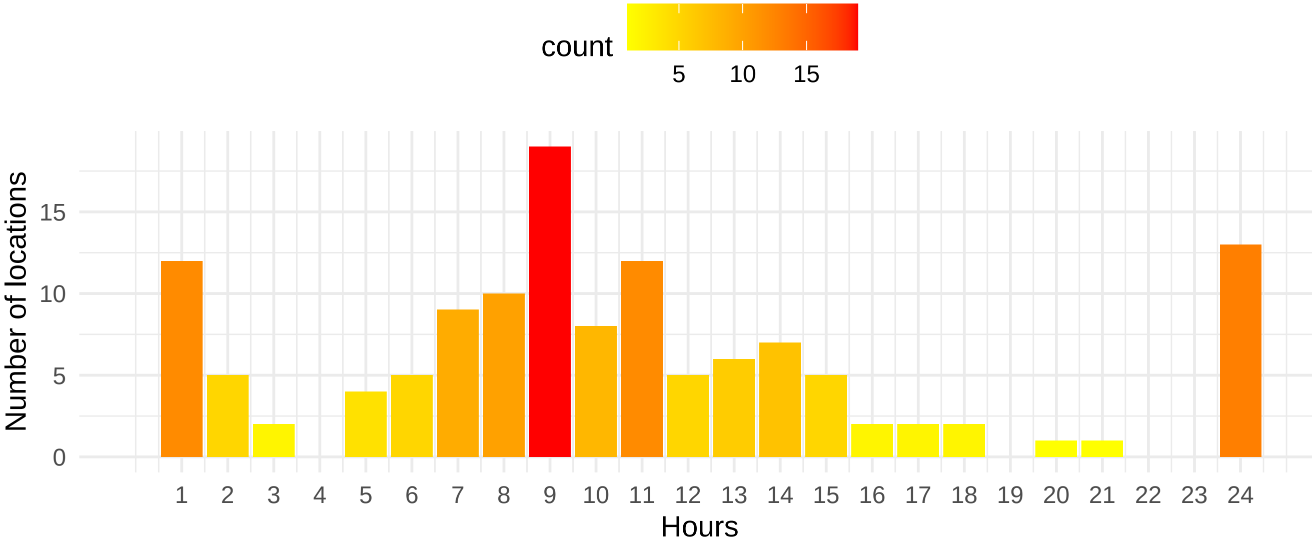

D Time interval between locations since deployment

2015_141347

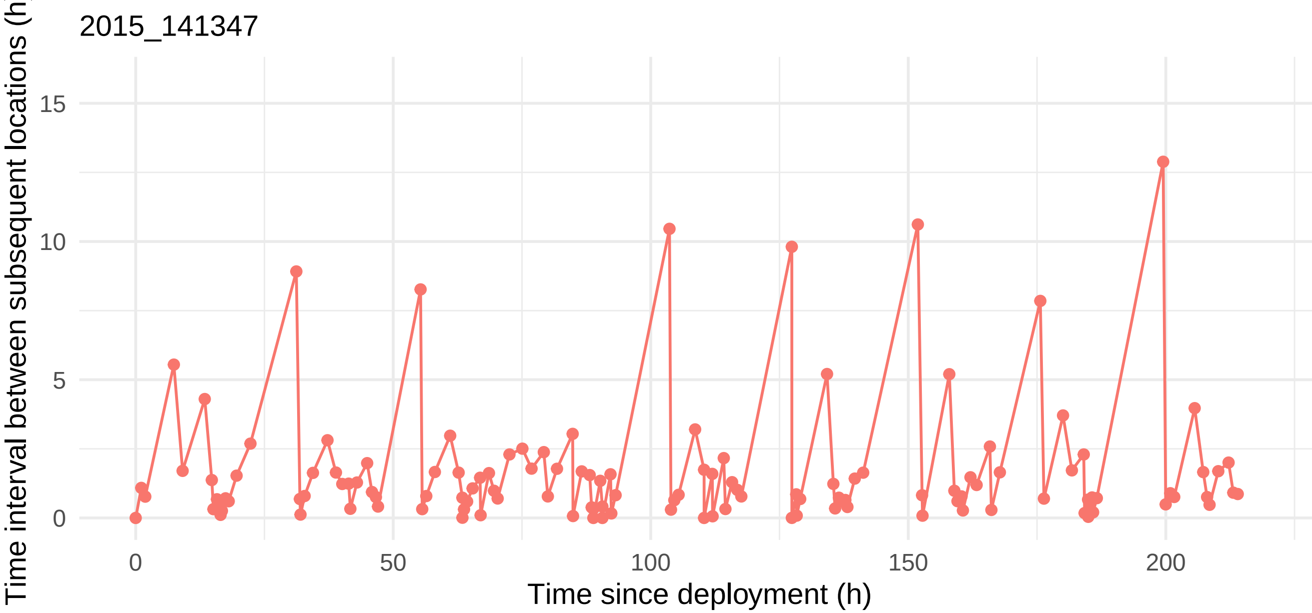

C

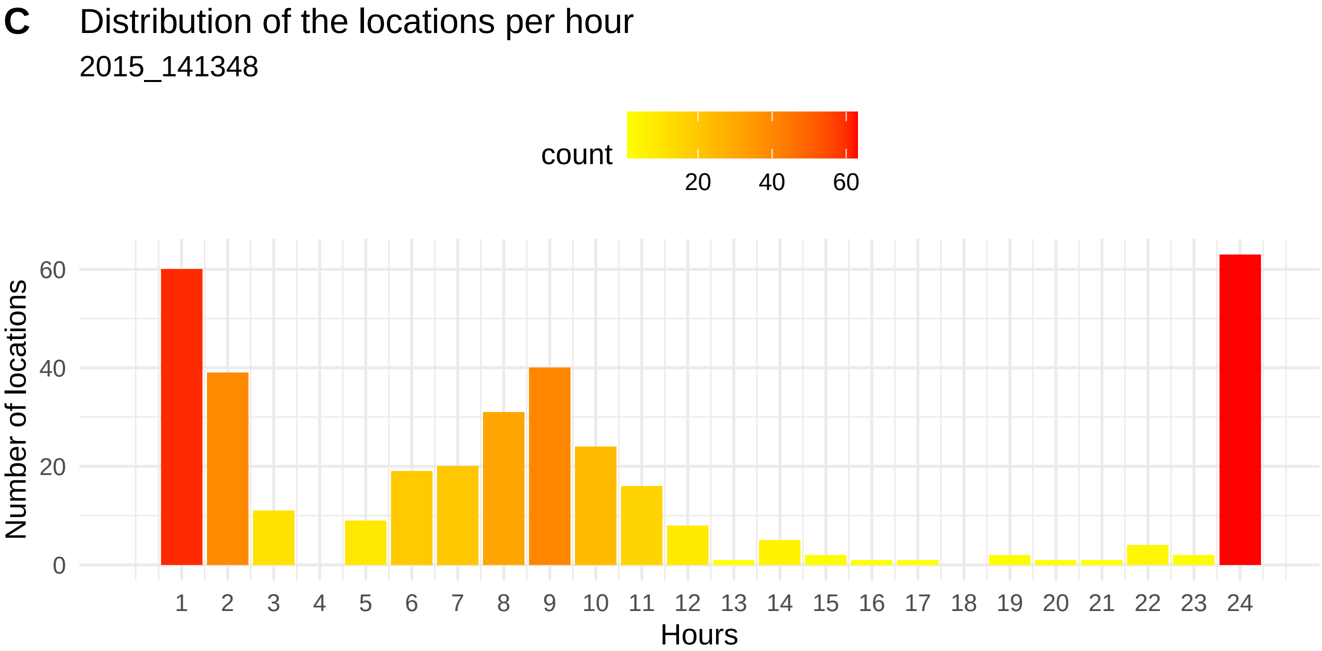
Distribution of the locations per hour
2015_141348
count
20
40
60
Number of locations
0
20
40
60
1
2
3
4
5
6
7
8
9
10
11
12
13
14
15
16
17
18
19
20
21
22
23
24
Hours

D

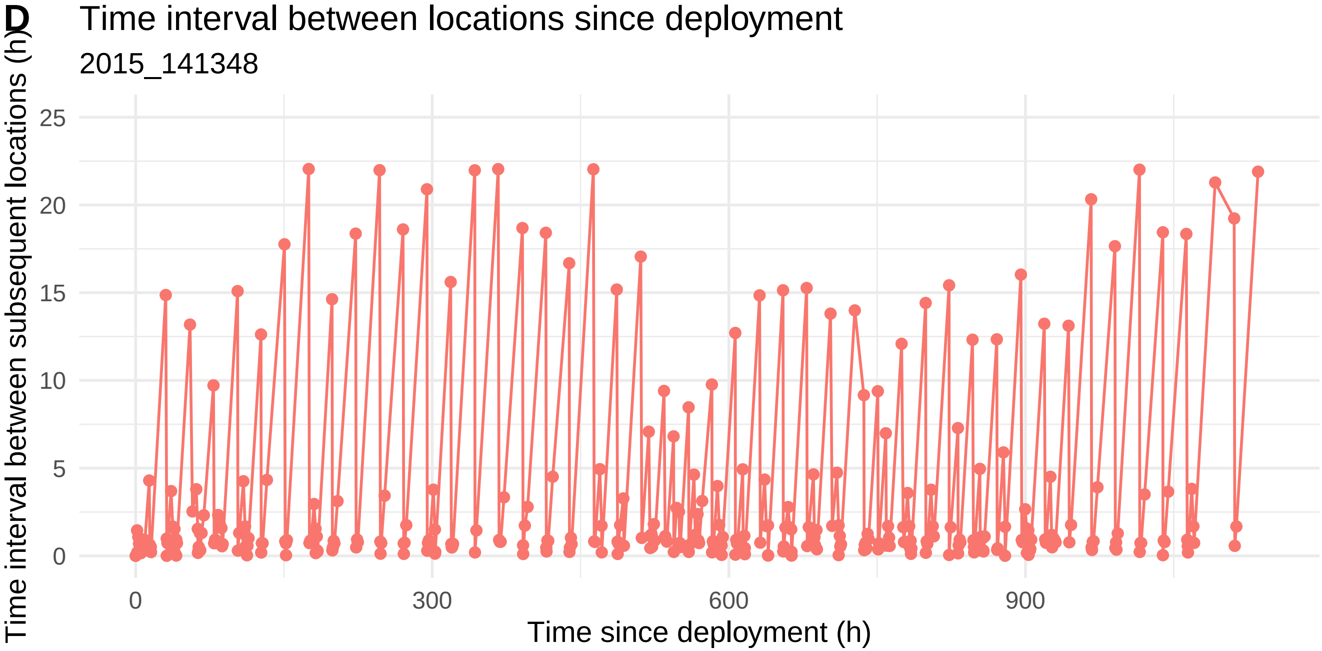
Time interval between locations since deployment
2015_141348
Time interval between subsequent locations (h)
0
5
10
15
20
25
0
300
600
900
Time since deployment (h)

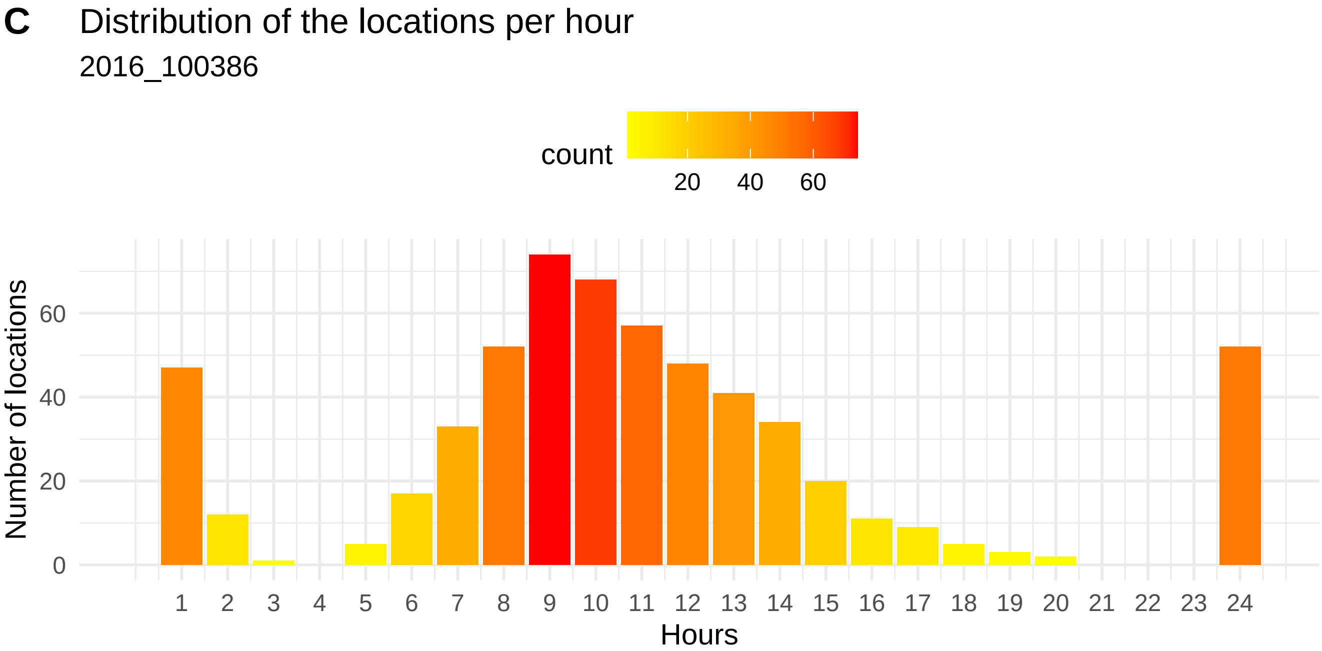
C
Distribution of the locations per hour
2016_100386
count
20
40
60
Number of locations
0
20
40
60
1
2
3
4
5
6
7
8
9
10
11
12
13
14
15
16
17
18
19
20
21
22
23
24
Hours

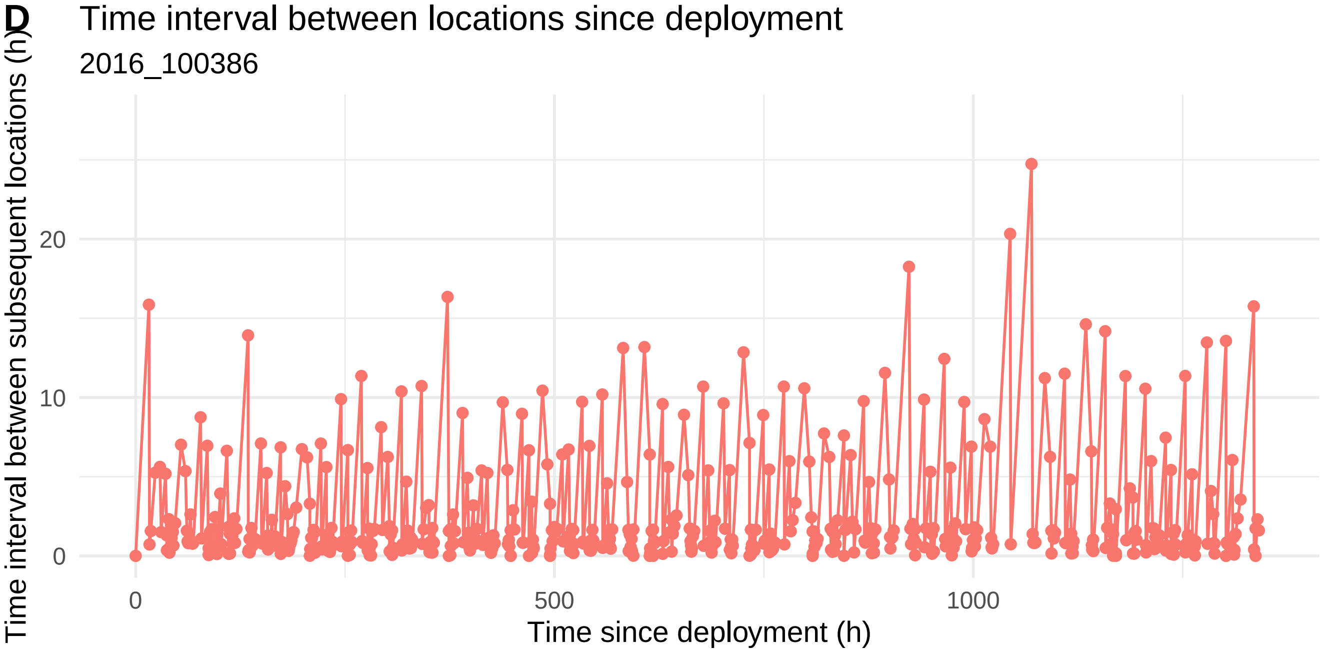
D
Time interval between locations since deployment
2016_100386
Time interval between subsequent locations (h)
0
10
20
0
500
1000
Time since deployment (h)

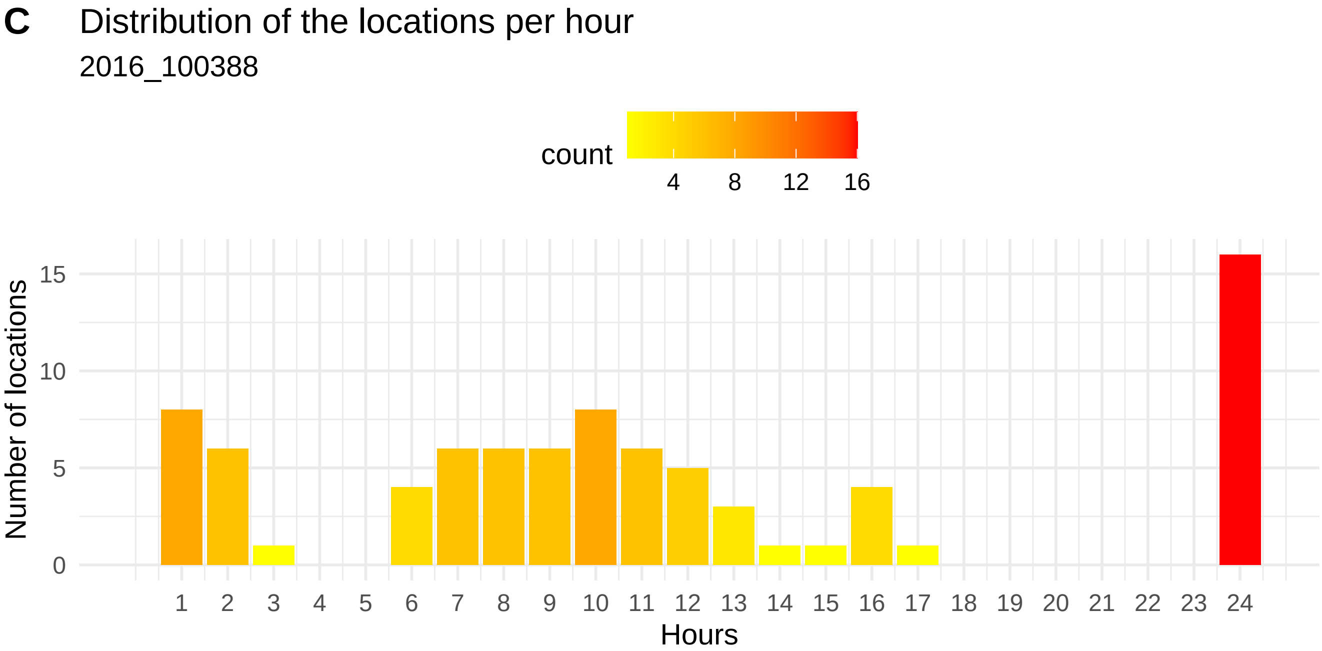
C
Distribution of the locations per hour
2016_100388
count
4
8
12
16
Number of locations
0
5
10
15
1 2 3 4 5 6 7 8 9 10 11 12 13 14 15 16 17 18 19 20 21 22 23 24
Hours

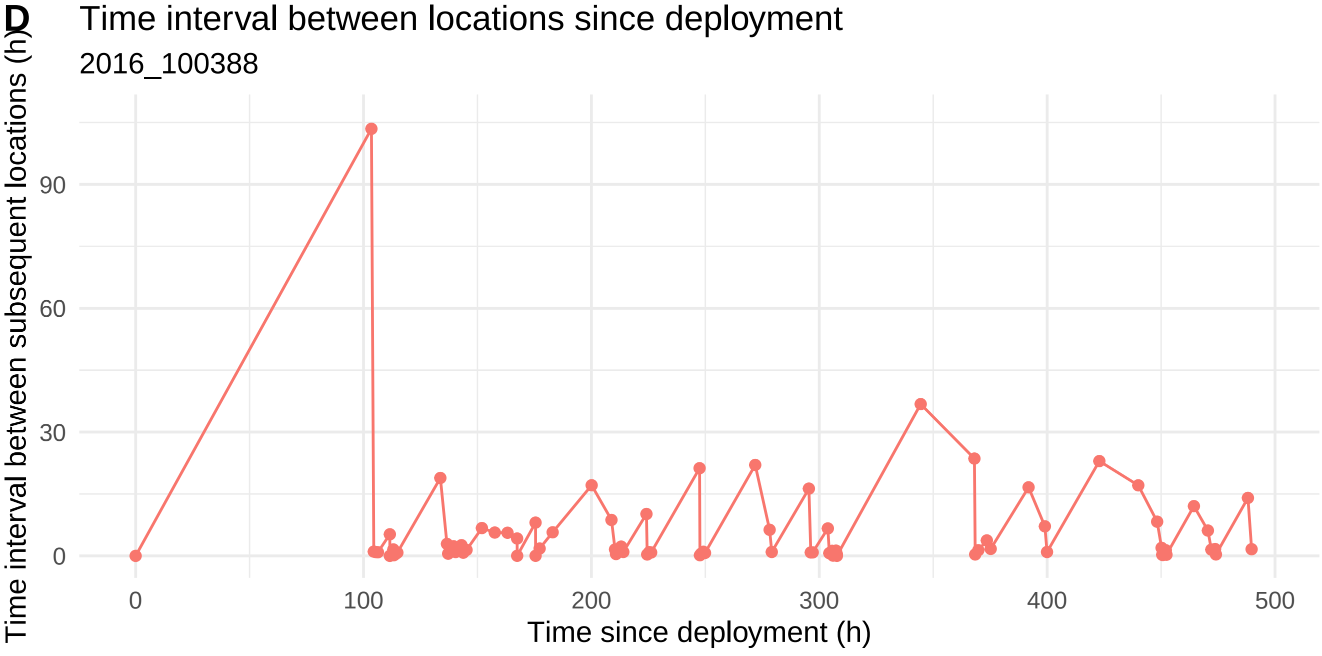
D
Time interval between locations since deployment
2016_100388
Time interval between subsequent locations (h)
0
30
60
90
0
100
200
300
400
500
Time since deployment (h)

C Distribution of the locations per hour
2017_100383

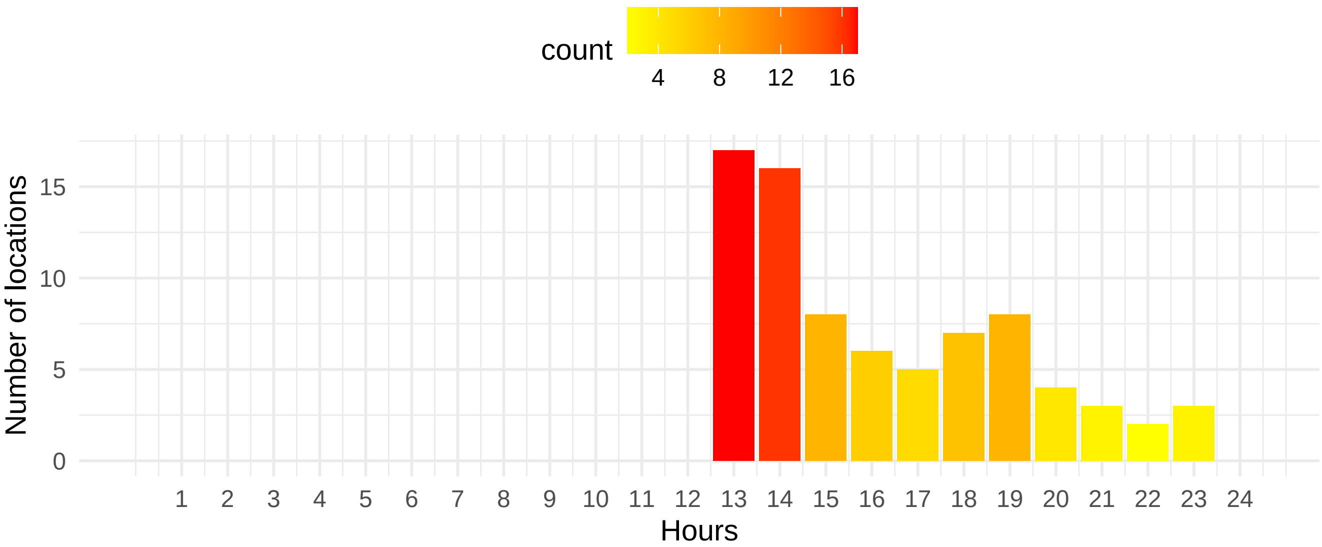

D Time interval between locations since deployment
2017_100383

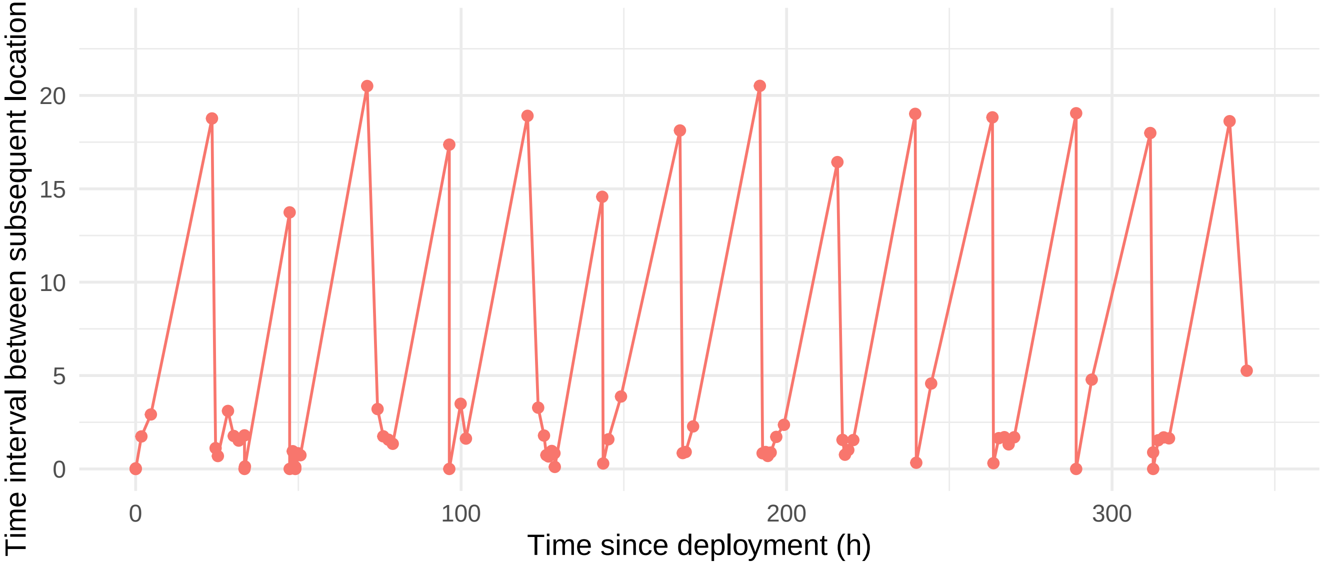

C

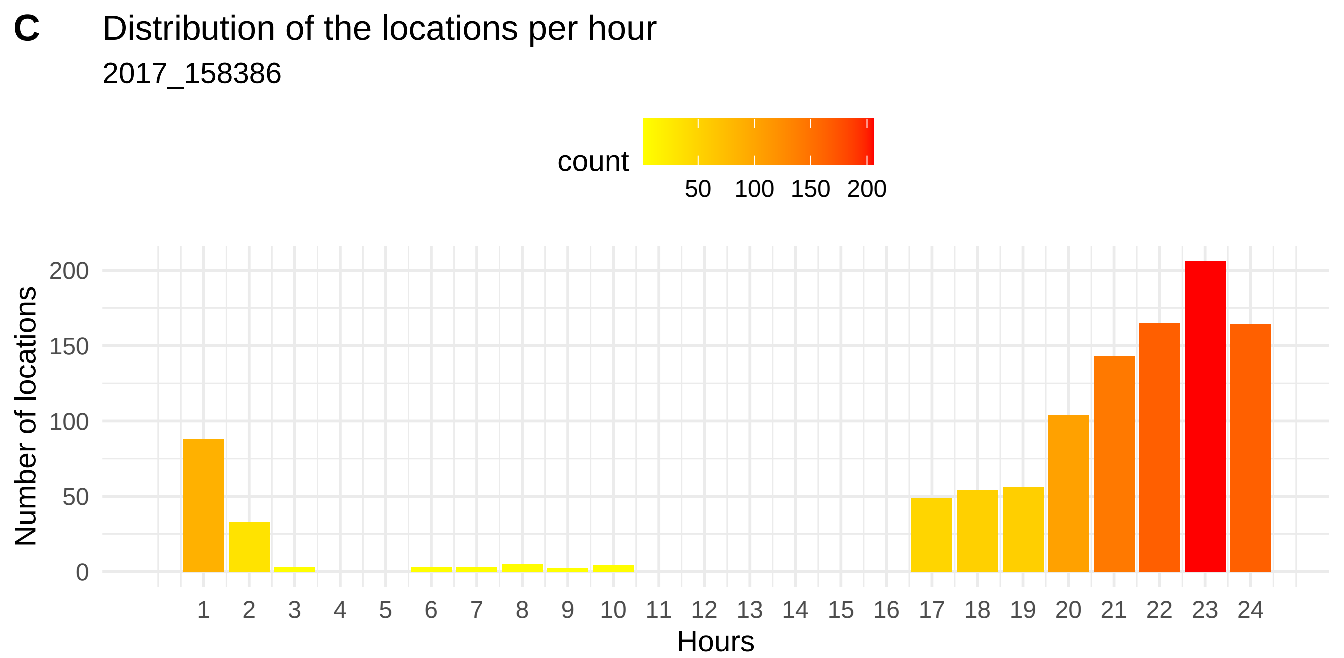

Distribution of the locations per hour
2017_158386
count
50
100
150
200
Number of locations
0
50
100
150
200
1 2 3 4 5 6 7 8 9 10 11 12 13 14 15 16 17 18 19 20 21 22 23 24
Hours

D

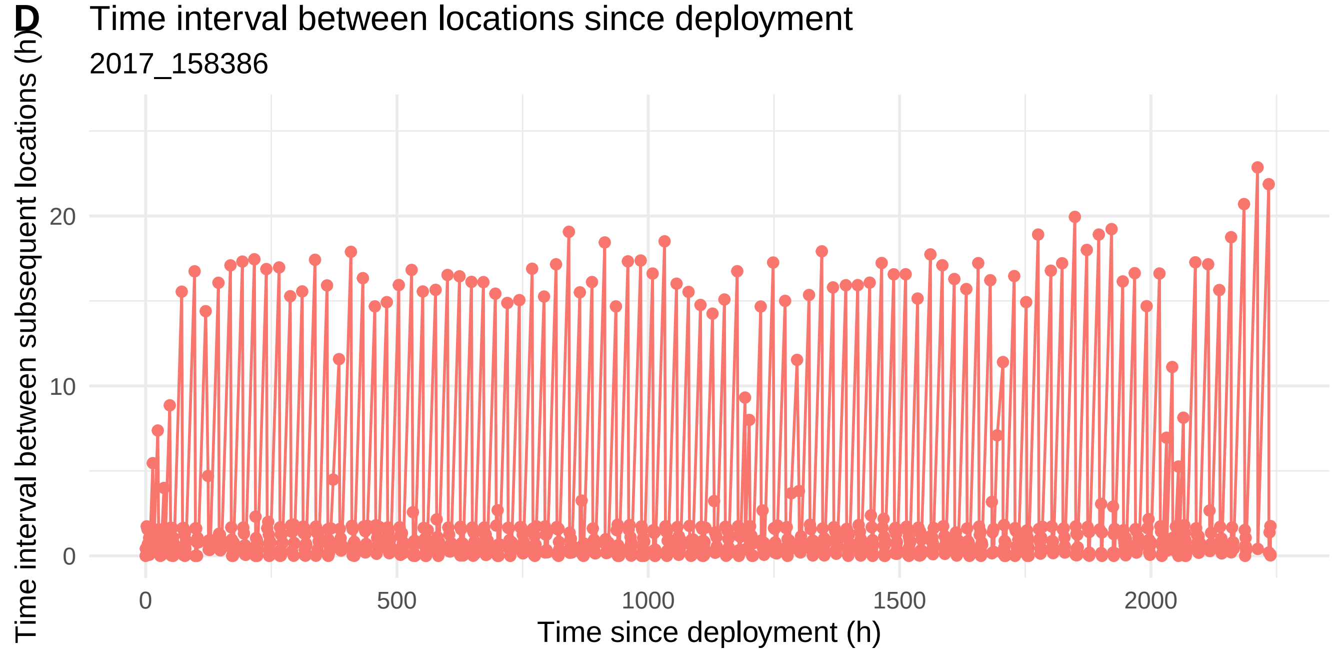

Time interval between locations since deployment
2017_158386
Time interval between subsequent locations (h)
0
10
20
0
500
1000
1500
2000
Time since deployment (h)

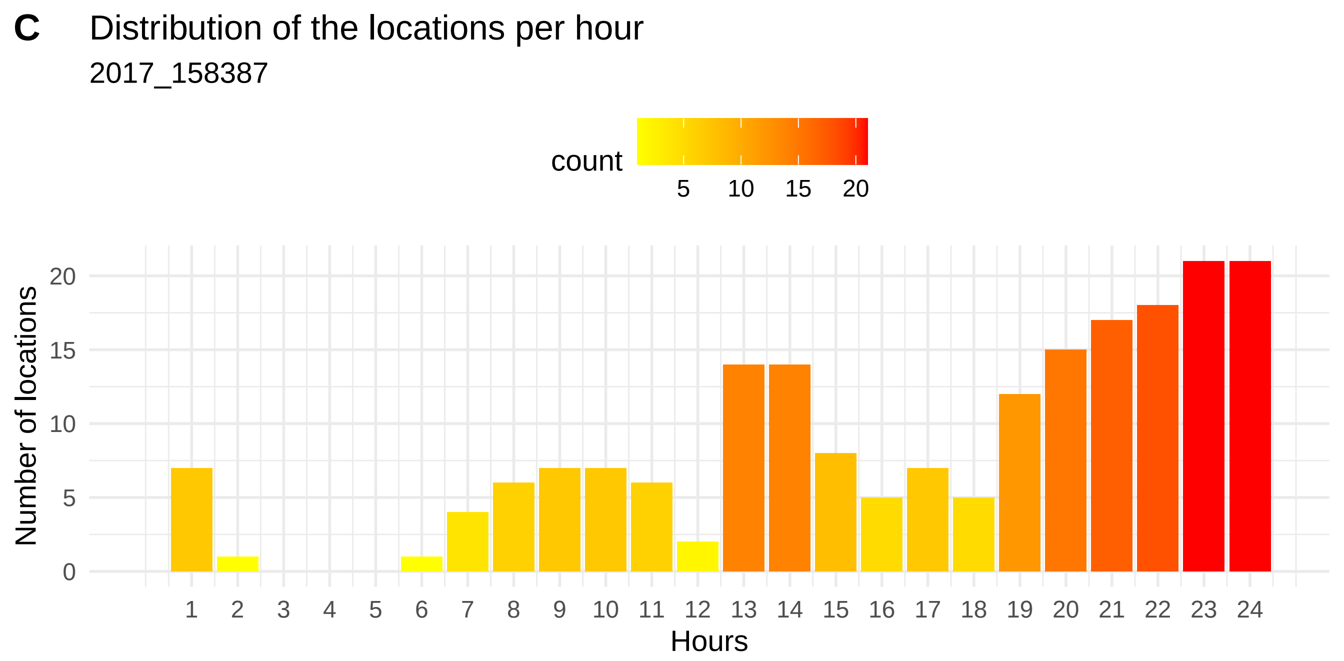
C
Distribution of the locations per hour
2017_158387
count
5 10 15 20
Number of locations
0 5 10 15 20
1 2 3 4 5 6 7 8 9 10 11 12 13 14 15 16 17 18 19 20 21 22 23 24
Hours

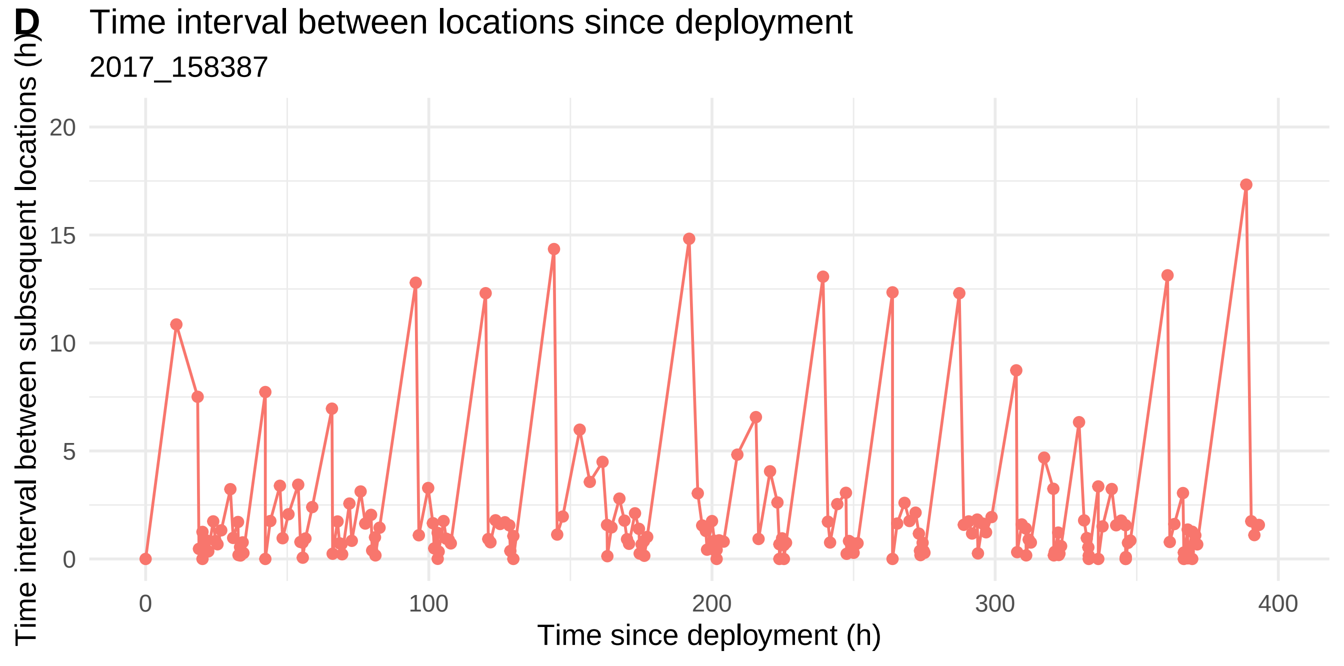
D
Time interval between locations since deployment
2017_158387
Time interval between subsequent locations (h)
0 5 10 15 20
0 100 200 300 400
Time since deployment (h)

C Distribution of the locations per hour

2018_172790

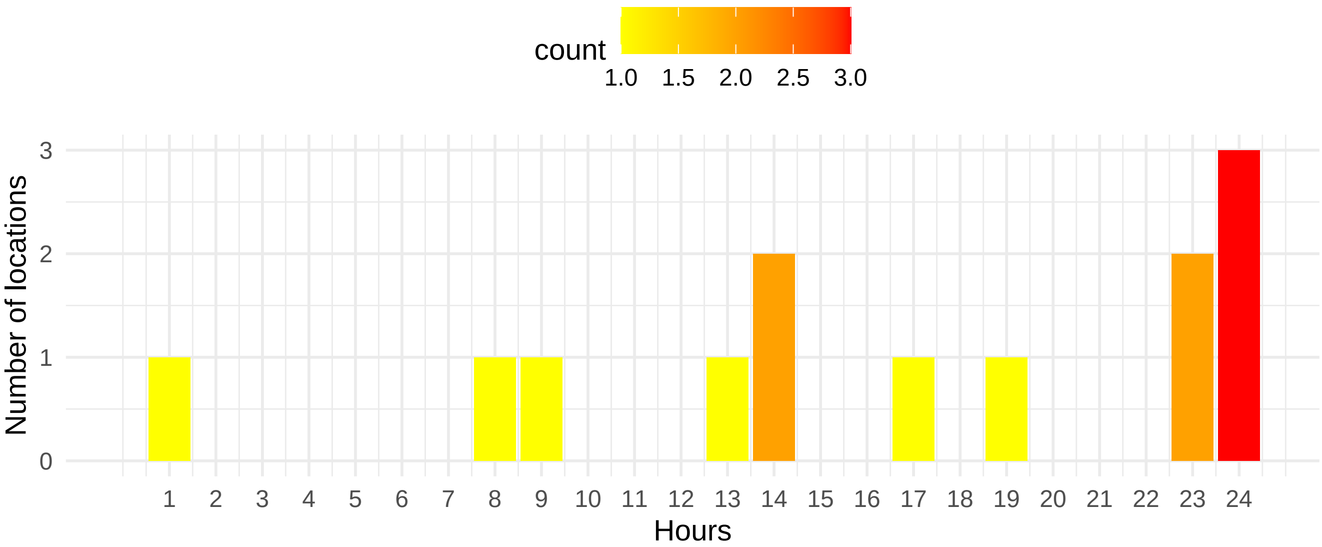

D Time interval between locations since deployment

2018_172790

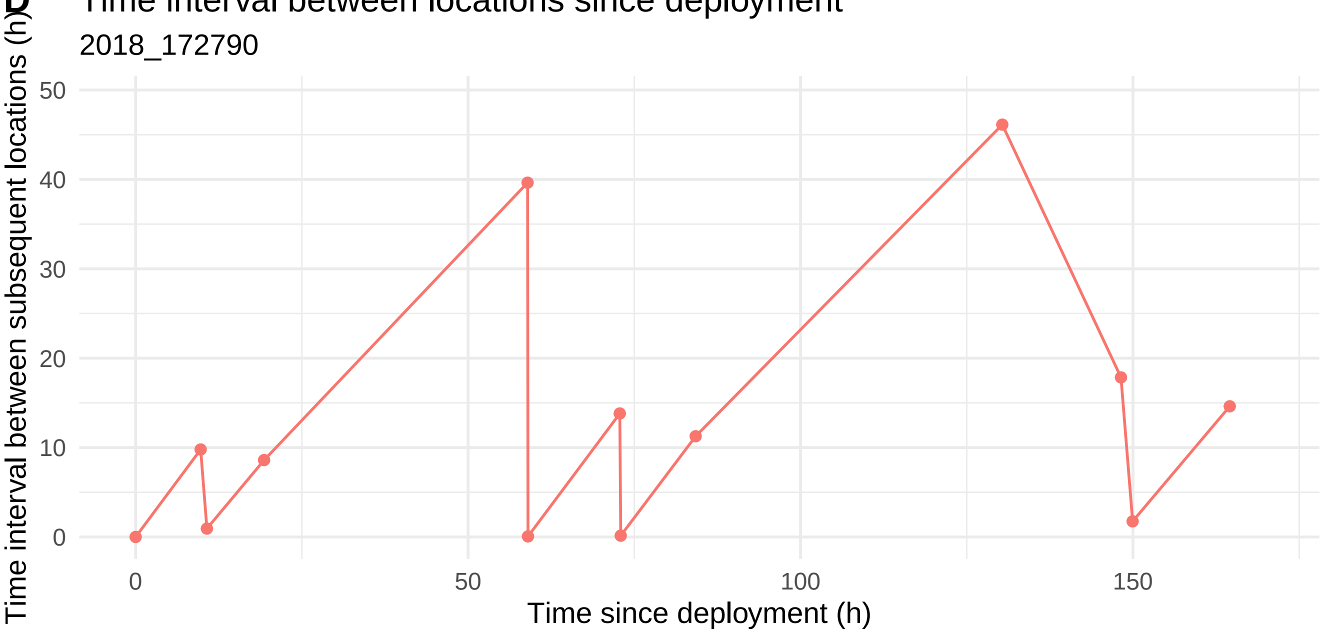

C

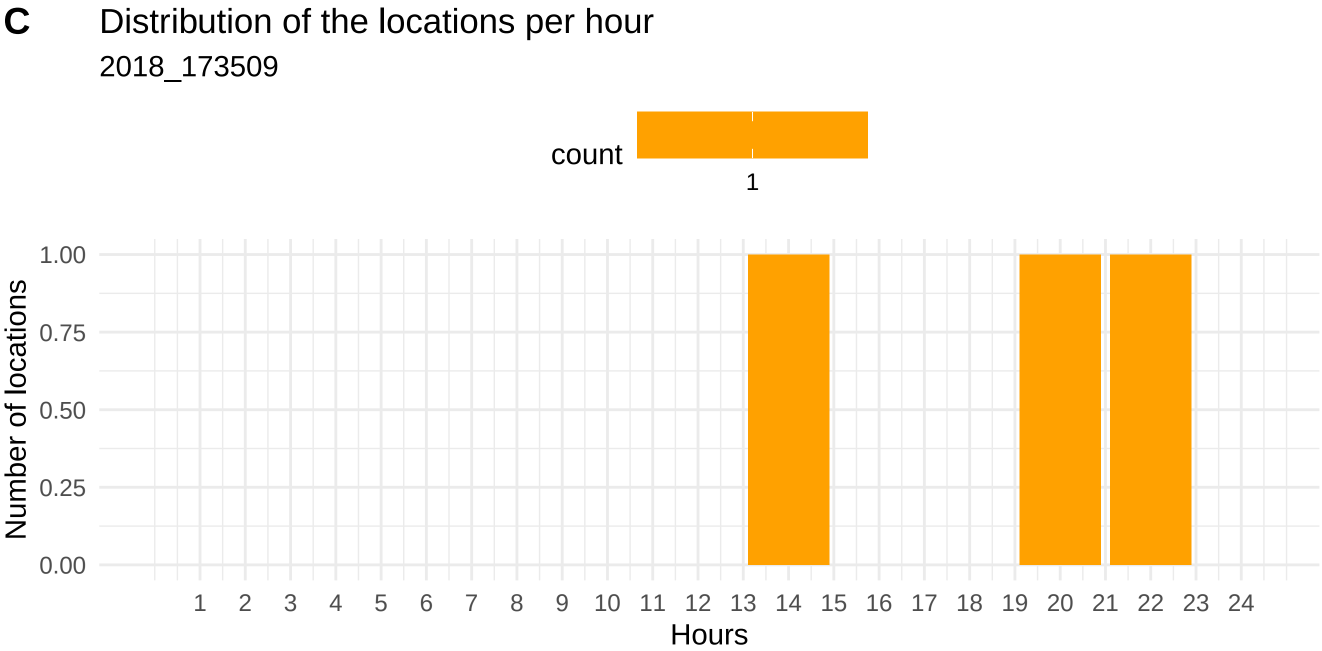
Distribution of the locations per hour
2018_173509
count
1
Number of locations
1.00
0.75
0.50
0.25
0.00
1 2 3 4 5 6 7 8 9 10 11 12 13 14 15 16 17 18 19 20 21 22 23 24
Hours

D

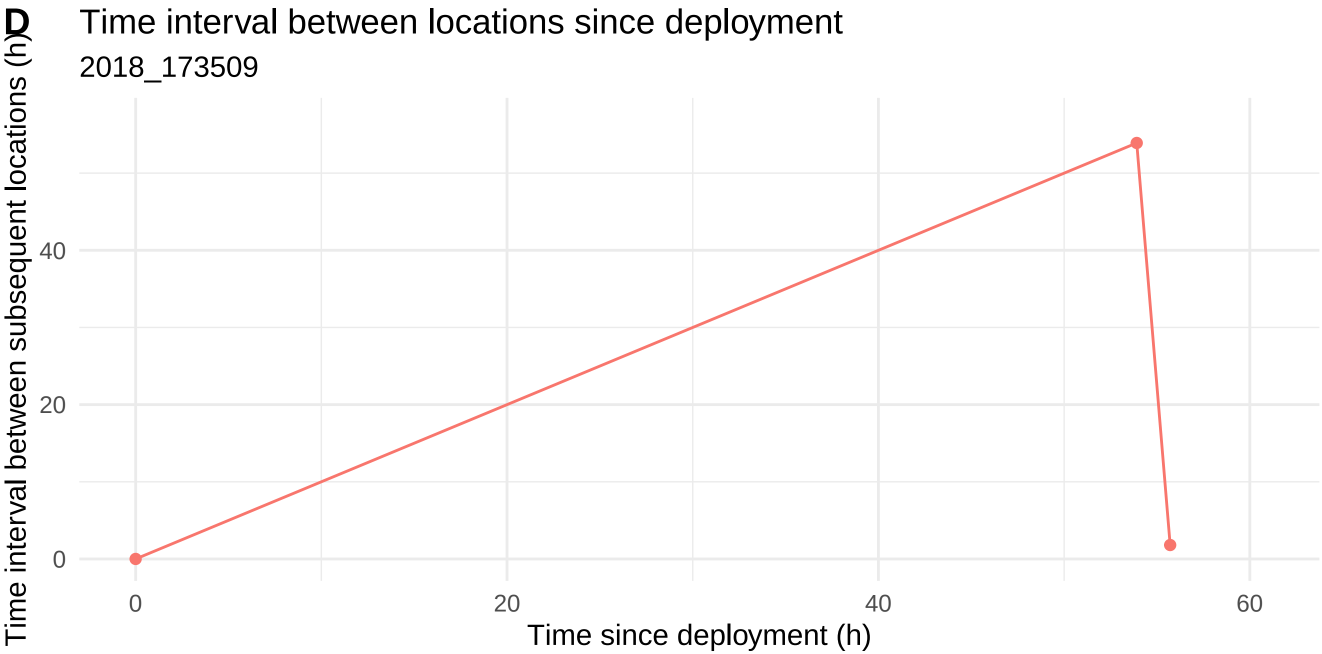
Time interval between locations since deployment
2018_173509
Time interval between subsequent locations (h)
40
20
0
0
20
40
60
Time since deployment (h)

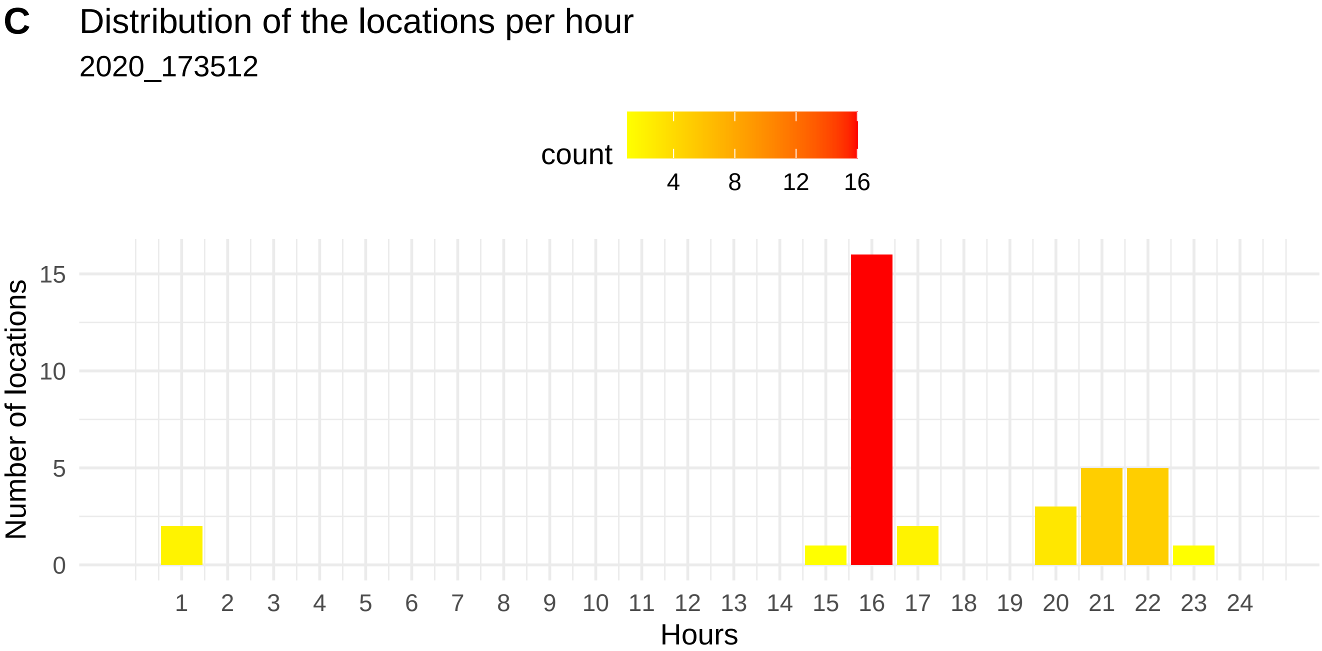
C
Distribution of the locations per hour
2020_173512
count
4
8
12
16
Number of locations
Hours

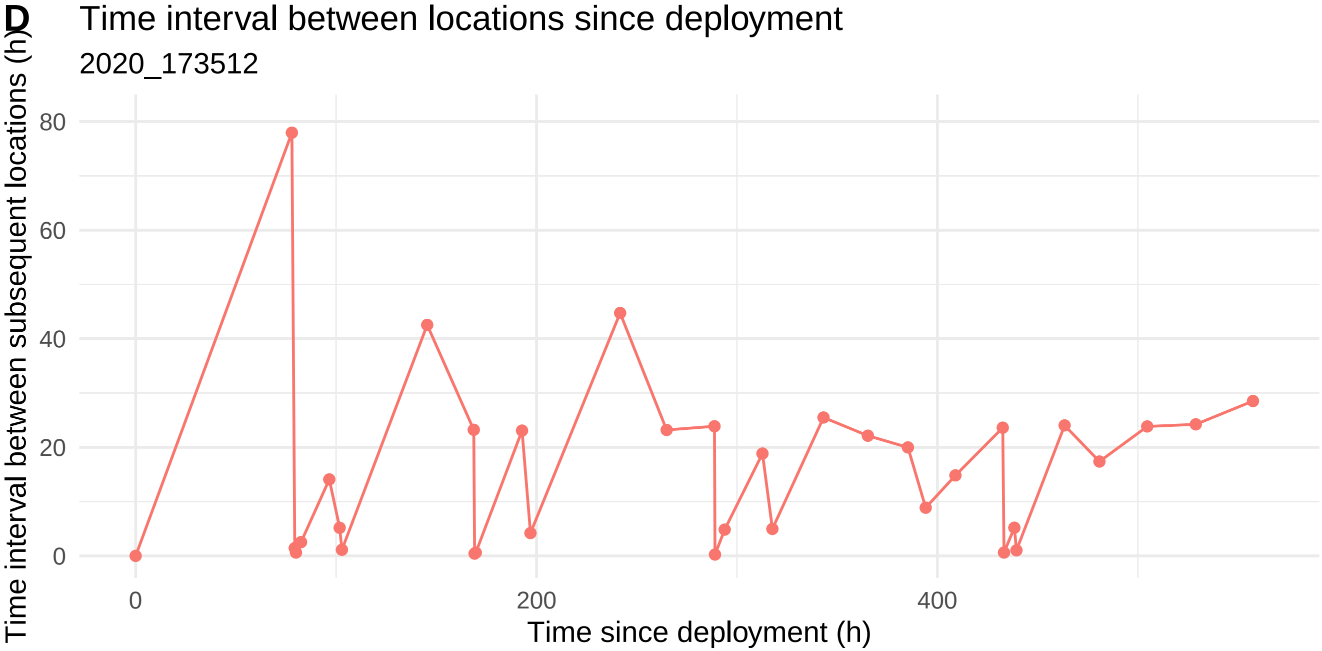
D
Time interval between locations since deployment
2020_173512
Time interval between subsequent locations (h)
Time since deployment (h)

C

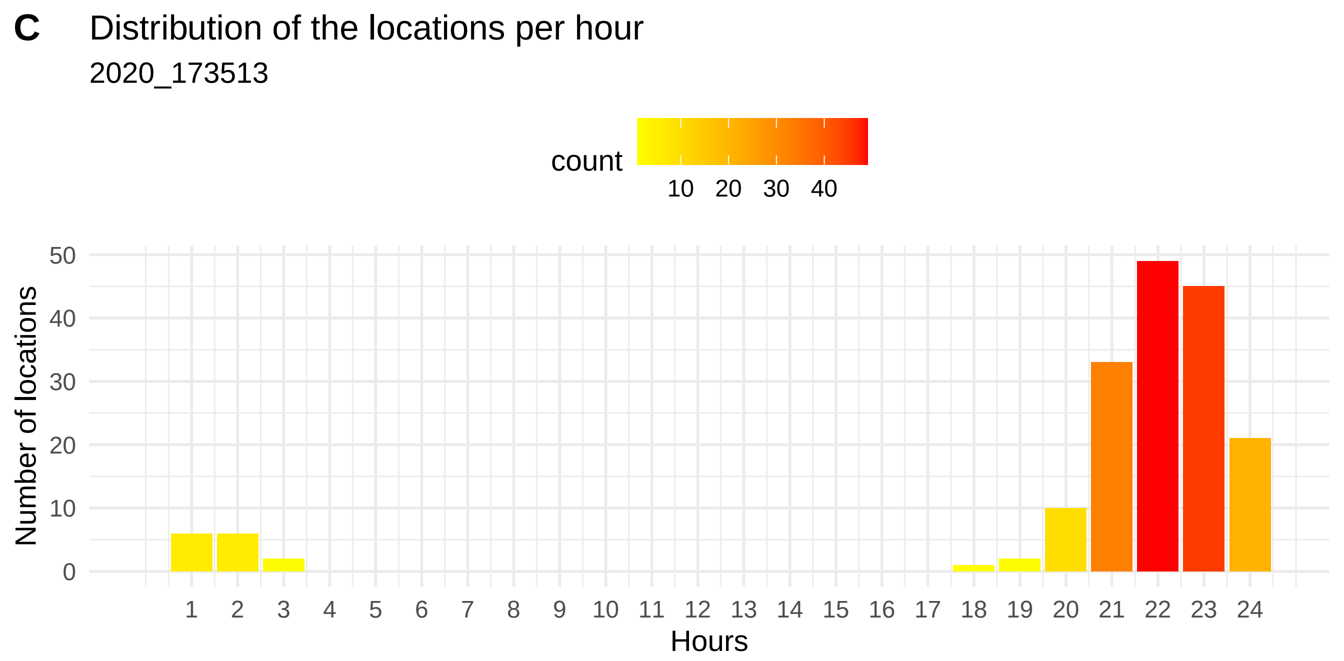

Distribution of the locations per hour
2020_173513
count
10 20 30 40
Number of locations
0 10 20 30 40 50
1 2 3 4 5 6 7 8 9 10 11 12 13 14 15 16 17 18 19 20 21 22 23 24
Hours

D

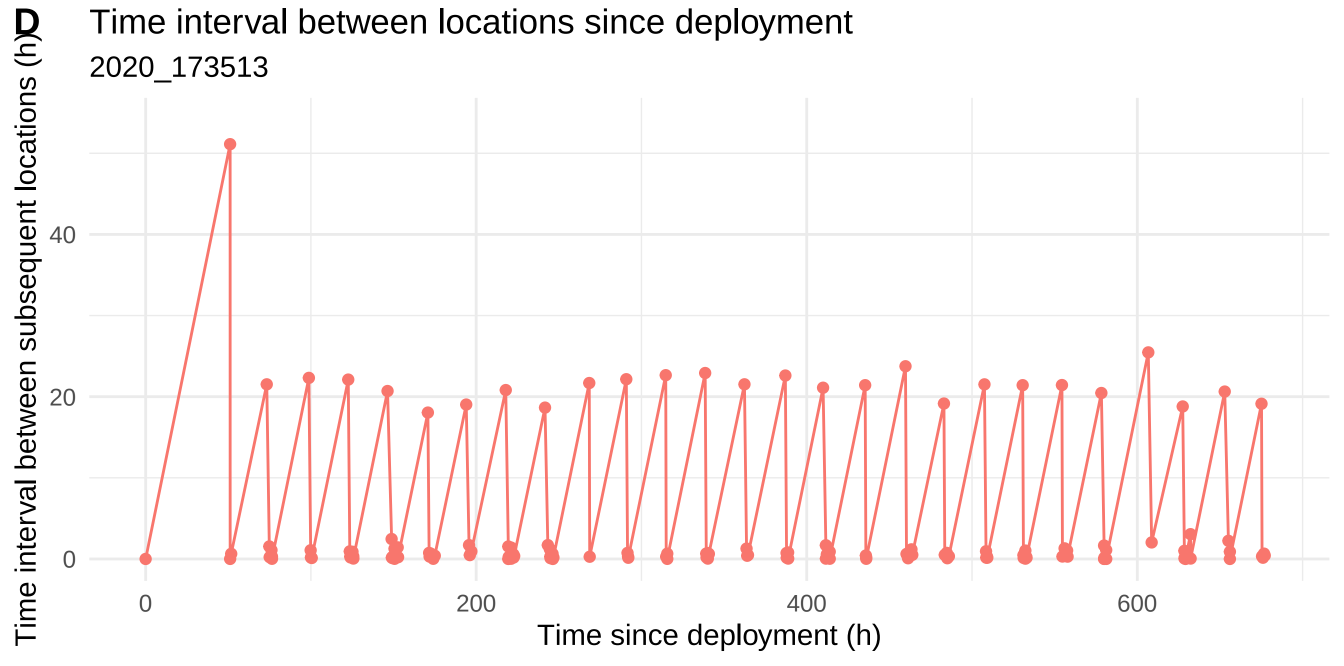

Time interval between locations since deployment
2020_173513
Time interval between subsequent locations (h)
0 20 40
0 200 400 600
Time since deployment (h)

C Distribution of the locations per hour

2020_173514

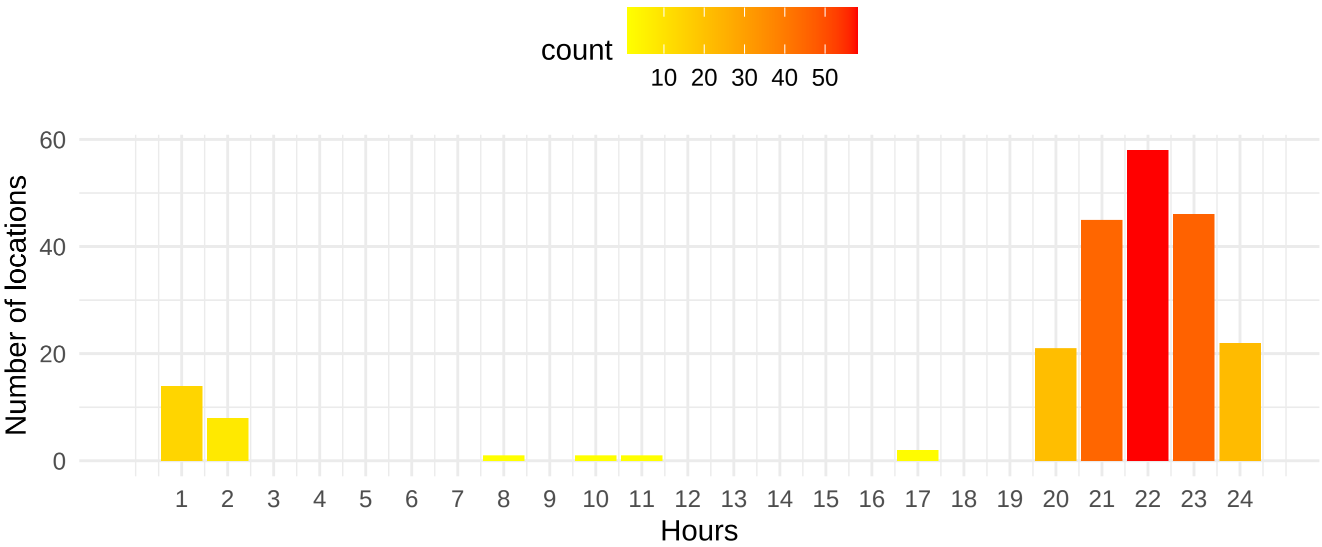

D Time interval between locations since deployment

2020_173514

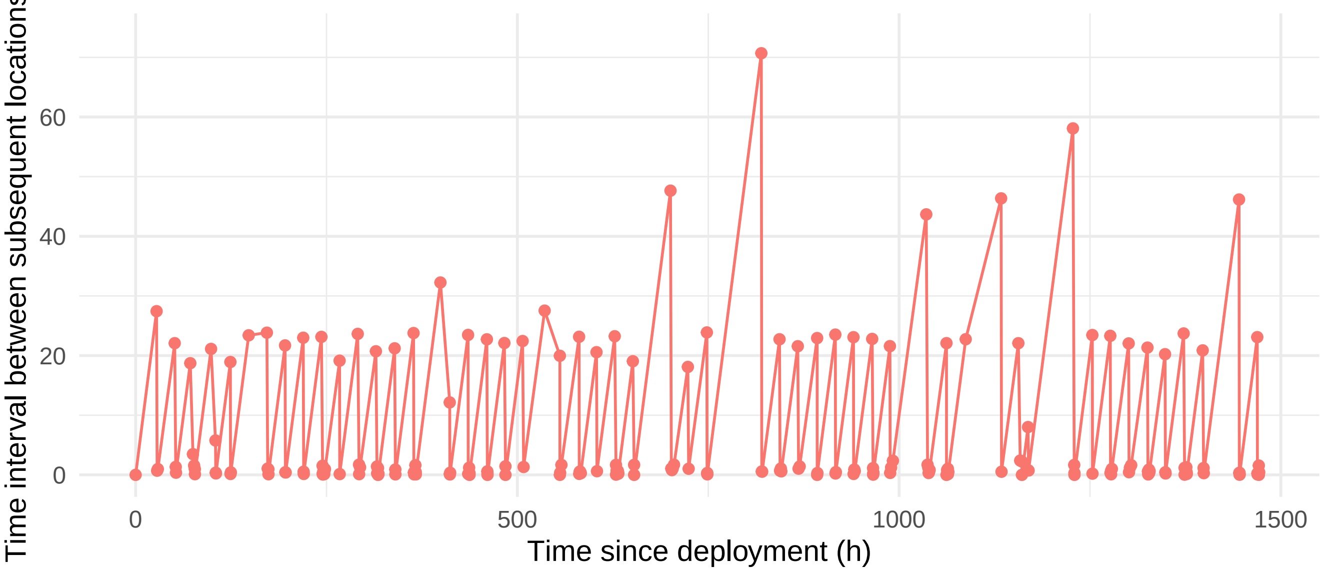

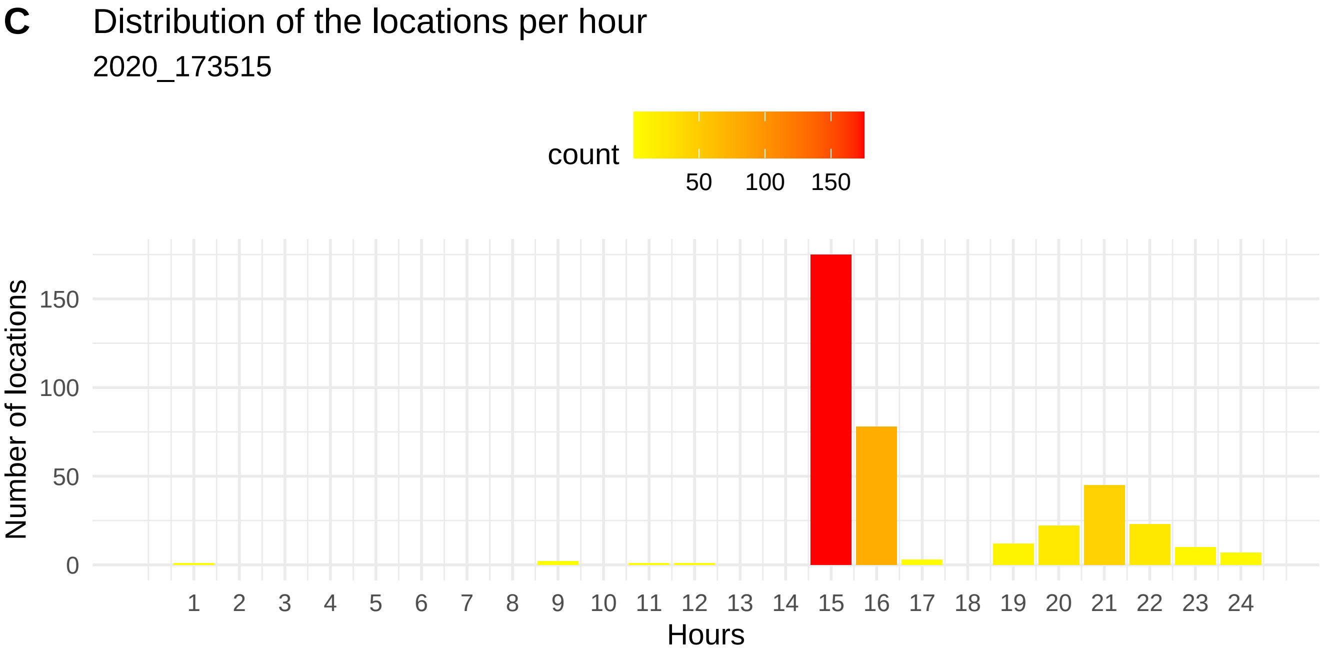
C
Distribution of the locations per hour
2020_173515
count
50
100
150
Number of locations
0
50
100
150
1
2
3
4
5
6
7
8
9
10
11
12
13
14
15
16
17
18
19
20
21
22
23
24
Hours

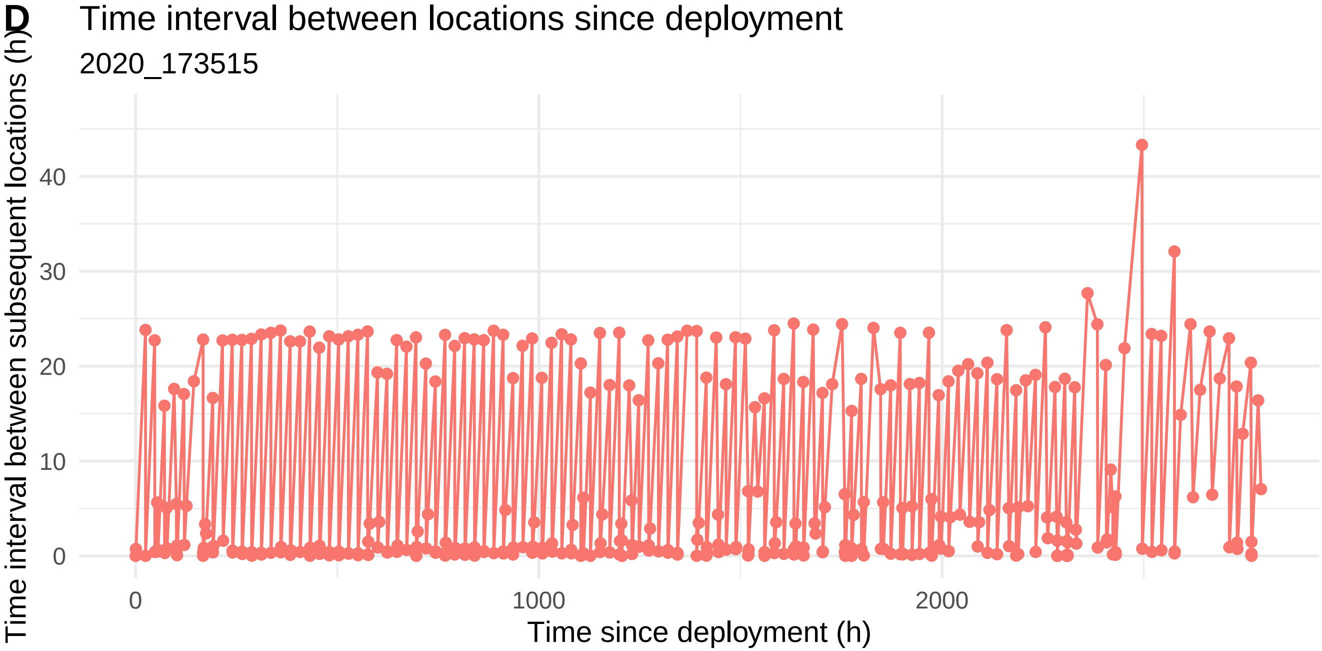
D
Time interval between locations since deployment
2020_173515
Time interval between subsequent locations (h)
0
10
20
30
40
0
1000
2000
Time since deployment (h)

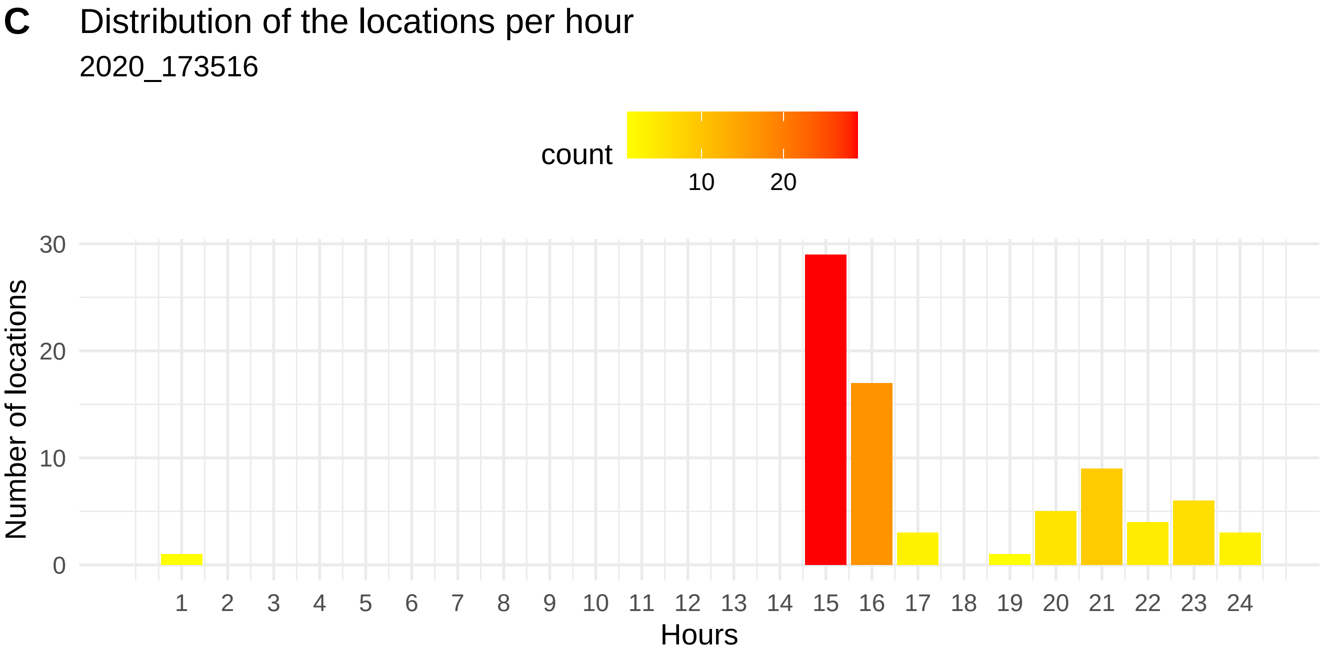
C
Distribution of the locations per hour
2020_173516
count
10
20
30
20
10
0
Number of locations
1 2 3 4 5 6 7 8 9 10 11 12 13 14 15 16 17 18 19 20 21 22 23 24
Hours

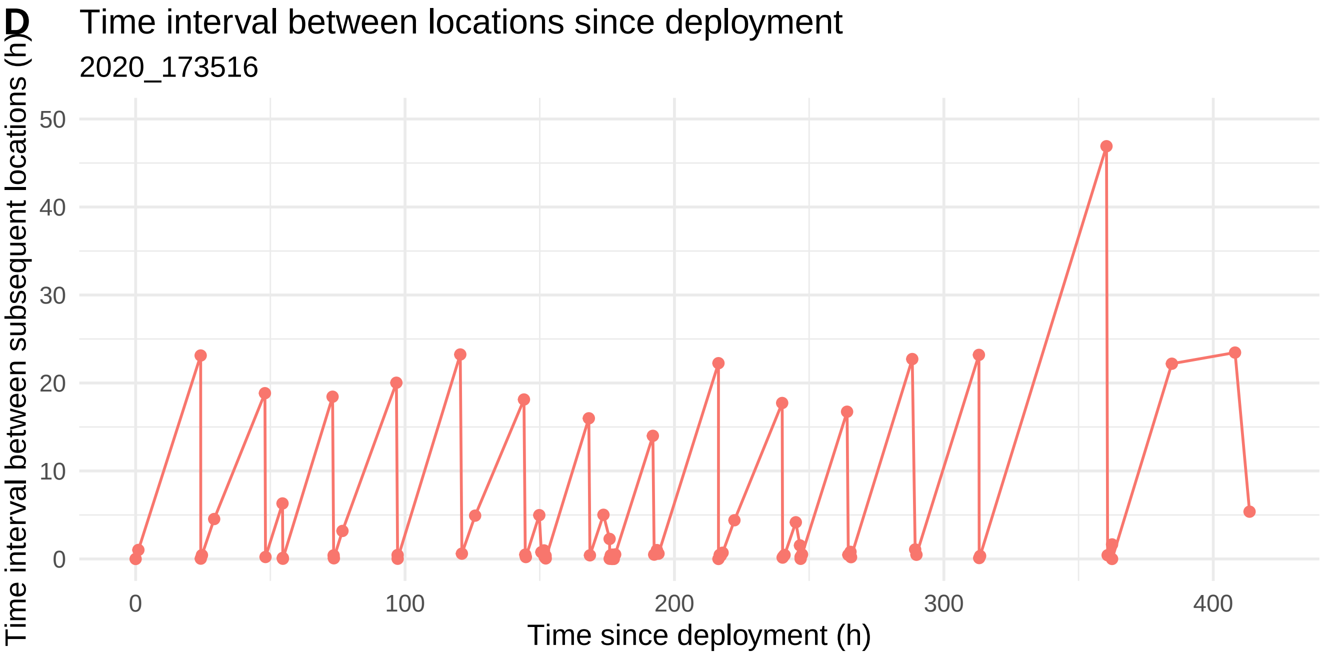
D
Time interval between locations since deployment
2020_173516
50
40
30
20
10
0
Time interval between subsequent locations (h)
0
100
200
300
400
Time since deployment (h)

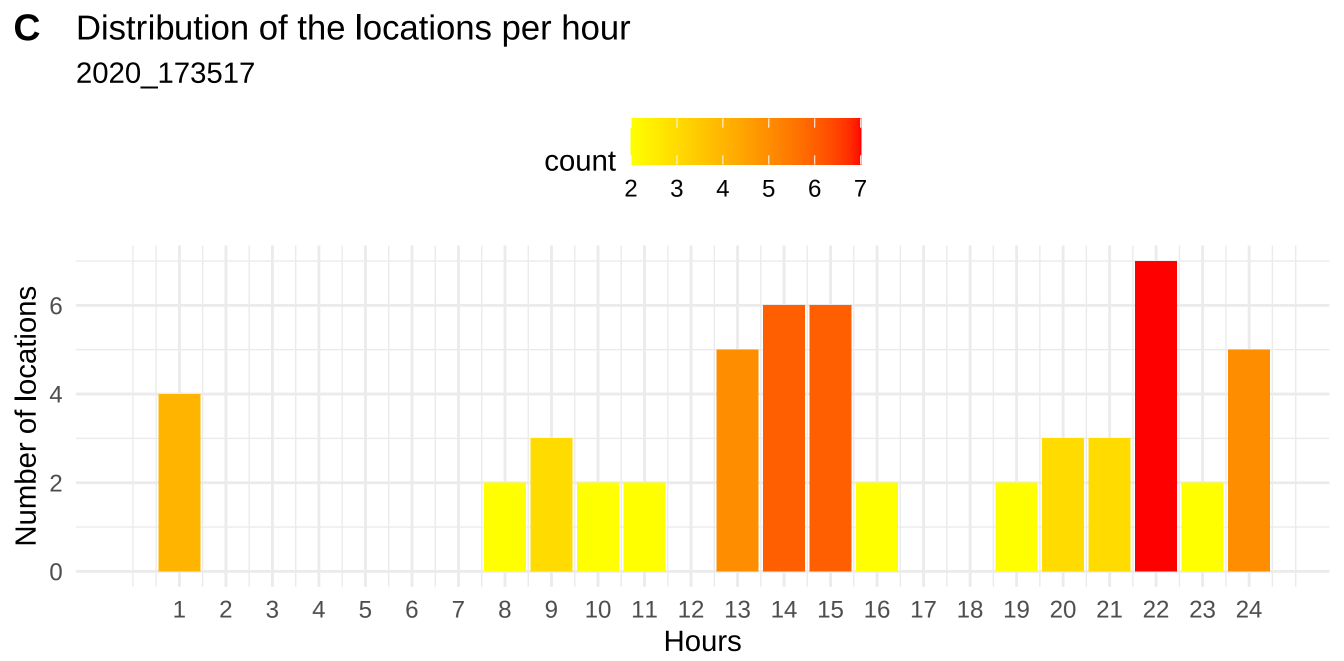
C
Distribution of the locations per hour
2020_173517
count
2 3 4 5 6 7
Number of locations
0 2 4 6
1 2 3 4 5 6 7 8 9 10 11 12 13 14 15 16 17 18 19 20 21 22 23 24
Hours

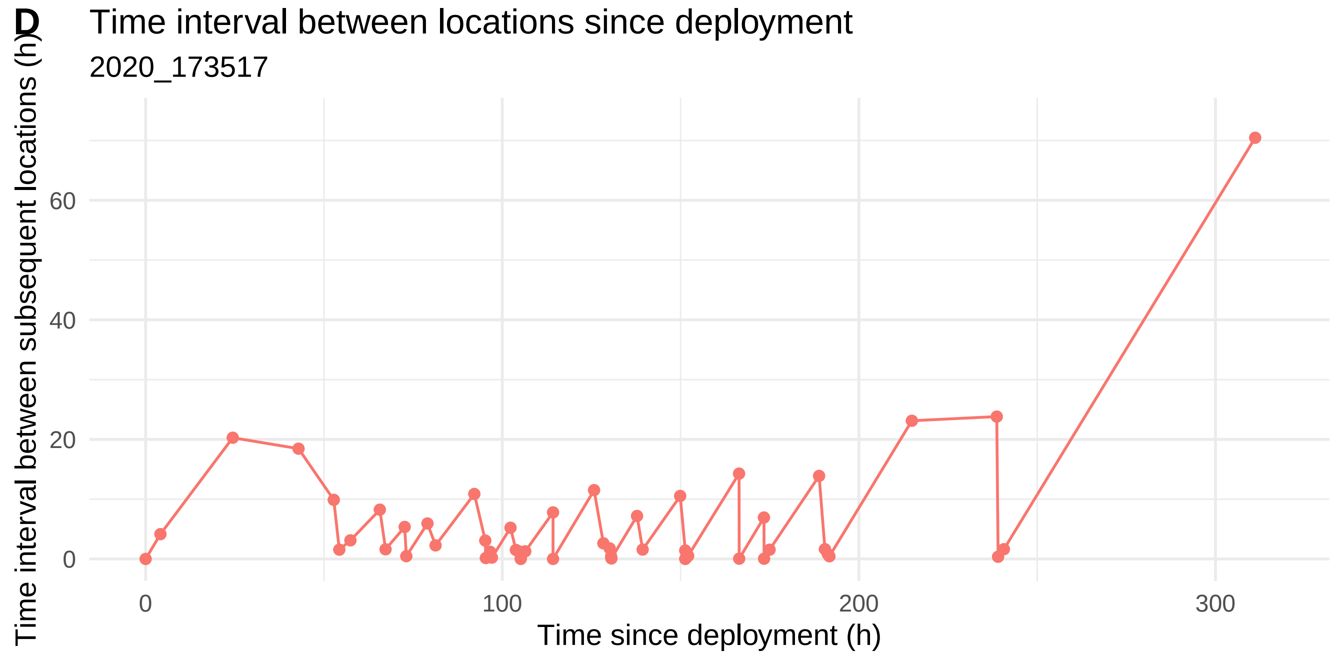
D
Time interval between locations since deployment
2020_173517
Time interval between subsequent locations (h)
0 20 40 60
0 100 200 300
Time since deployment (h)

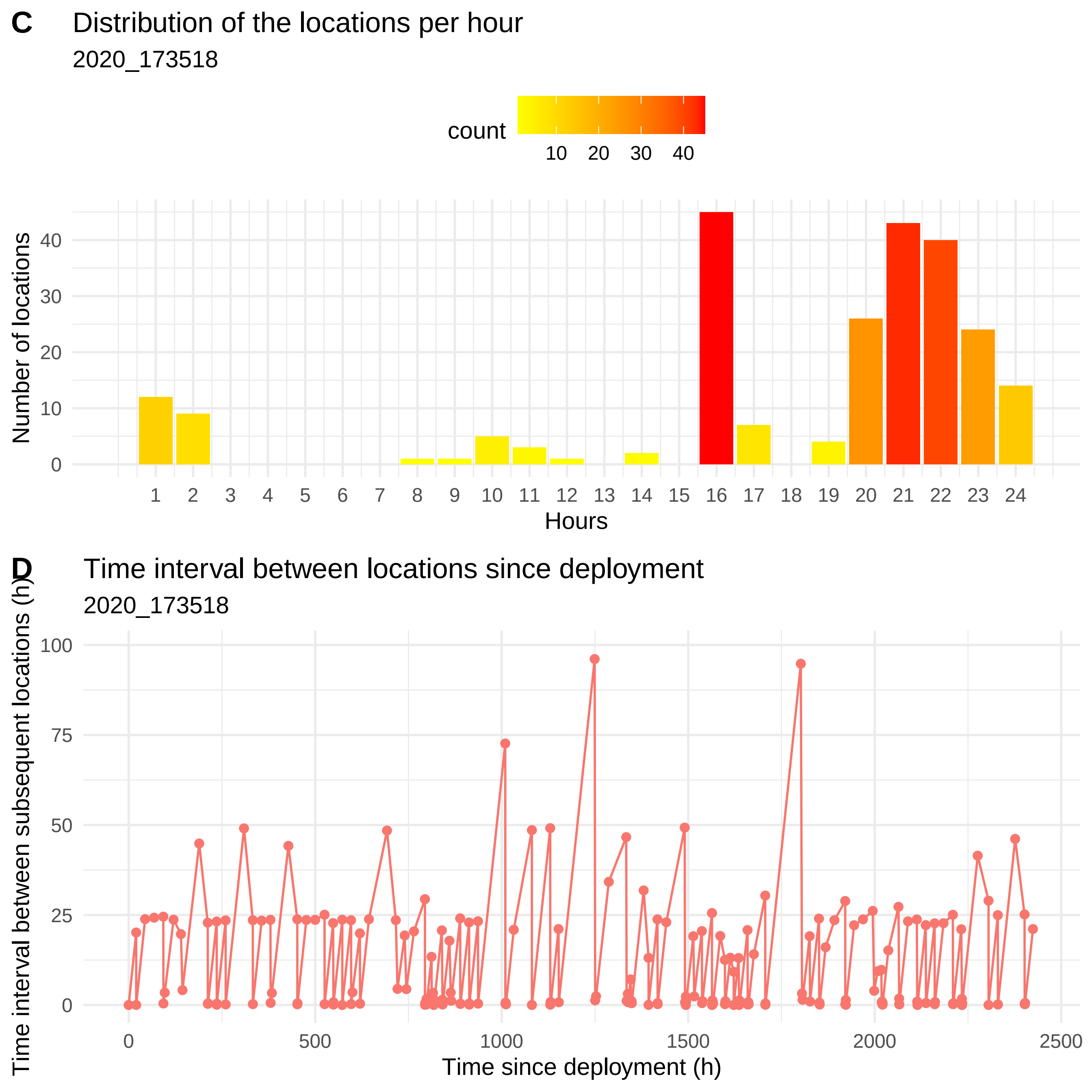
C
Distribution of the locations per hour
2020_173518
count
10 20 30 40
Number of locations
0 10 20 30 40
1 2 3 4 5 6 7 8 9 10 11 12 13 14 15 16 17 18 19 20 21 22 23 24
Hours
D
Time interval between locations since deployment
2020_173518
Time interval between subsequent locations (h)
0 25 50 75 100
0 500 1000 1500 2000 2500
Time since deployment (h)

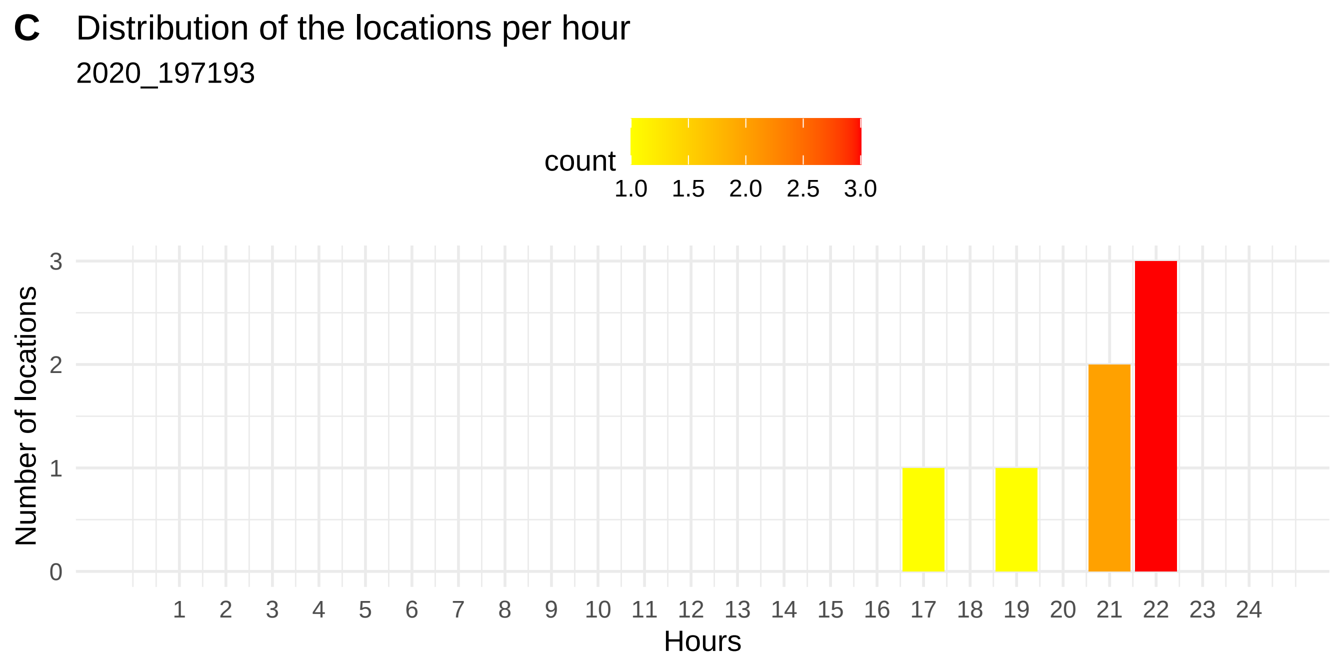
C
Distribution of the locations per hour
2020_197193
count
1.0
1.5
2.0
2.5
3.0
Number of locations
0
1
2
3
1 2 3 4 5 6 7 8 9 10 11 12 13 14 15 16 17 18 19 20 21 22 23 24
Hours

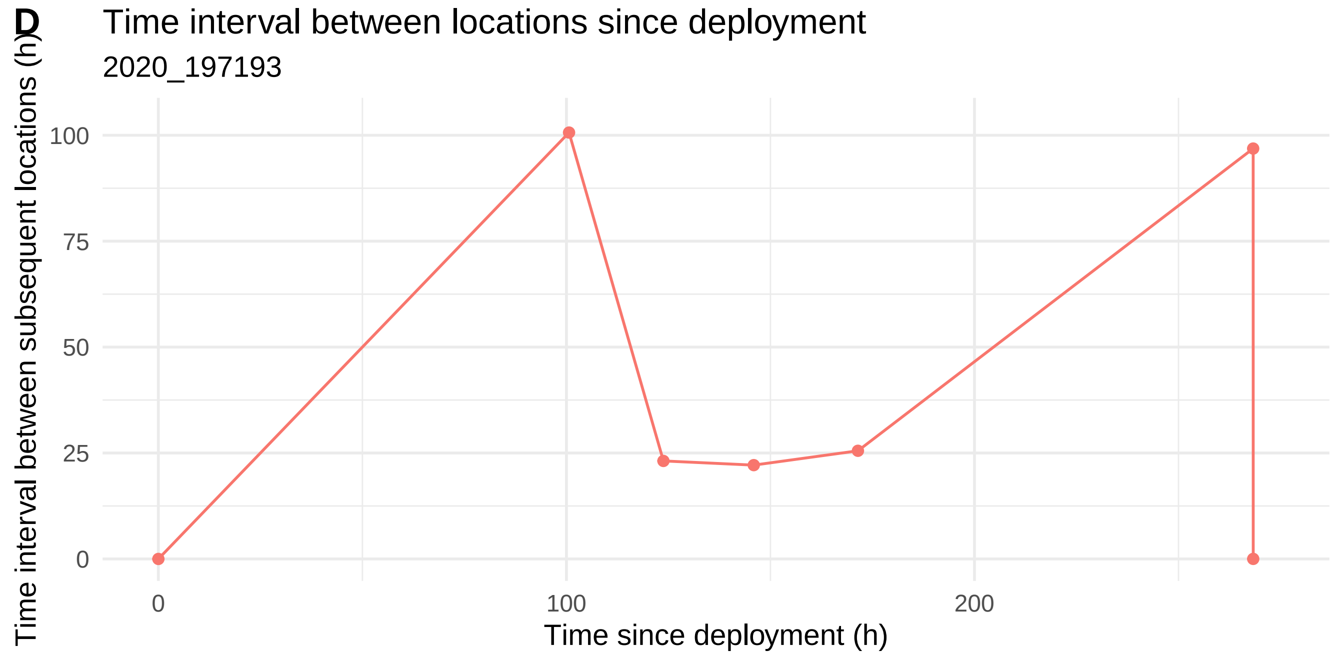
D
Time interval between locations since deployment
2020_197193
Time interval between subsequent locations (h)
0
25
50
75
100
0
100
200
Time since deployment (h)

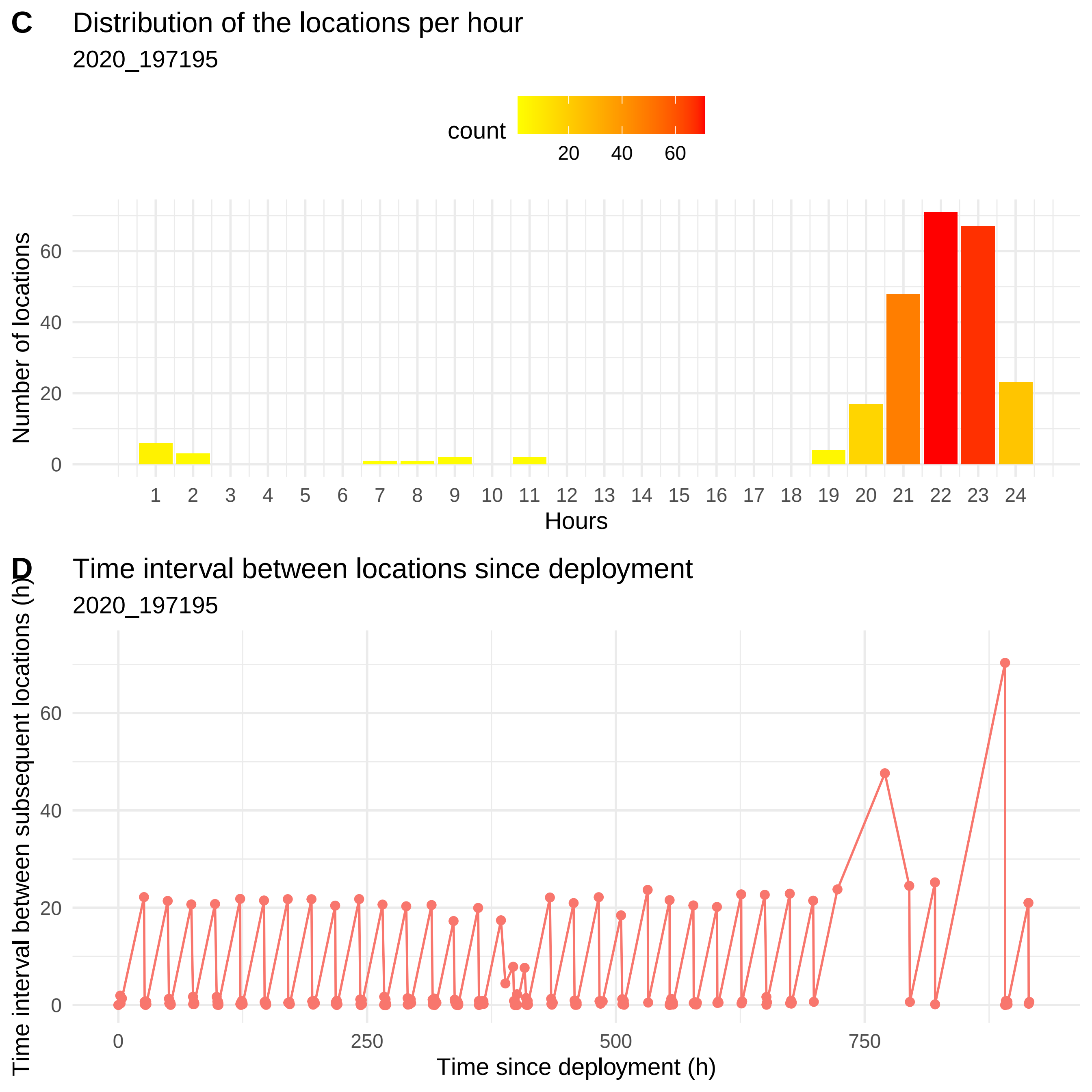
C
Distribution of the locations per hour
2020_197195
count
20
40
60
Number of locations
0
20
40
60
1 2 3 4 5 6 7 8 9 10 11 12 13 14 15 16 17 18 19 20 21 22 23 24
Hours
D
Time interval between locations since deployment
2020_197195
Time interval between subsequent locations (h)
0
20
40
60
0
250
500
750
Time since deployment (h)

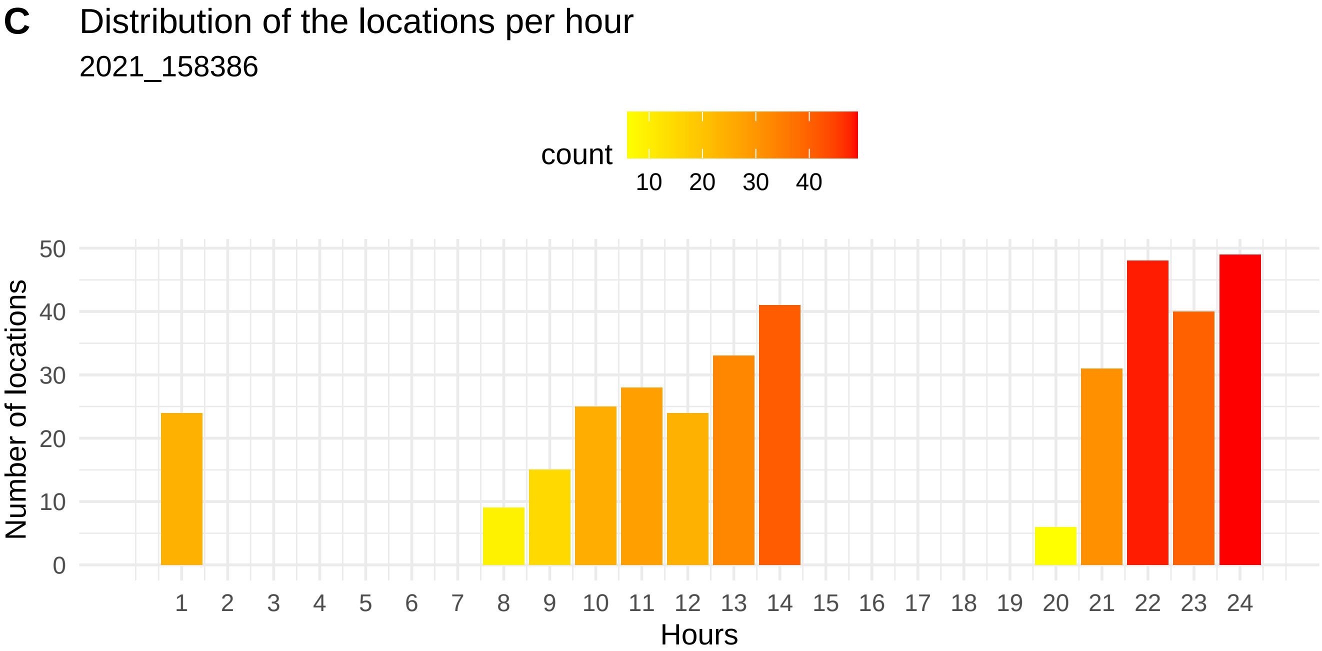
C
Distribution of the locations per hour
2021_158386
count
10
20
30
40
Number of locations
Hours
0
10
20
30
40
50
1
2
3
4
5
6
7
8
9
10
11
12
13
14
15
16
17
18
19
20
21
22
23
24

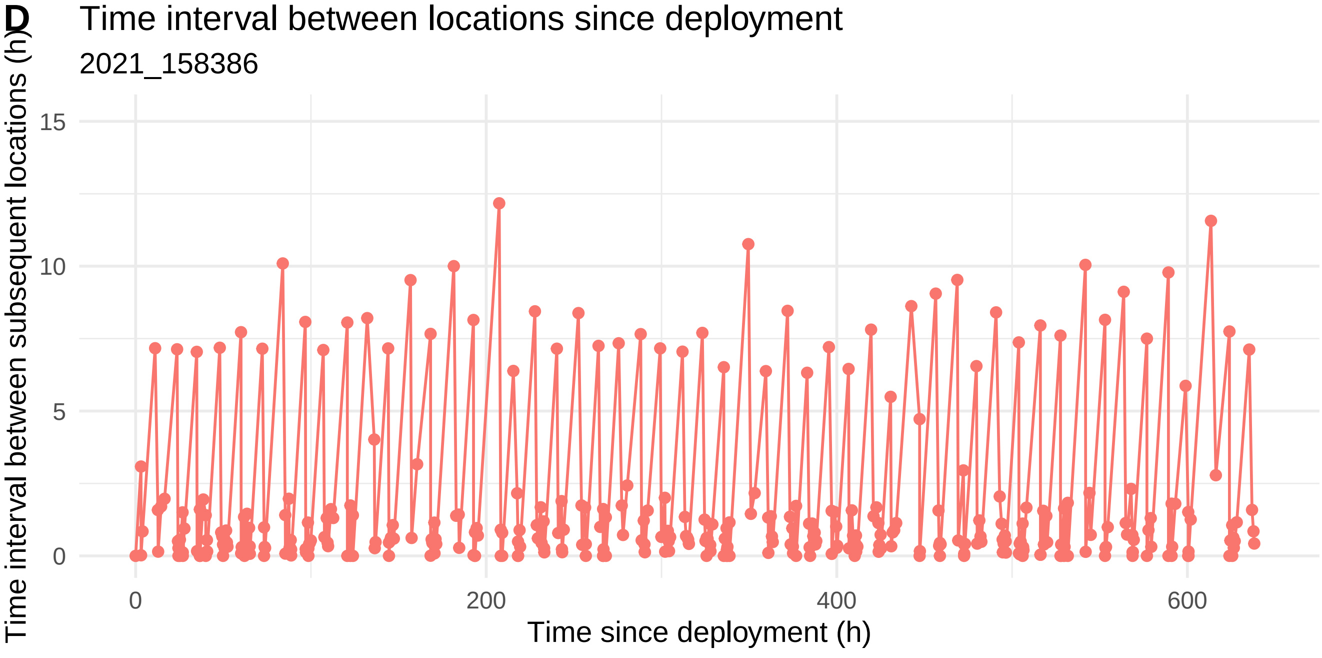
D
Time interval between locations since deployment
2021_158386
Time interval between subsequent locations (h)
Time since deployment (h)
0
5
10
15
0
200
400
600

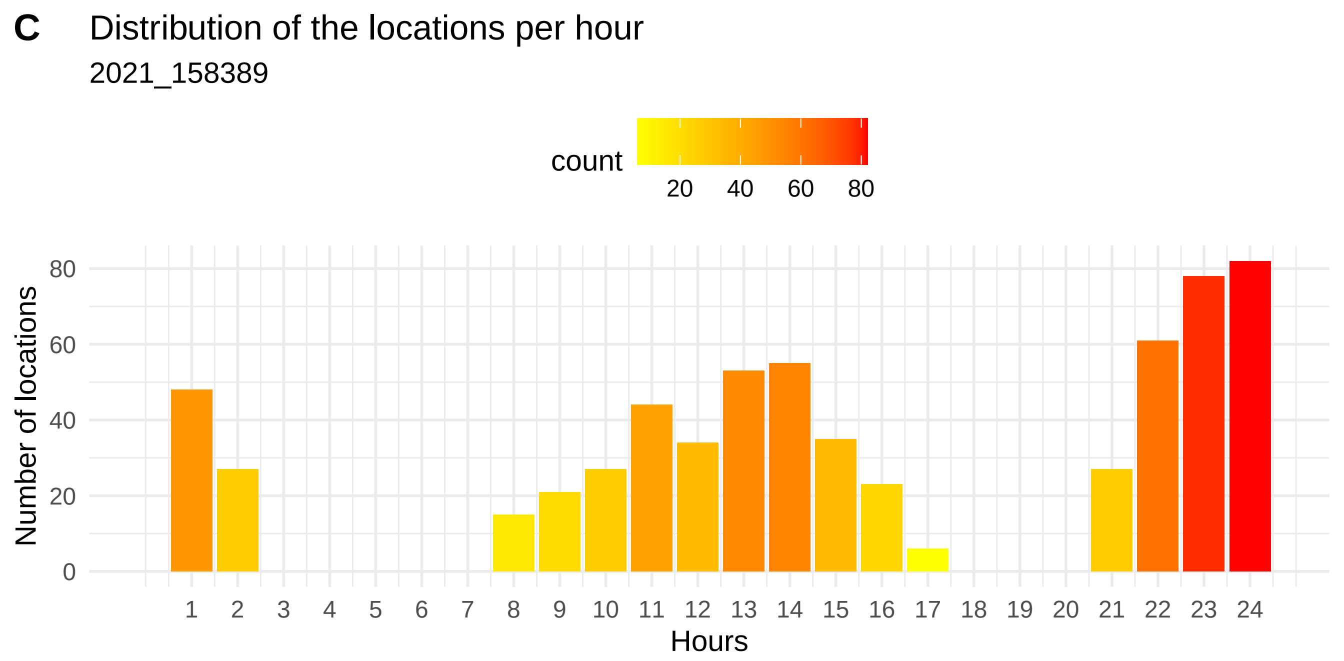
C
Distribution of the locations per hour
2021_158389
count
20
40
60
80
Number of locations
0
20
40
60
80
1 2 3 4 5 6 7 8 9 10 11 12 13 14 15 16 17 18 19 20 21 22 23 24
Hours

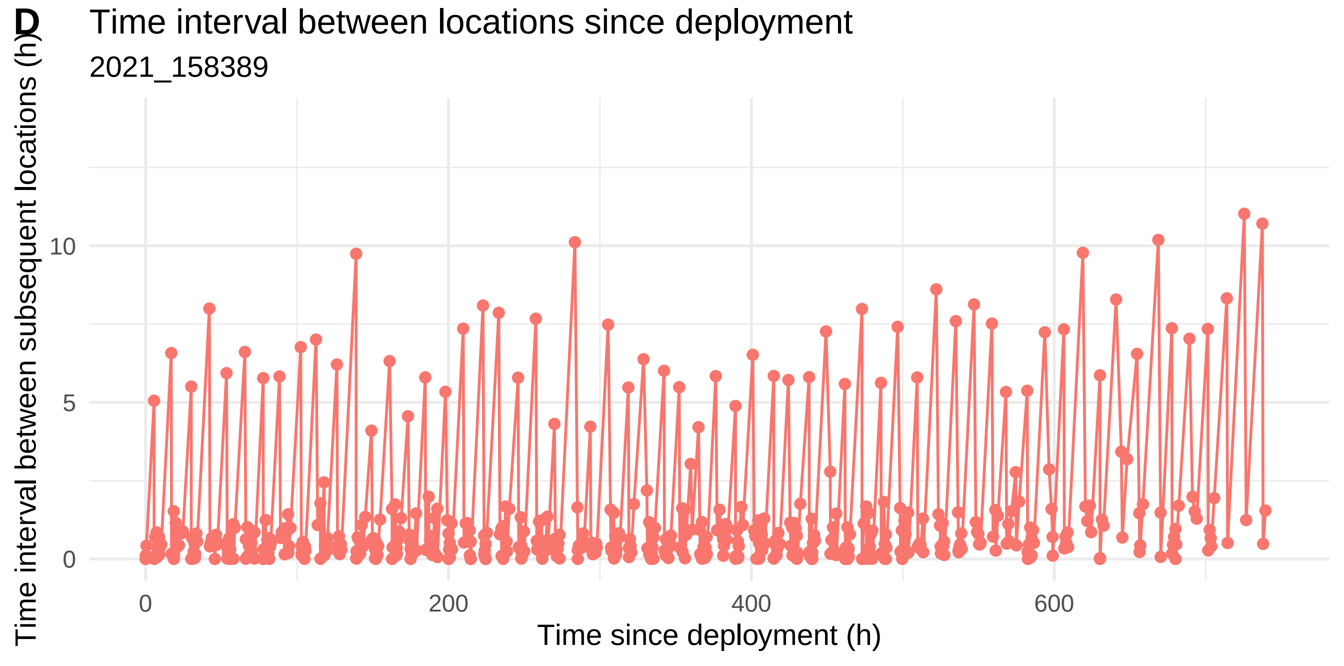
D
Time interval between locations since deployment
2021_158389
Time interval between subsequent locations (h)
0
5
10
0
200
400
600
Time since deployment (h)

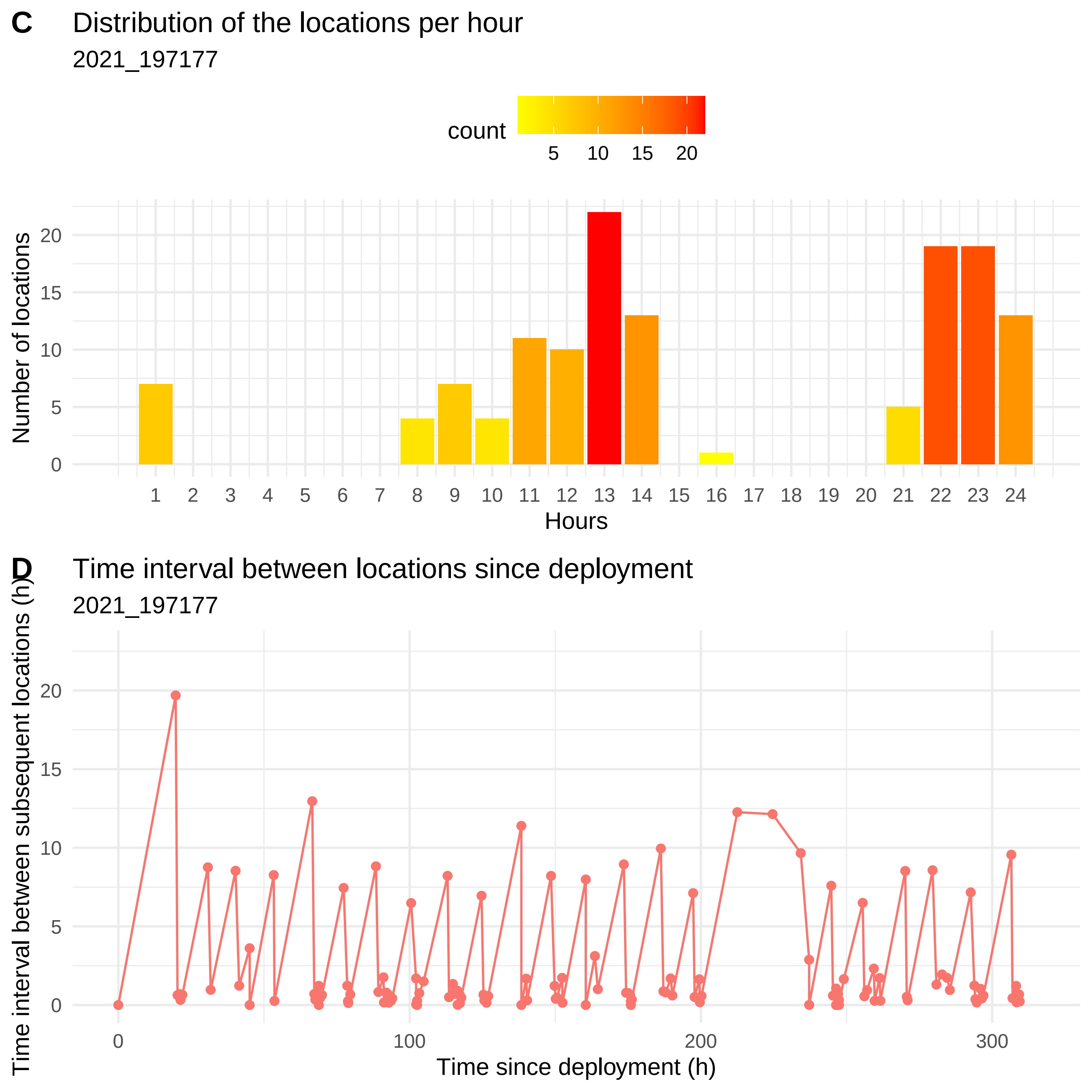
C
Distribution of the locations per hour
2021_197177
count
5
10
15
20
Number of locations
0
5
10
15
20
1 2 3 4 5 6 7 8 9 10 11 12 13 14 15 16 17 18 19 20 21 22 23 24
Hours
D
Time interval between locations since deployment
2021_197177
Time interval between subsequent locations (h)
0
5
10
15
20
0
100
200
300
Time since deployment (h)

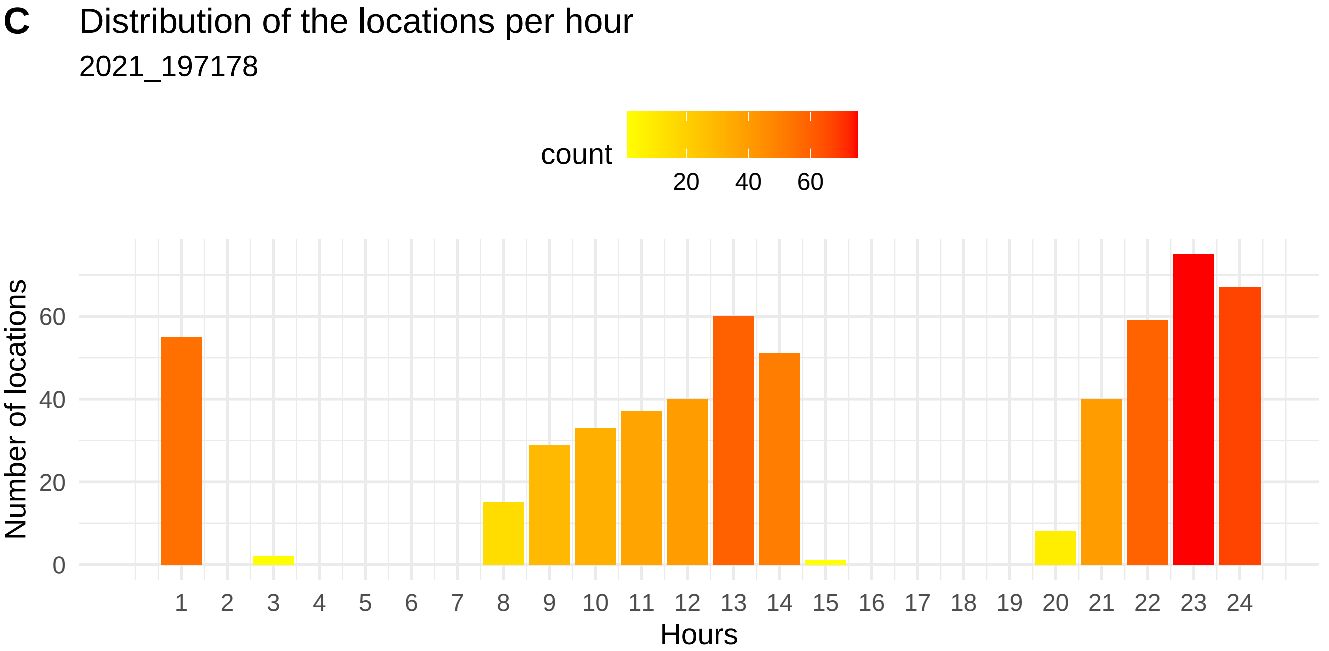
C
Distribution of the locations per hour
2021_197178
count
20
40
60
Number of locations
0
20
40
60
1
2
3
4
5
6
7
8
9
10
11
12
13
14
15
16
17
18
19
20
21
22
23
24
Hours

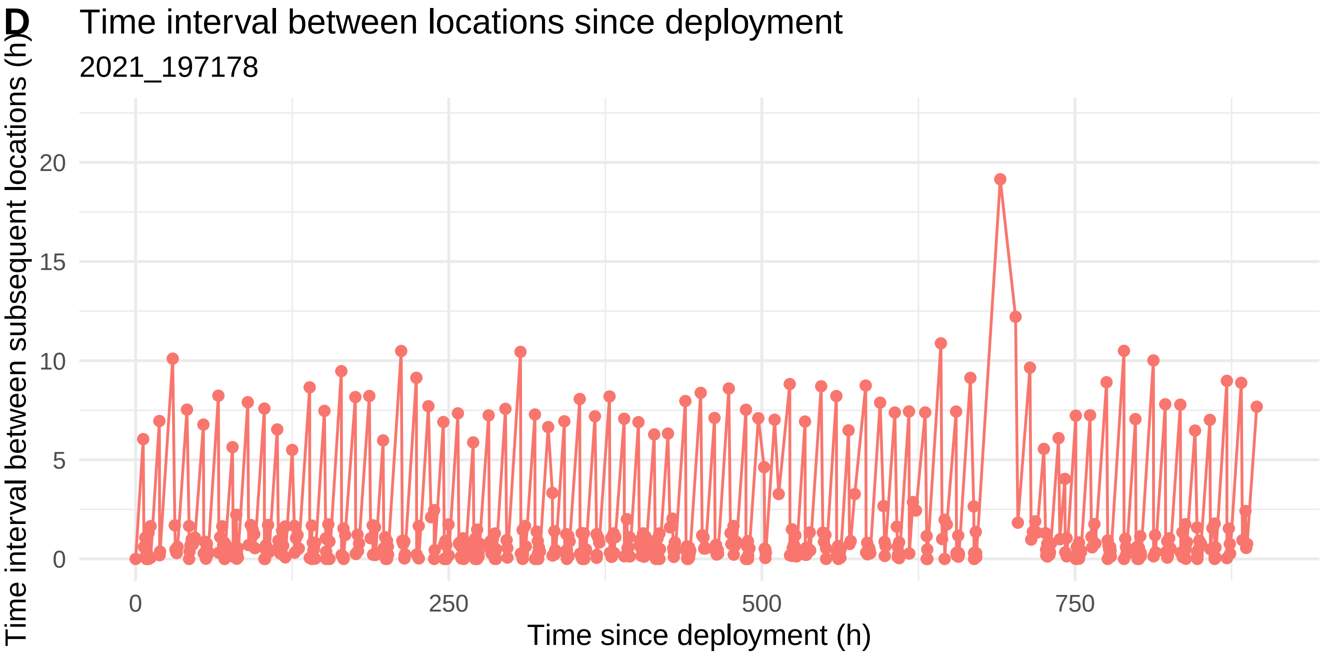
D
Time interval between locations since deployment
2021_197178
Time interval between subsequent locations (h)
0
5
10
15
20
0
250
500
750
Time since deployment (h)

C Distribution of the locations per hour
2021_197179

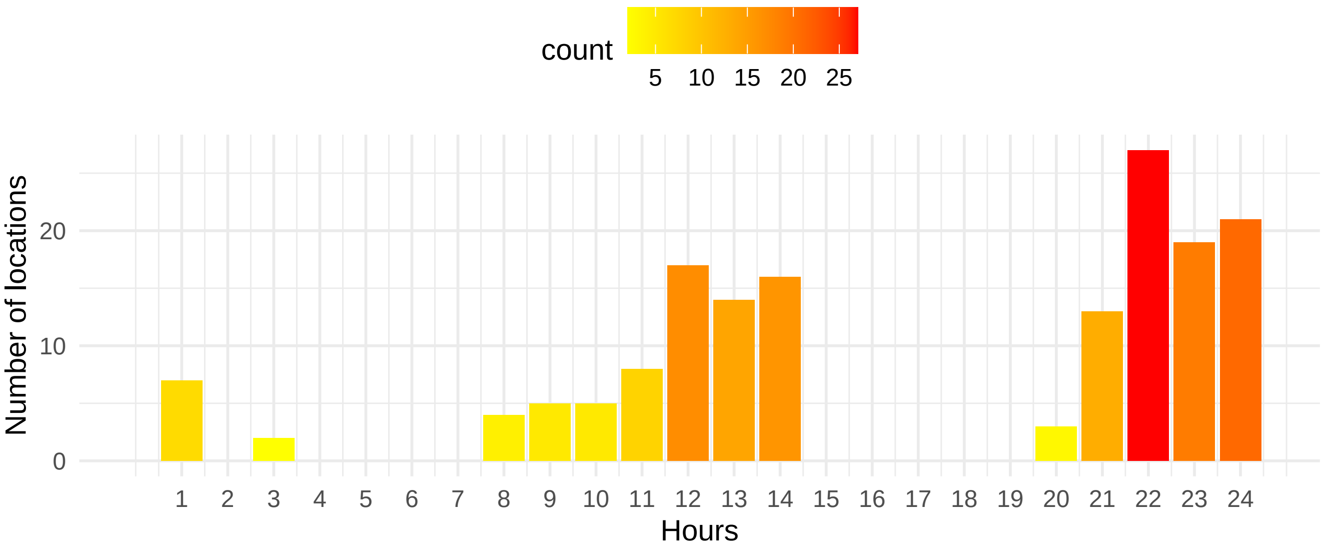

D Time interval between locations since deployment
2021_197179

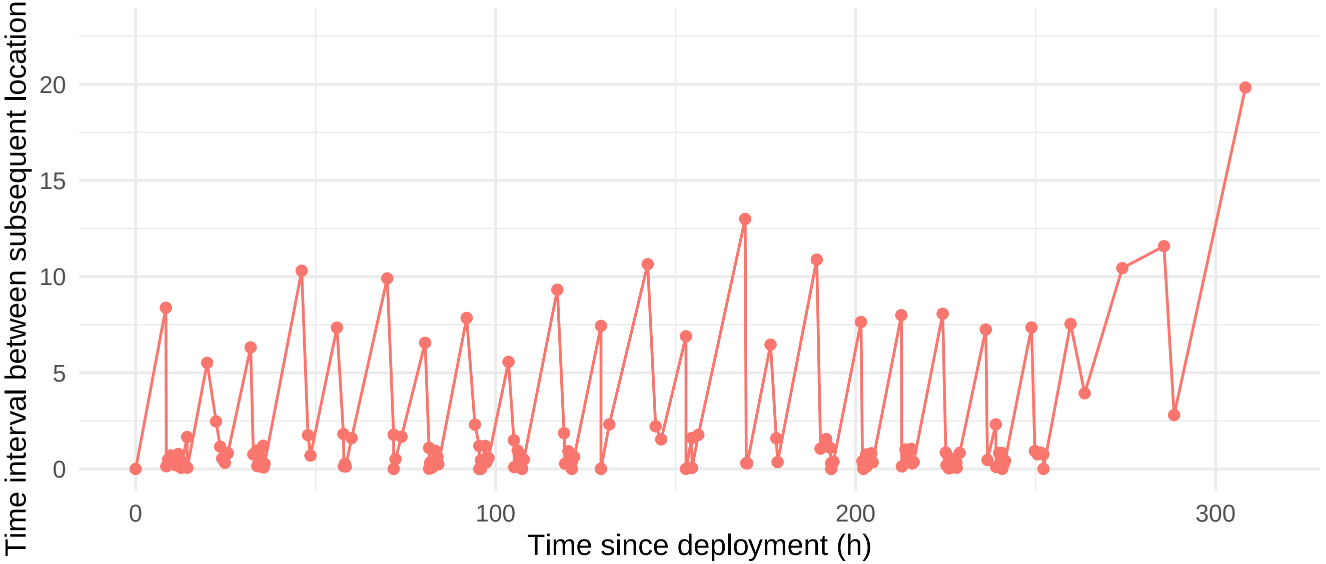

C Distribution of the locations per hour

2021_197181

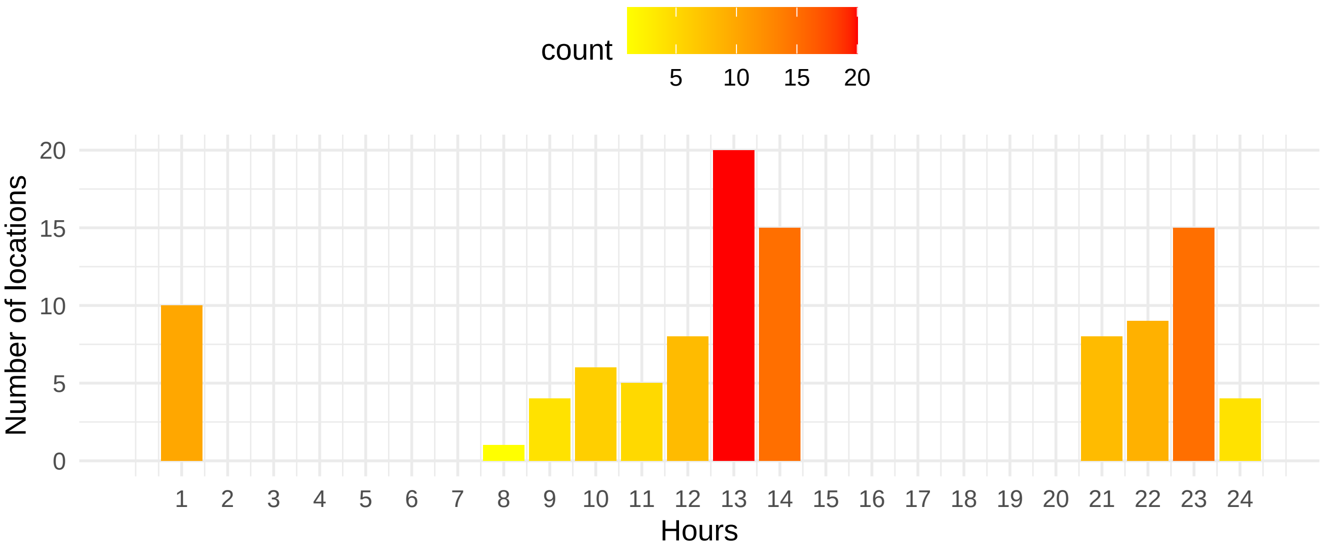

D Time interval between locations since deployment

2021_197181

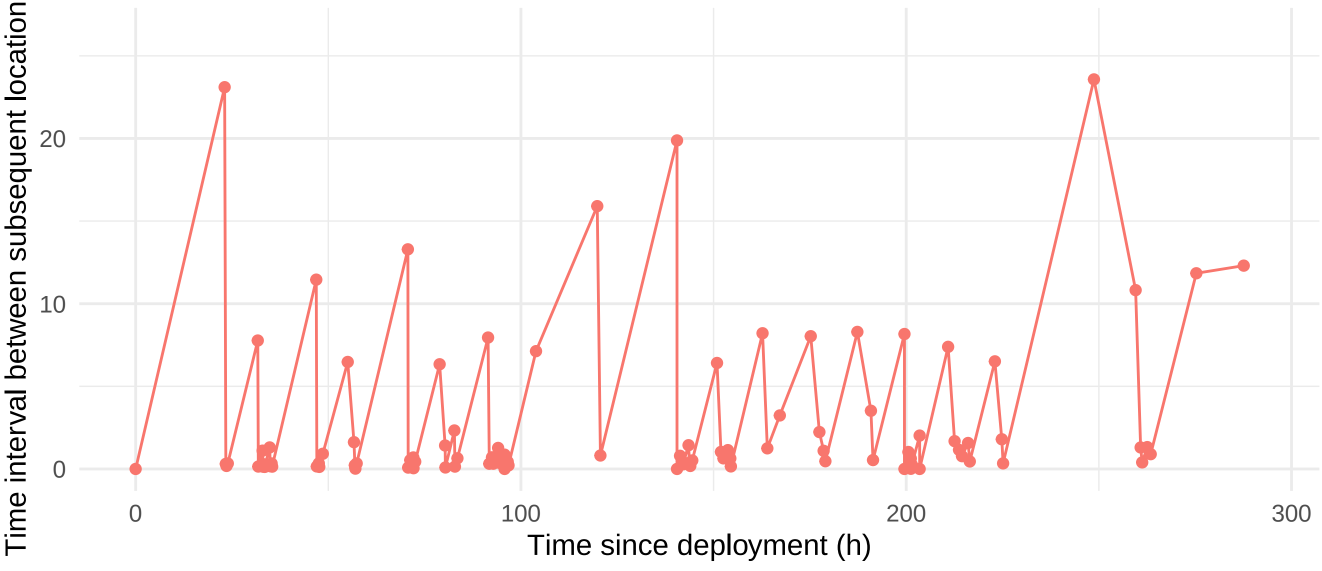

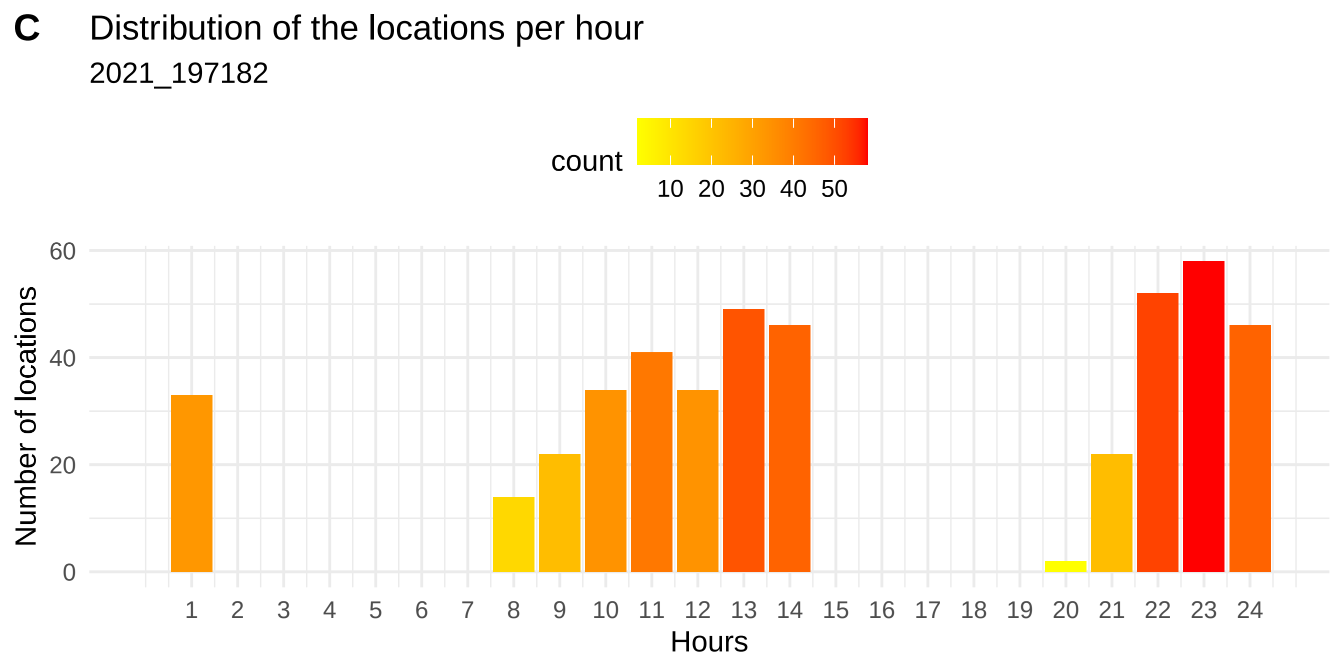
C
Distribution of the locations per hour
2021_197182
count
10 20 30 40 50
Number of locations
0
20
40
60
1 2 3 4 5 6 7 8 9 10 11 12 13 14 15 16 17 18 19 20 21 22 23 24
Hours

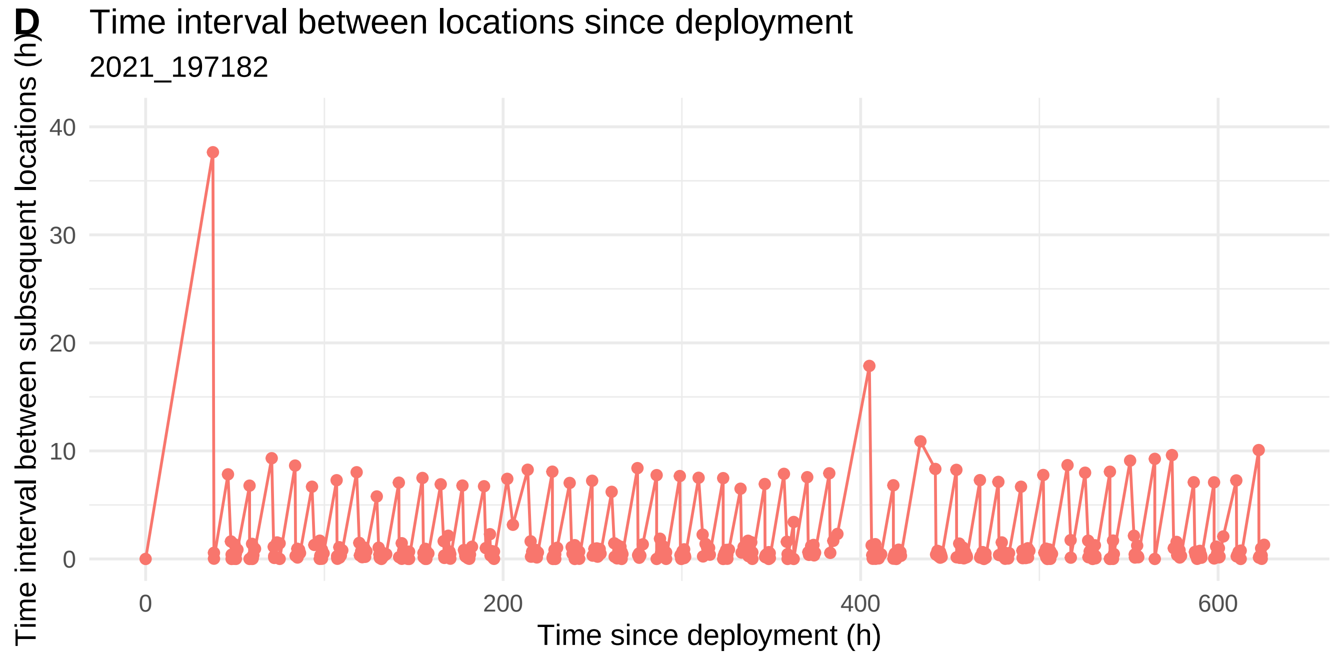
D
Time interval between locations since deployment
2021_197182
Time interval between subsequent locations (h)
0
10
20
30
40
0
200
400
600
Time since deployment (h)

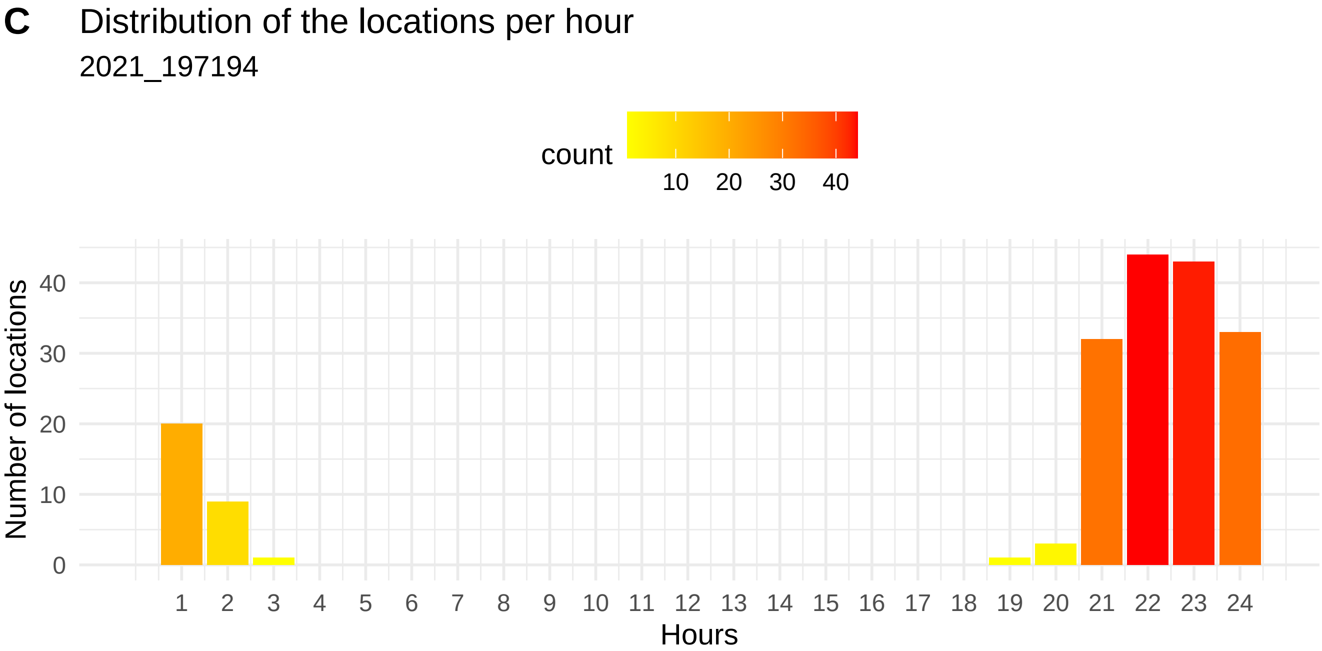
C
Distribution of the locations per hour
2021_197194
count
10 20 30 40
Number of locations
0 10 20 30 40
1 2 3 4 5 6 7 8 9 10 11 12 13 14 15 16 17 18 19 20 21 22 23 24
Hours

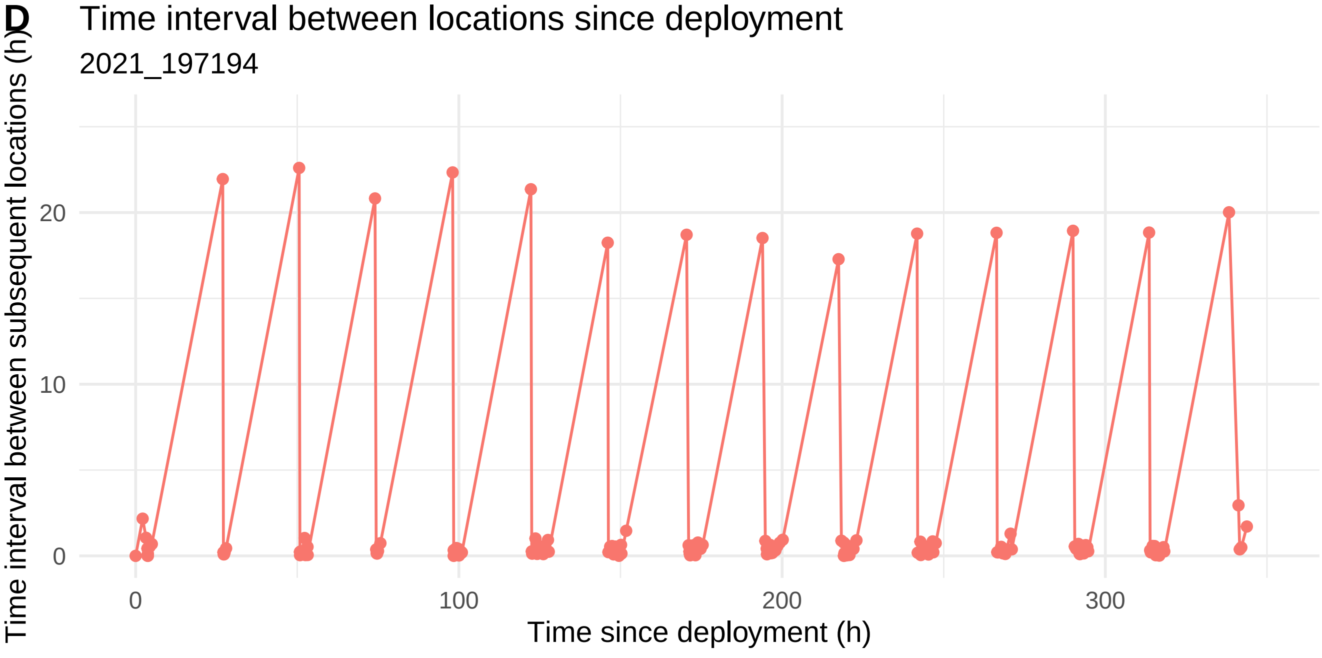
D
Time interval between locations since deployment
2021_197194
Time interval between subsequent locations (h)
0 10 20
0 100 200 300
Time since deployment (h)

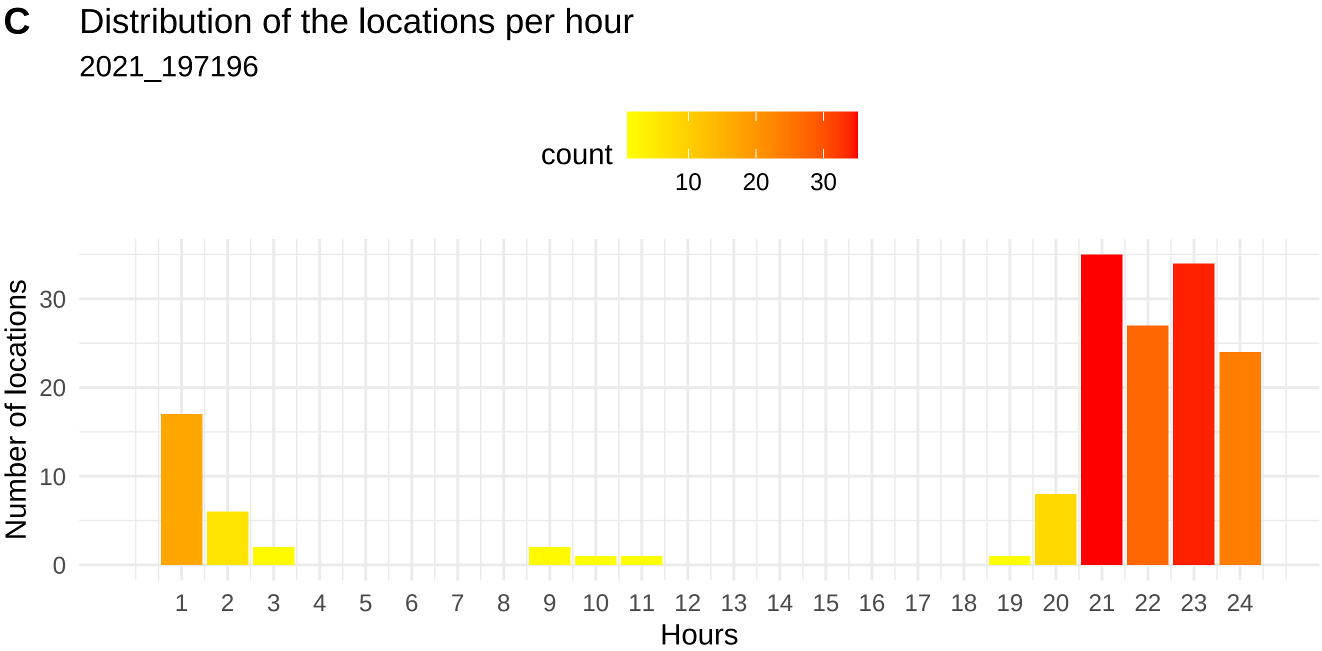
C
Distribution of the locations per hour
2021_197196
count
10
20
30
Number of locations
0
10
20
30
1
2
3
4
5
6
7
8
9
10
11
12
13
14
15
16
17
18
19
20
21
22
23
24
Hours

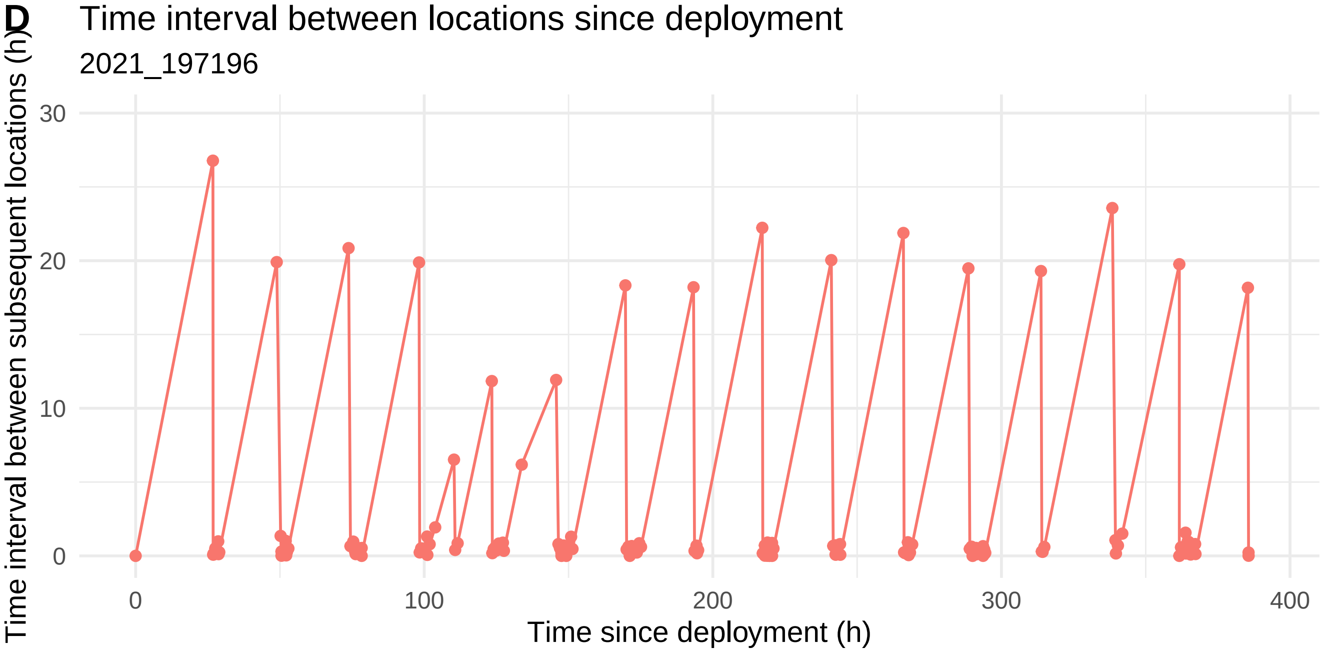
D
Time interval between locations since deployment
2021_197196
Time interval between subsequent locations (h)
0
10
20
30
0
100
200
300
400
Time since deployment (h)

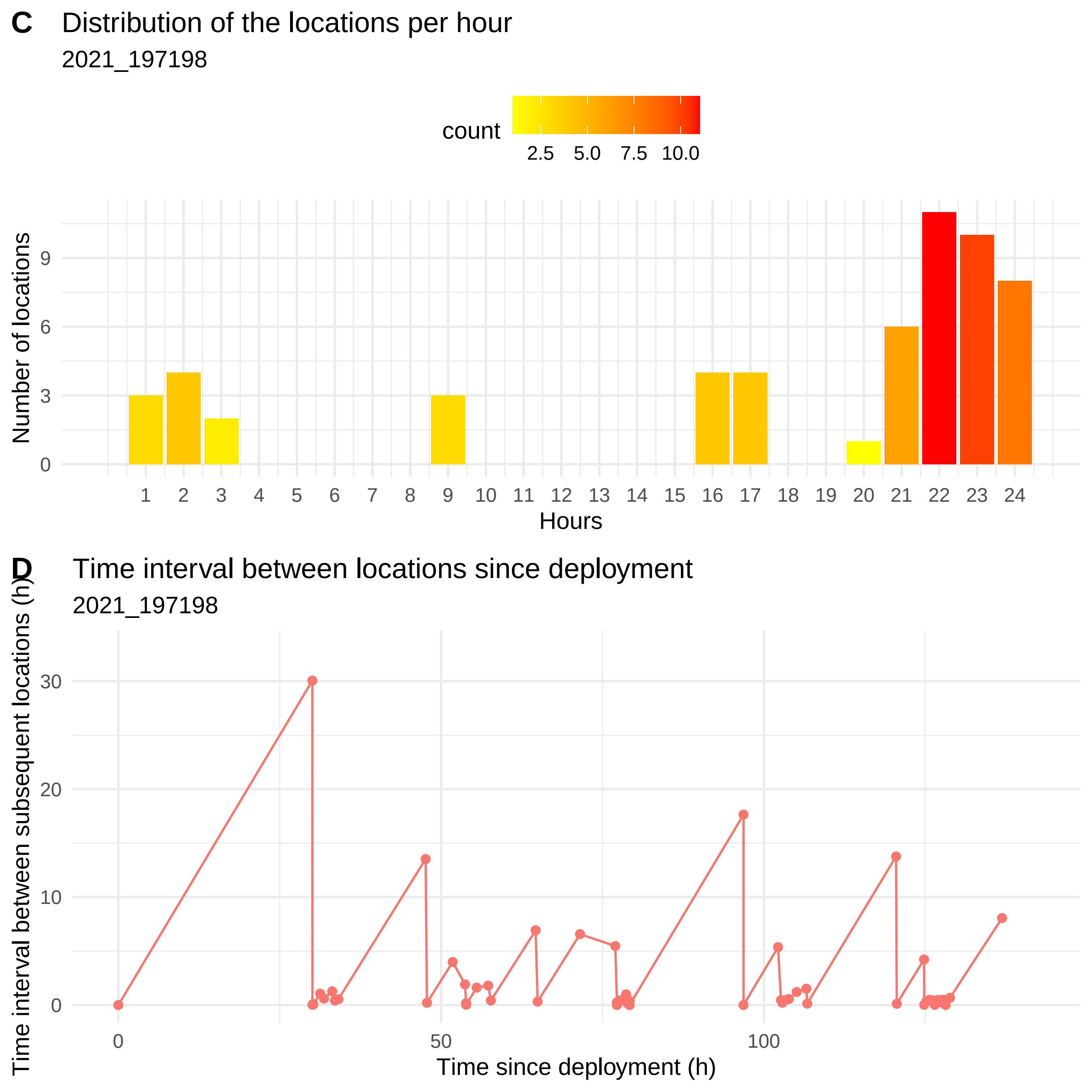
C
Distribution of the locations per hour
2021_197198
count
2.5
5.0
7.5
10.0
Number of locations
0
3
6
9
1
2
3
4
5
6
7
8
9
10
11
12
13
14
15
16
17
18
19
20
21
22
23
24
Hours
D
Time interval between locations since deployment
2021_197198
Time interval between subsequent locations (h)
0
10
20
30
0
50
100
Time since deployment (h)

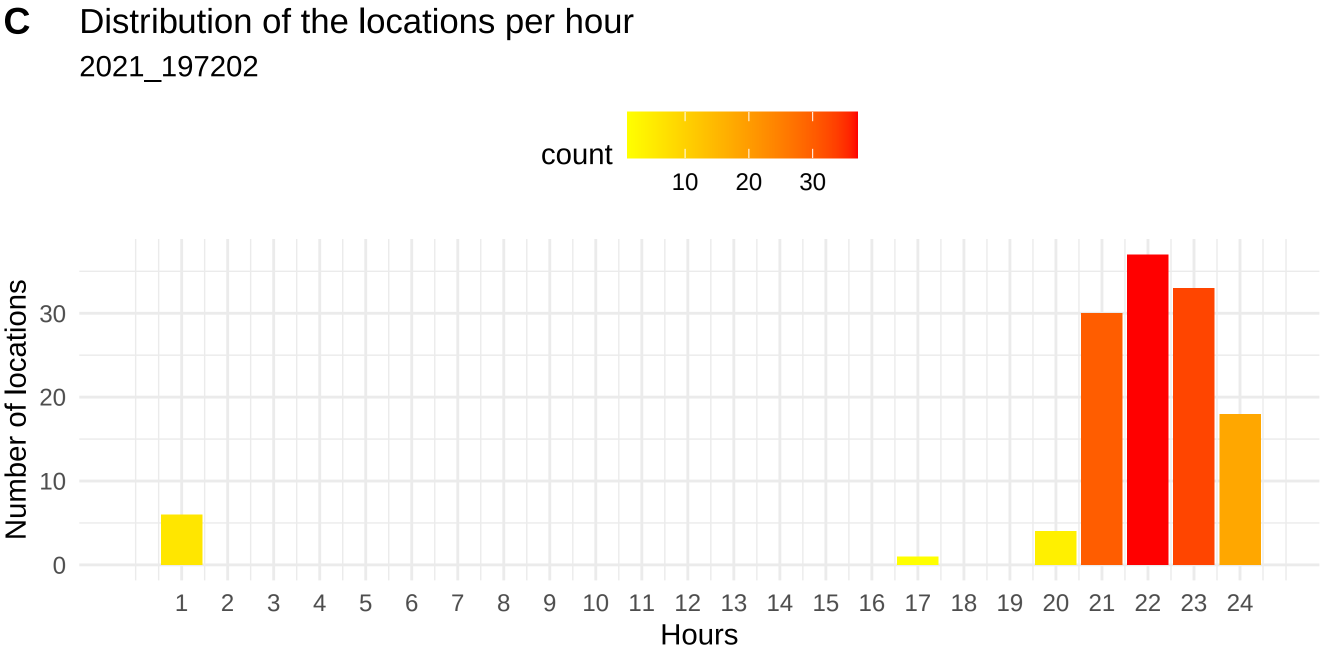
C
Distribution of the locations per hour
2021_197202
count
10
20
30
Number of locations
0
10
20
30
1 2 3 4 5 6 7 8 9 10 11 12 13 14 15 16 17 18 19 20 21 22 23 24
Hours

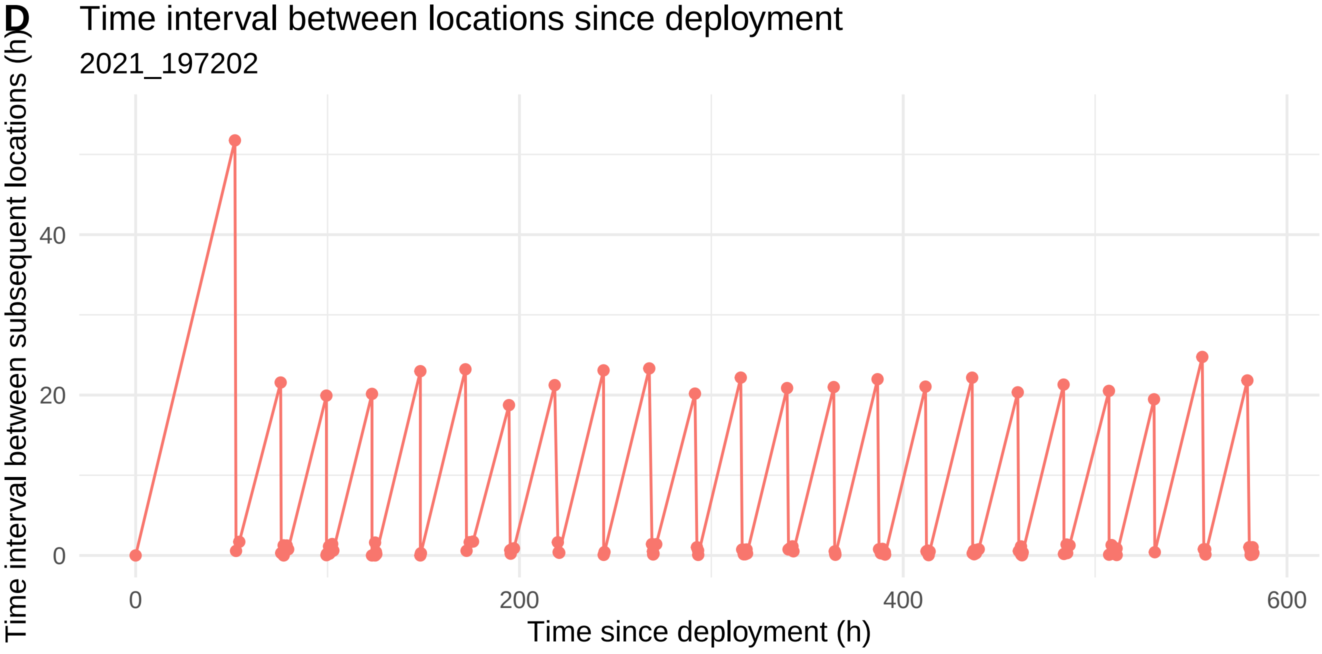
D
Time interval between locations since deployment
2021_197202
Time interval between subsequent locations (h)
0
20
40
0
200
400
600
Time since deployment (h)

Supplement 2:

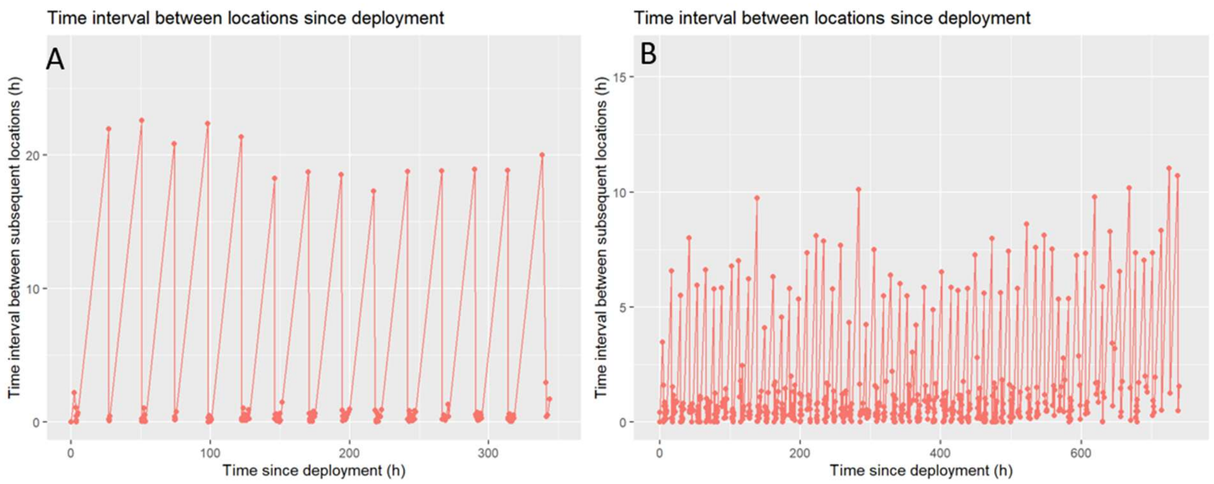

Figure I: The time difference between subsequent positions for (A) one SPOT (ARGOS only) and (B) one SPLASH tag (ARGOS and GPS) over the deployment period. In both cases, we received multiple positions per hour during the peak satellite coverage, with longer pauses between the two peaks. This pattern resulted in short mean and median periods (Table A below) between observations, which many studies used to determine their time step. In our case, this number seems misleading, suggesting a much smaller resolution than justified by the data. We inspected the timing between subsequent positions of all tags (Supplement 1 above). While 16 of the tags showed two clusters of ARGOS positions approximately 12 hours apart, the other 12 provided ARGOS positions within a few hours only once daily and in the middle of the night. The fast-lock GPS onboard the SPLASH tags performed poorly, with only 7 to 20% (average: 10%) of the successful GPS positions (i.e., 7 to 162 locations) transmitted to the ARGOS satellite over the entire deployment period (Table A). Taking it all together, we applied a 12-hour time step.

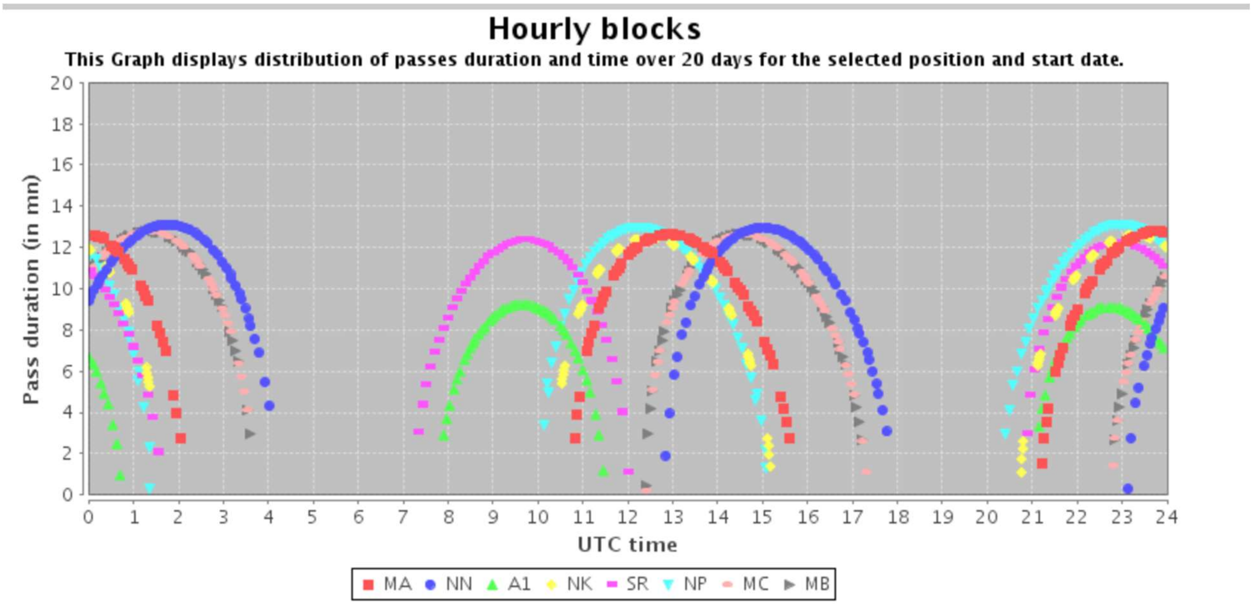

Figure II: Satellite Coverage of the 8 ARGOS satellites for the St Lawrence Estuary (~48N by 64W for November/December 2020 in UTC. The peaks and gaps move very little for the same location over the years but shift with longitudes over the time of the day. Higher latitudes (towards the poles) have better coverage, while lower latitudes (towards the equator) have longer gaps.

Table A: Summary of satellite tag deployments. PTT number (* SPLASH tag) with the date of deployment (Dep. Date) and last location (loc_date), number (NoD) of days with positions, the total number of ARGOS (loc) and GPS (bracket) locations received, number of daily locations (loc/d) for ARGOS and ARGOS/GPS combined (in brackets), and median time step (TS, in hours (decimal) between subsequent locations, followed by the number of ARGOS locations per class (we counted GPS locations as ARGOS location class 3 in the analysis), the total distance (Dis.) the animal travelled (straight line between subsequent locations), and the daily distance (km/d).

Tag	Dep. Date	Loc_date	NoD	LOC(GPS)	loc/d	TS	LC3	LC2	LC1	LC0	LCA	LCB	Dis.	km/d
106740	04-11-2014	05-01-2015	62	438	7.05	1.70	2	3	3	6	17	407	1779.9	28.71
141347	30-07-2015	08-08-2015	9	130	14.56	0.85	1	1	2	3	10	113	115.6	12.84
141345	09-08-2015	19-08-2015	10	121	12.00	0.78	2	2	6	6	11	94	137.8	13.78
141348	18-11-2015	04-01-2016	47	360	7.70	0.87	24	30	41	26	57	182	610.8	13.00
100388	20-10-2016	09-11-2016	20	82	4.05	1.37	1	0	6	6	7	62	631.2	31.56
100386	18-11-2016	13-01-2017	56	591	10.75	0.95	7	5	11	20	81	467	1419.6	25.35
158387	27-06-2017	13-07-2017	17	198	11.71	0.95	1	0	4	4	12	177	207.4	12.20
158386	29-06-2017	30-09-2017	94	1082	11.51	0.67	8	25	64	124	162	699	1161.9	12.36
100383	31-07-2017	14-08-2017	15	79	5.27	1.56	1	0	2	11	7	58	264.9	17.66
158386*	03-07-2021	30-07-2021	28	373(48)	13.32(15.0)	0.60	0	0	0	2	6	365	431.8	15.42
158389*	04-10-2021	04-11-2021	32	643(119)	20.09(20.8)	0.47	8	15	32	47	96	445	1407.3	43.98
173510	12-10-2020	25-10-2020	14	54	3.71	1.60	0	0	2	0	10	40	94.69	6.76
173512	06-11-2020	29-11-2020	24	35	1.46	14.81	1	1	1	4	9	19	76.1	3.17
173513	15-11-2020	13-12-2020	29	181	6.24	0.41	12	23	17	11	52	66	211.2	7.28
173514	28-11-2020	29-01-2020	63	219	3.48	0.68	5	12	28	34	50	90	4758.5	75.53
173515	05-10-2020	29-01-2020	115	380	3.30	1.13	8	23	50	79	104	116	3528.1	30.68
173516	02-10-2020	19-10-2020	17	47	2.76	0.50	3	1	5	14	2	22	176.15	10.36
173518	05-10-2020	14-01-2020	102	237	2.32	0.95	8	20	35	37	54	83	6993.7	68.57
197177*	04-10-2021	17-10-2021	14	134(28)	9.5(11.5)	0.66	0	2	3	3	6	120	732.1	52.29
197178*	21-06-2021	28-07-2021	38	572(162)	15.05(19.31)	0.53	1	1	5	13	36	516	933.5	24.57
197179*	21-06-2021	04-07-2021	12	161(5)	13.41(13.82)	0.58	1	2	1	6	12	139	548.6	45.72
197181*	29-09-2021	11-10-2021	13	104(7)	8.0(8.53)	0.63	0	0	1	6	9	88	187.9	14.45
197182*	29-09-2021	25-10-2021	27	452(127)	16.74(21.44)	0.43	5	17	37	32	63	298	1054.9	39.07
197194	21-10-2021	05-11-2021	15	186	12.40	0.33	5	8	14	14	36	109	1348.3	89.89
197195	28-11-2020	05-01-2020	39	245	6.28	0.43	26	37	56	29	37	60	4434.8	113.71
197196	21-10-2021	06-11-2021	16	157	9.81	0.43	2	3	6	11	27	108	782.3	48.89
197198	20-10-2021	26-11-2020	7	56	8.00	0.45	1	1	0	0	10	44	35.92	5.13
197202	17-11-2021	12-12-2021	26	129	4.96	0.60	1	10	5	8	28	77	2354.8	90.57

Table B: Number of predicted locations and behavioural states from the general model, including all data and results divided by season (upper table is identical to Table 2 in the main text). The lower table shows the model outputs for the separately analyzed summer, fall and winter data.

State	All data	Summer	Fall	Winter
ARS	1176	349	505	322
uncertain ARS	127	21	56	50
uncertain Transit	79	6	34	39
Transit	274	5	106	163
Proportion in ARS	71%	91.6%	72.0%	56.1%
Speed in km/h				
ARS mean (SD)	0.53 (0.68)	0.53(0.60)	0.51(073)	0.52(0.65)
Transit mean (SD)	5.74 (2.54)	4.87(1.57)	5.13(1.95)	6.05(2.85)
Max speed	12.48	10.7	11.42	12.48

State	Summer	Fall	Winter
ARS	348	542	346
uncertain ARS	19	81	92
uncertain Transit	7	38	48
Transit	7	138	191
Proportion ARS	91.3%	67.8%	51.1%
Speed in km/h			
ARS mean (SD)	0.55(0.64)	0.49(0.66)	0.61(0.74)
Transit mean (SD)	4.58(3.36)	5.54(2.64)	6.32(2.65)
Max speed	9.18	12.15	15.63

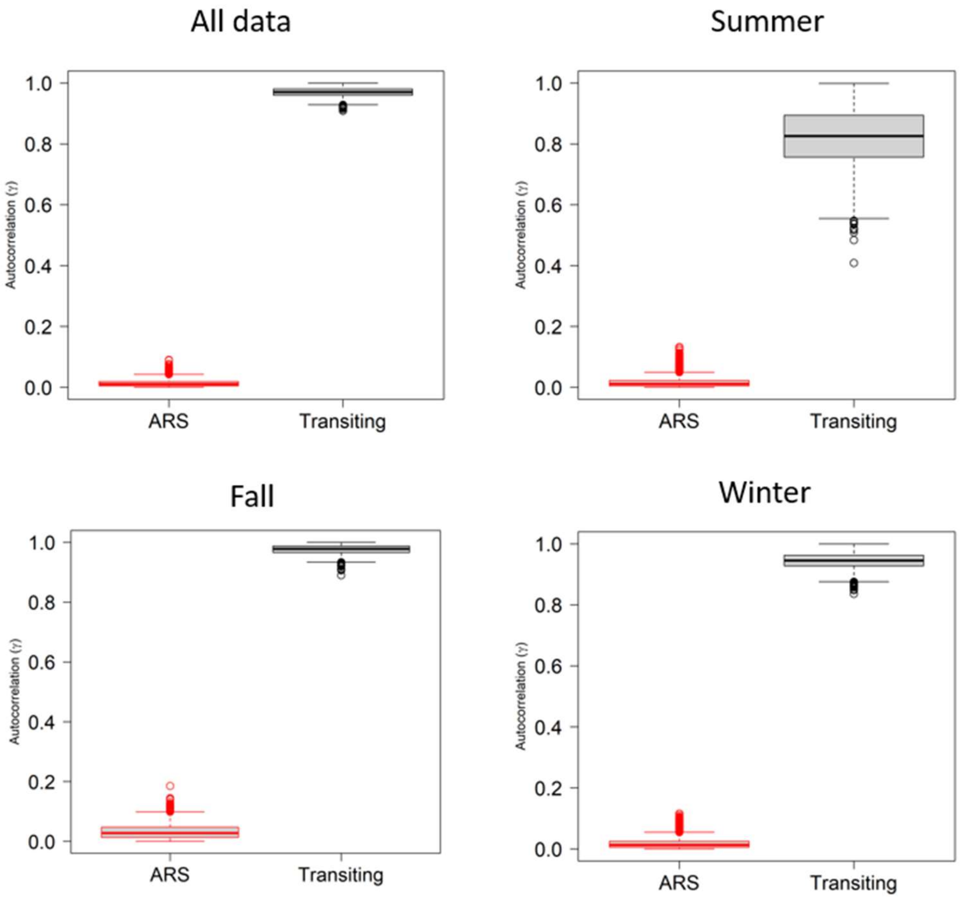

Figure III: Autocorrelation (gamma) for the hSSSM using all tag data and for summer, fall and winter data analyzed separately.

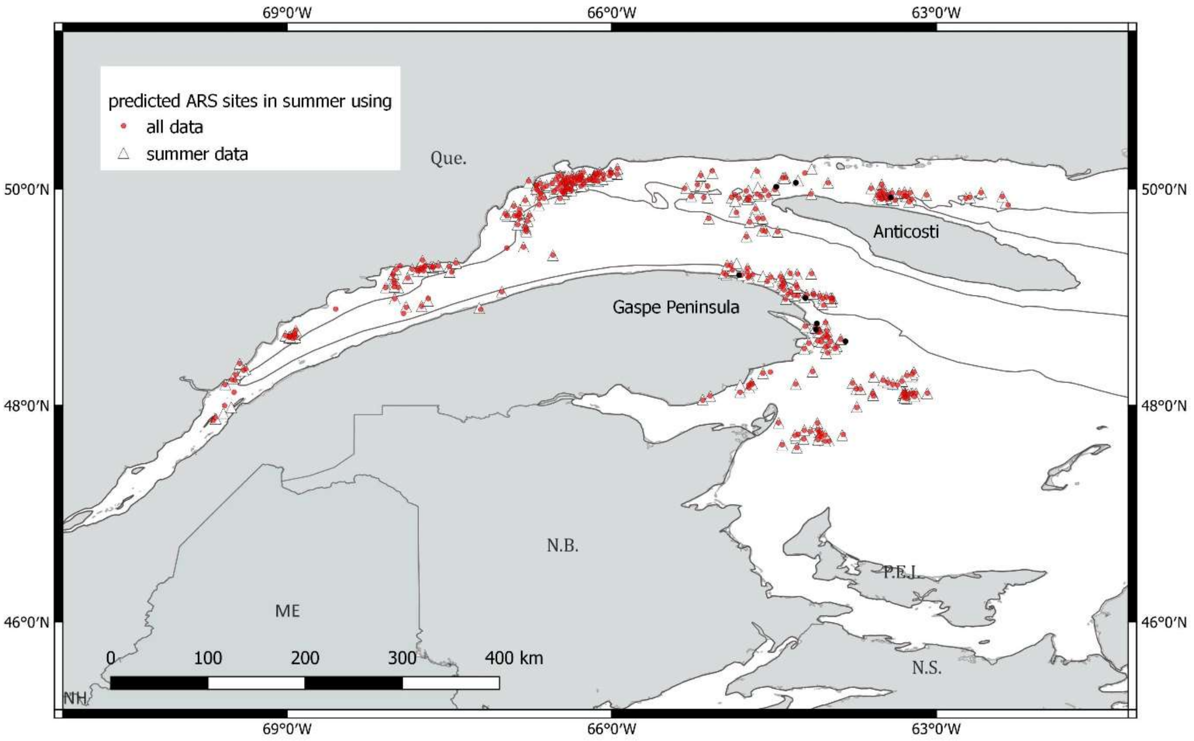

Figure IV: ARS states in the GSL in Summer (June to August) as predicted by the model using all data (red dots) and summer data only (white triangles). Black dots show deployment points.

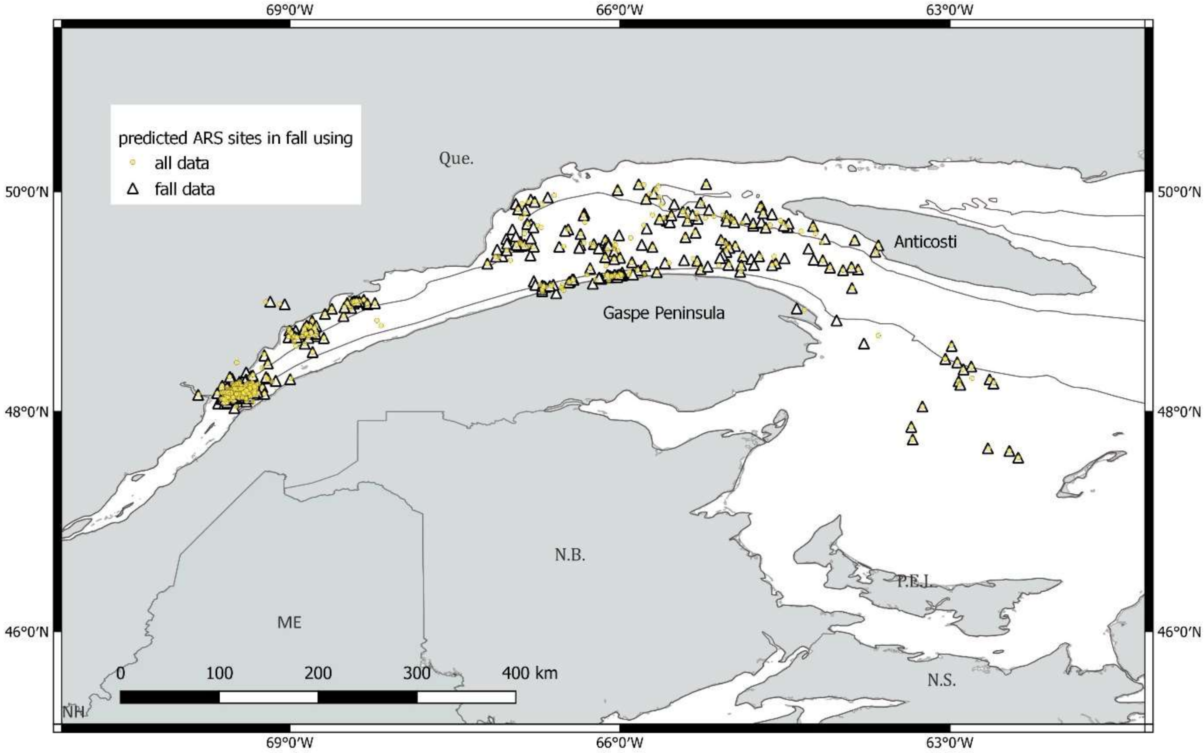

Figure V: ARS states in the GSL in Fall (September to November) as predicted by the model using all data (yellow dots) and fall data only (white triangles).

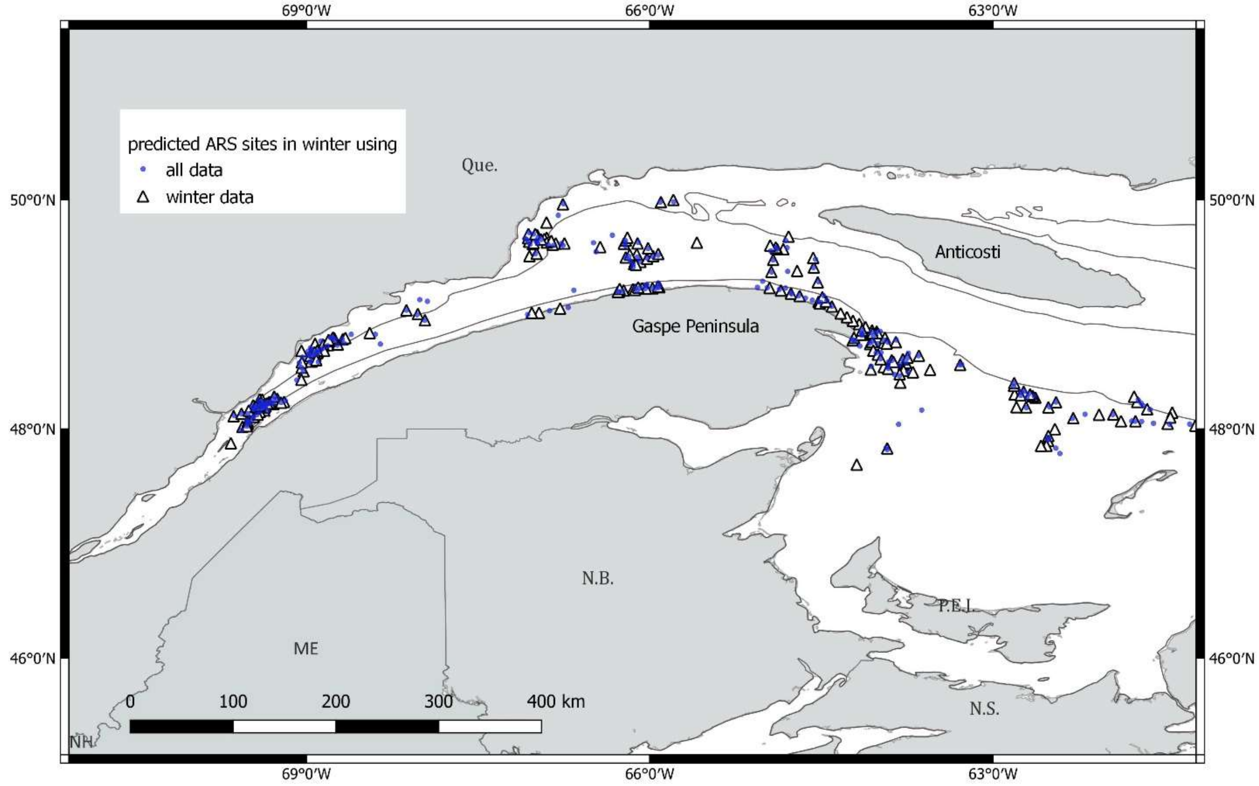

Figure VI: ARS states in the GSL in winter (December and January) as predicted by the model using all data (blue dots) and winter data only (white triangles).

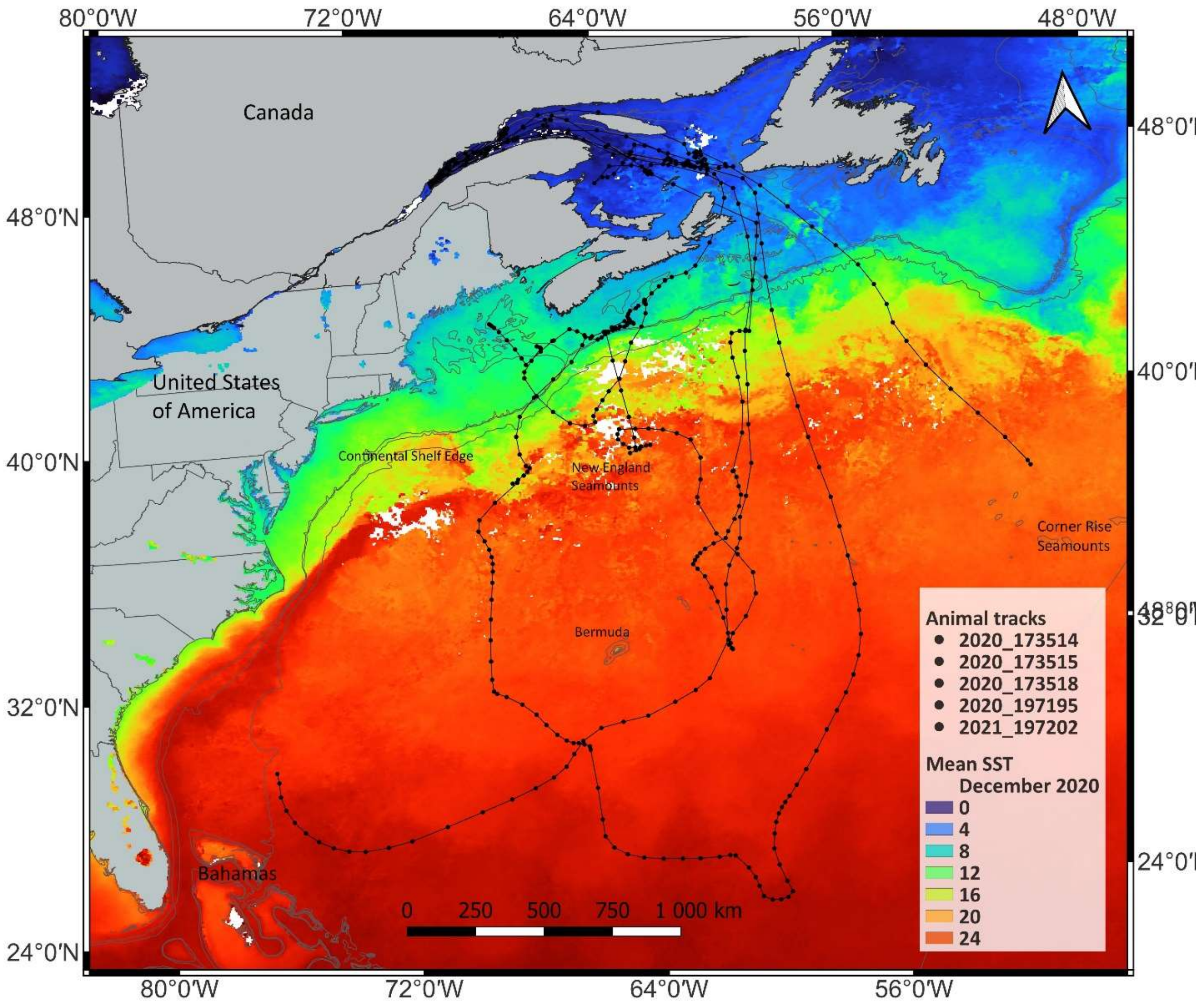

Figure VII: Animal track of five fin whales leaving the Shelf in December 2020 overlayed with monthly averages sea surface temperature (SST) for December 2020 (Aqua-MODIS Level-3 sea surface temperature (11µ daytime) 4km resolution, https://oceancolor.gsfc.nasa.gov/l3/)

Please note that the track of 2021_197202 ending north of Corner Rise Seamounts occurred in 2021, but the SST average in the figure is for December 2020. For simplicity, we plotted everything on one map. We plotted the track 2021_197202 with the SST for December 2021, and the temperature at the end of the track was 22 degrees Celsius.

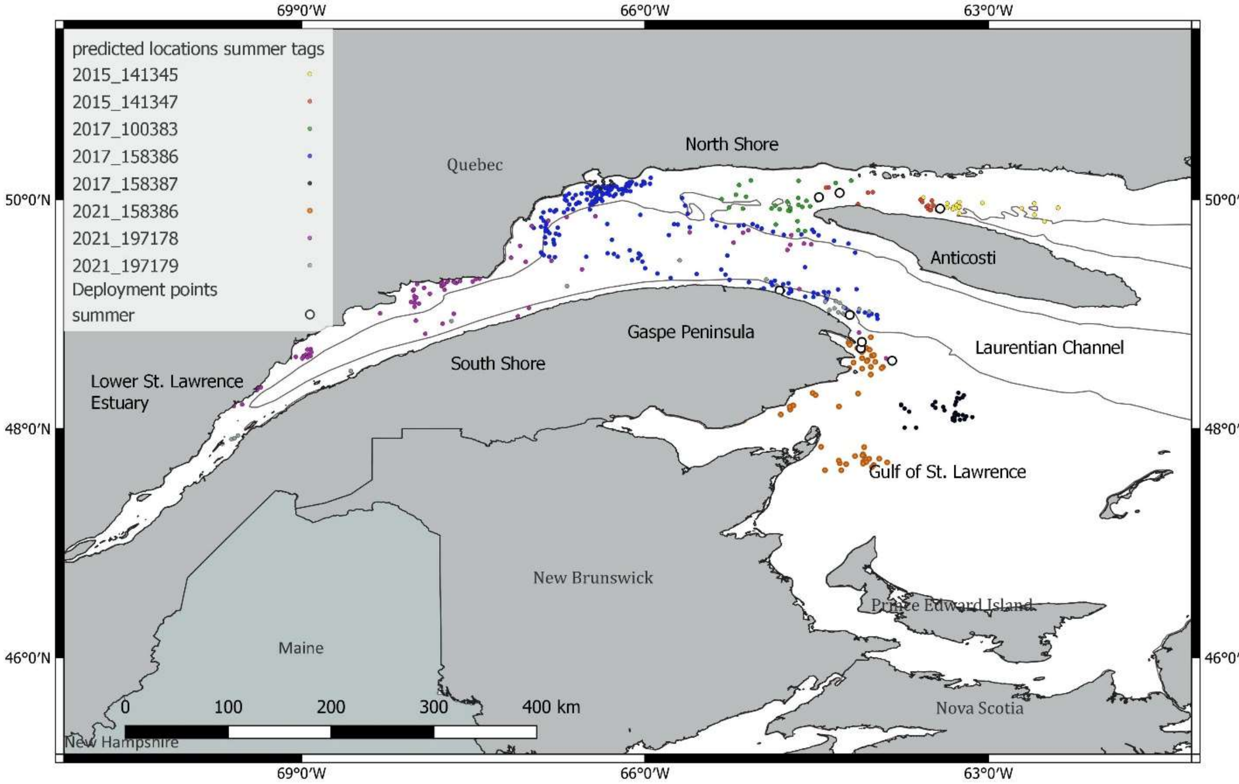

Figure VIII: Predicted locations of the tags deployed in summer with deployment point (white circles)

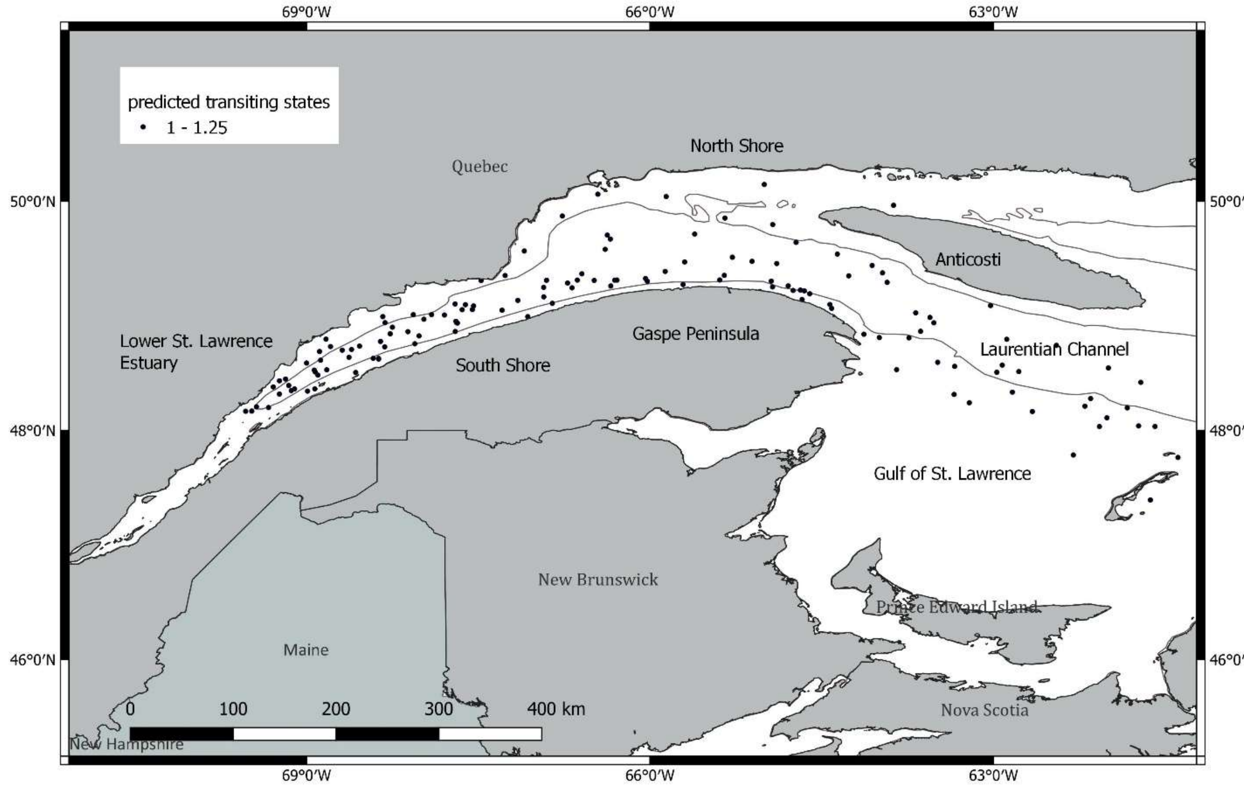

Figure IX: Transiting (b-mode 1-1.25) of all fin whales within the Gulf of St. Lawrence.

Supplement 3. Diagnostics Main Model

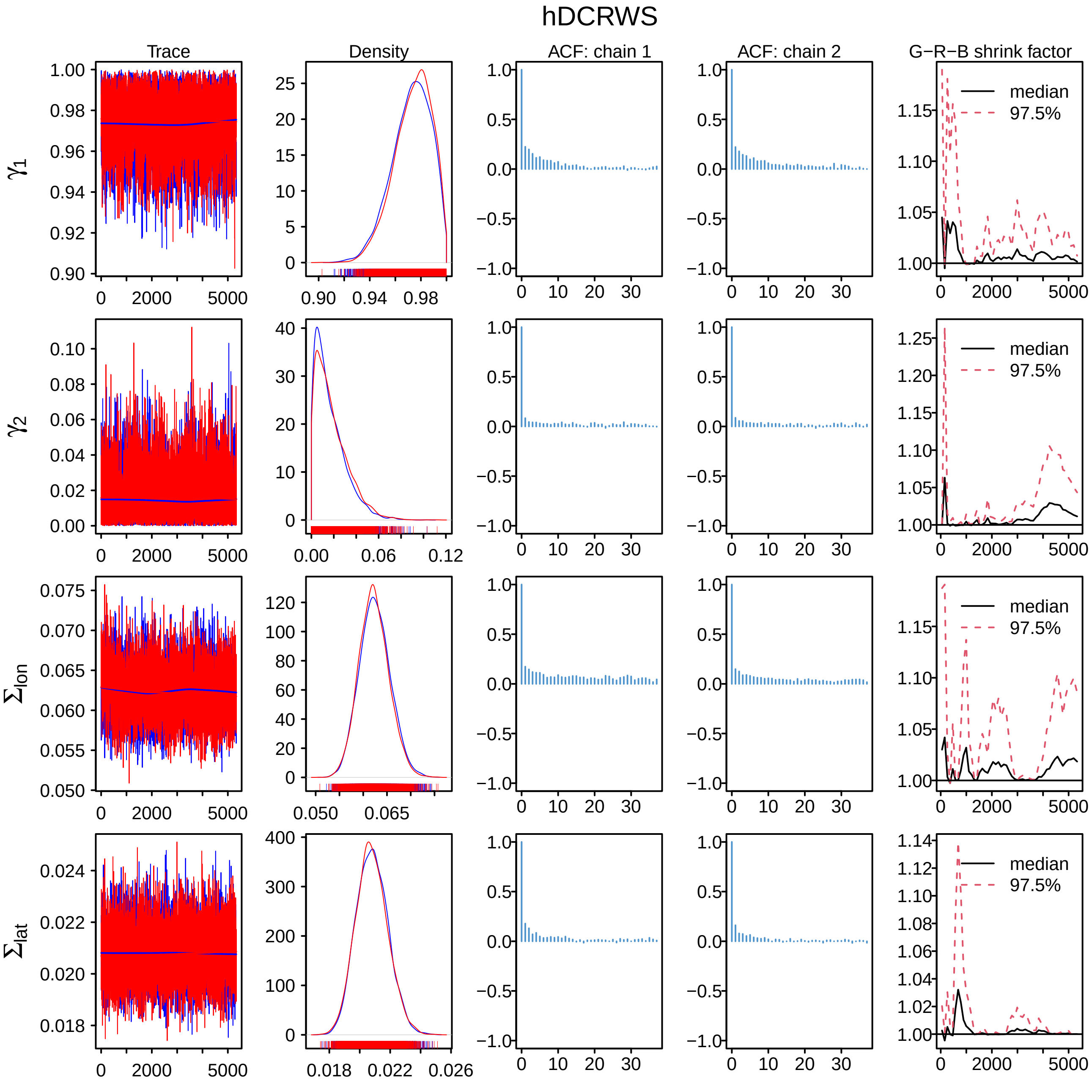
hDCRWS
Trace
Density
ACF: chain 1
ACF: chain 2
G−R−B shrink factor
median
97.5%
γ_1
γ_2
Σ_{lon}
Σ_{lat}

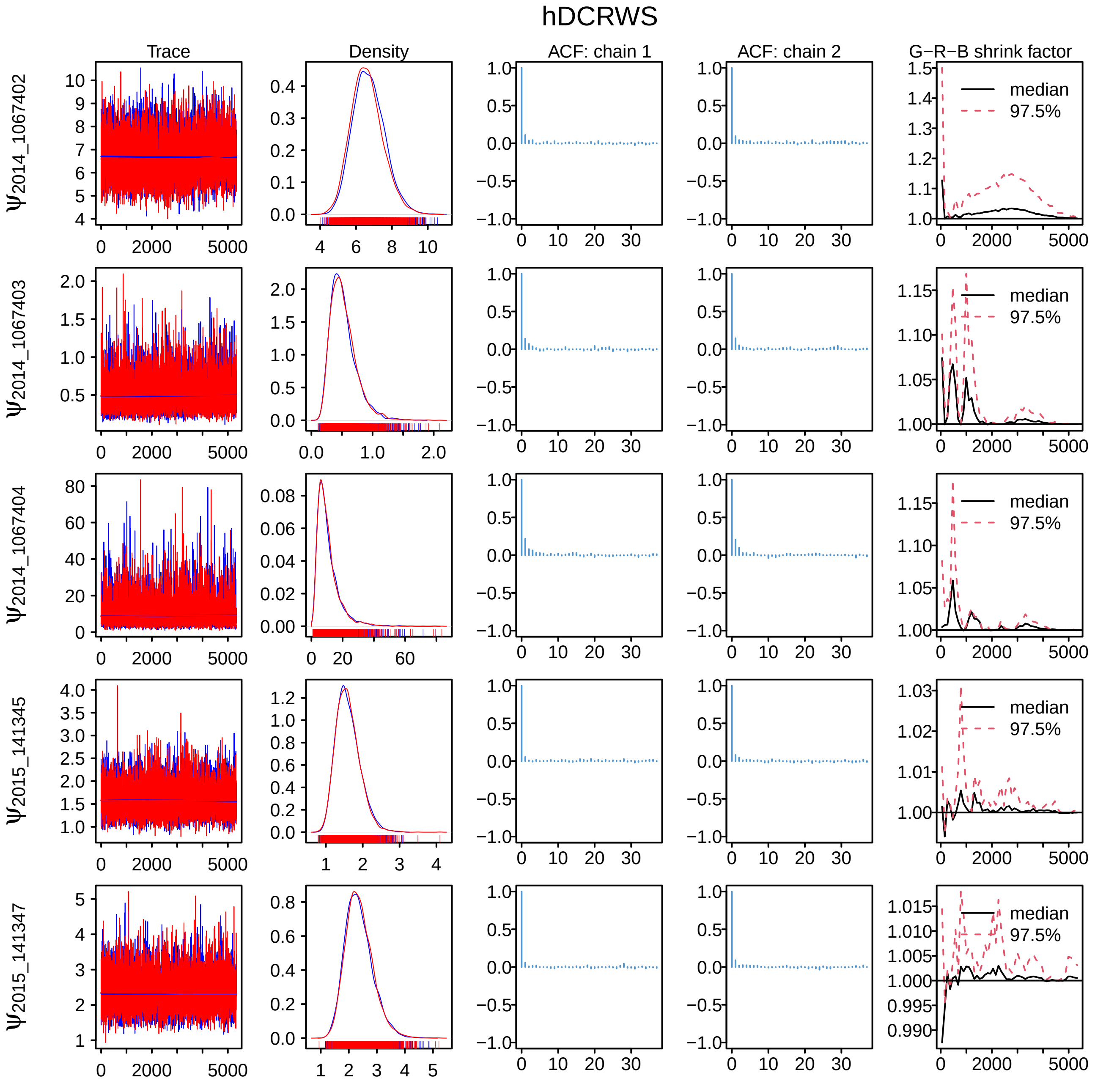
hDCRWS
Trace
Density
ACF: chain 1
ACF: chain 2
G−R−B shrink factor
median
97.5%
$\psi_{2014\_1067402}$
$\psi_{2014\_1067403}$
$\psi_{2014\_1067404}$
ψ_{2015_141345}
ψ_{2015_141347}

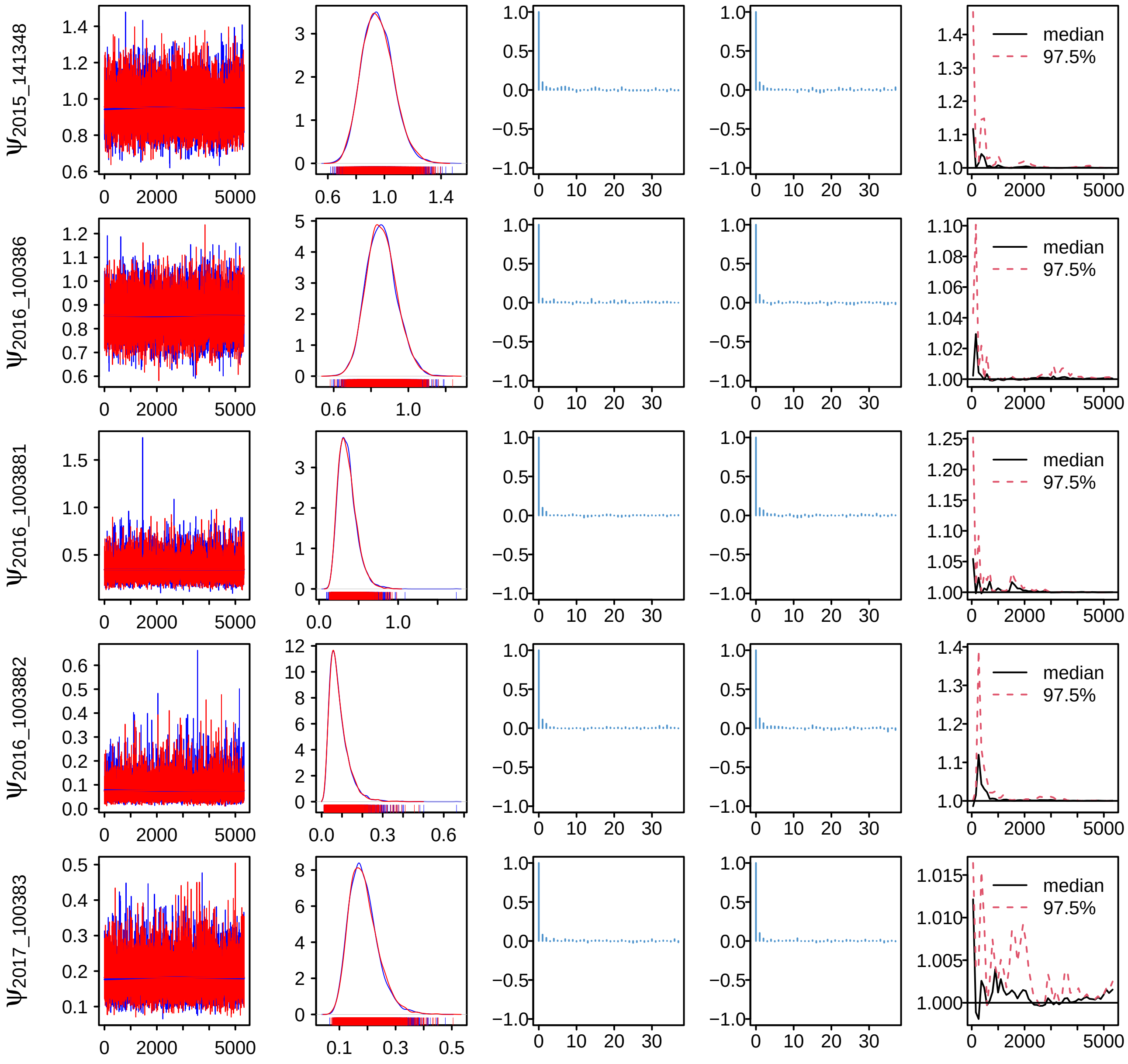

ψ_{2015_141348}
ψ_{2016_100386}
$\psi_{2016\_1003881}$
$\psi_{2016\_1003882}$
ψ_{2017_100383}
median
97.5%

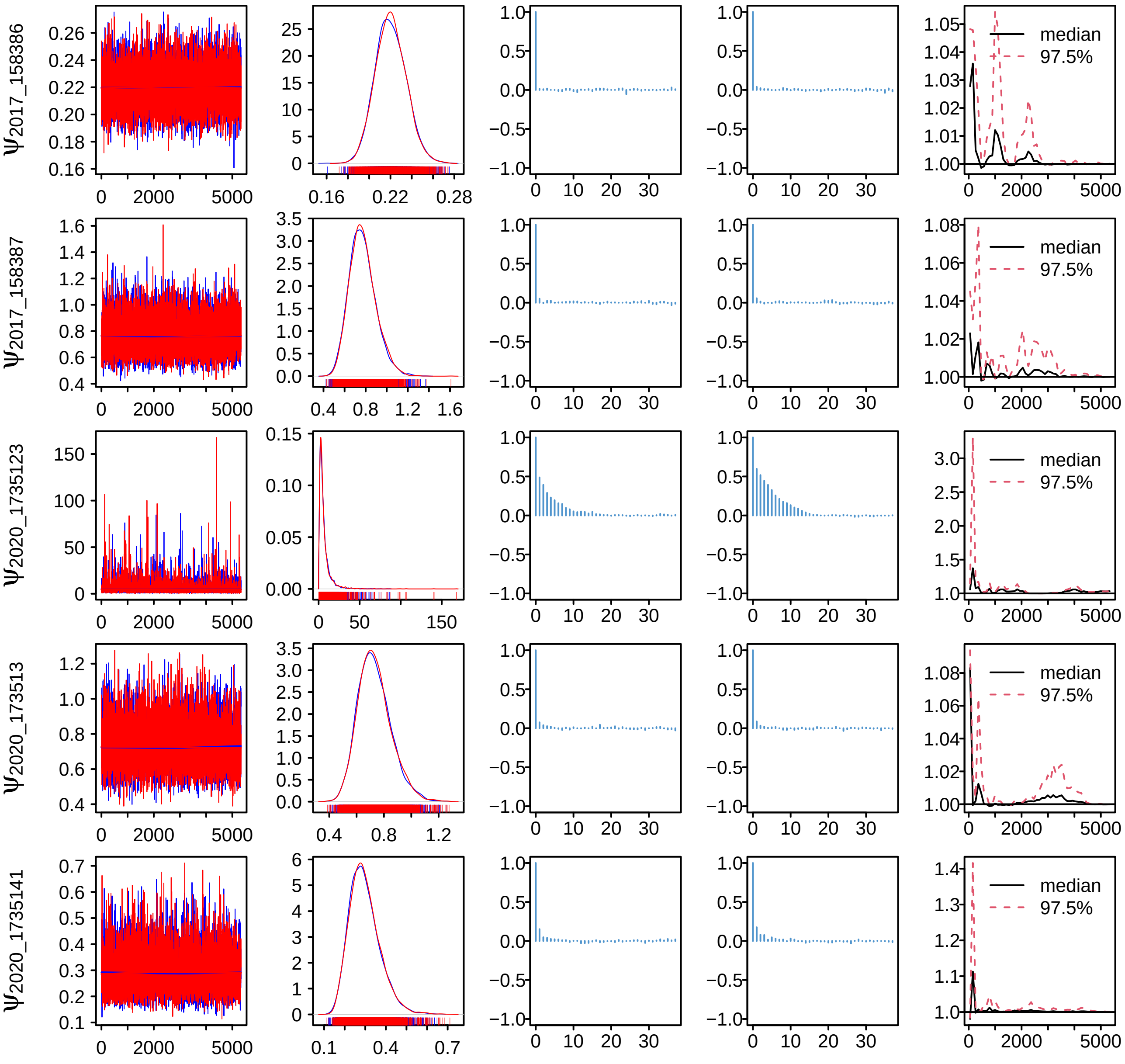

ψ_{2017_158386}
ψ_{2017_158387}
$\psi_{2020\_1735123}$
ψ_{2020_173513}
$\psi_{2020\_1735141}$
median
97.5%

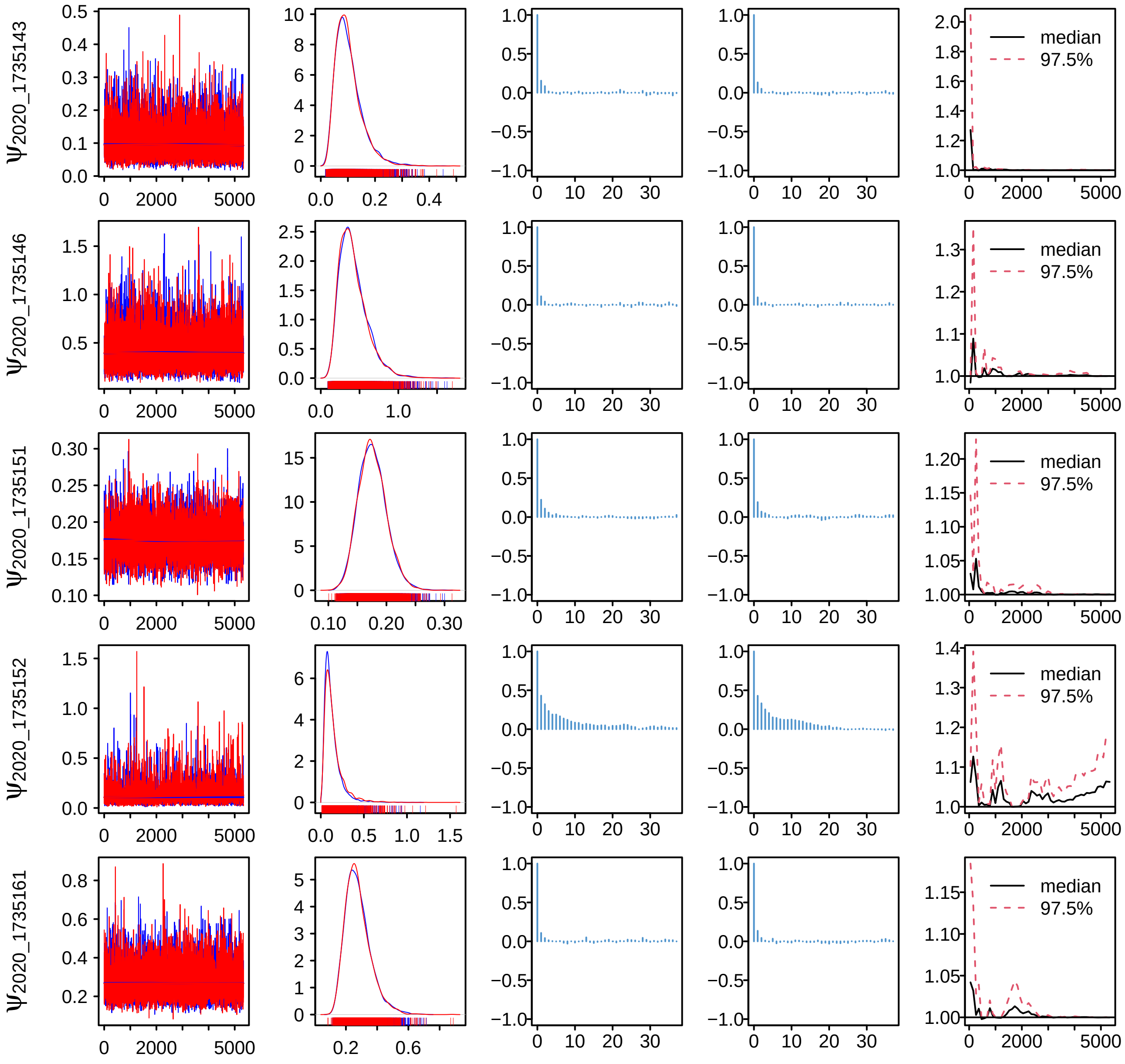

ψ2020_1735143
ψ2020_1735146
ψ2020_1735151
ψ2020_1735152
ψ2020_1735161
median
97.5%

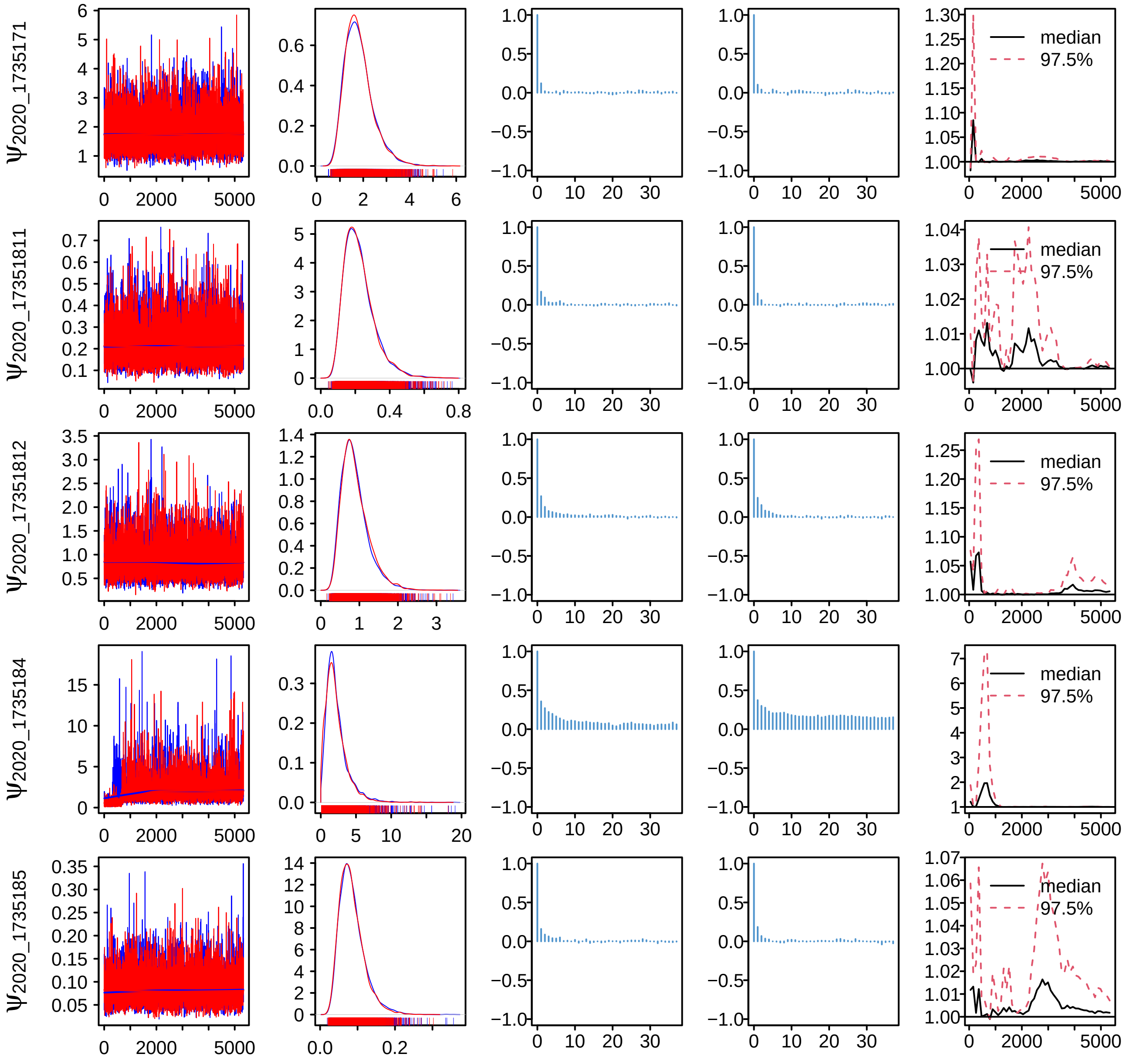

$\psi_{2020\_1735171}$
$\psi_{2020\_17351811}$
$\psi_{2020\_17351812}$
$\psi_{2020\_1735184}$
$\psi_{2020\_1735185}$
median
97.5%

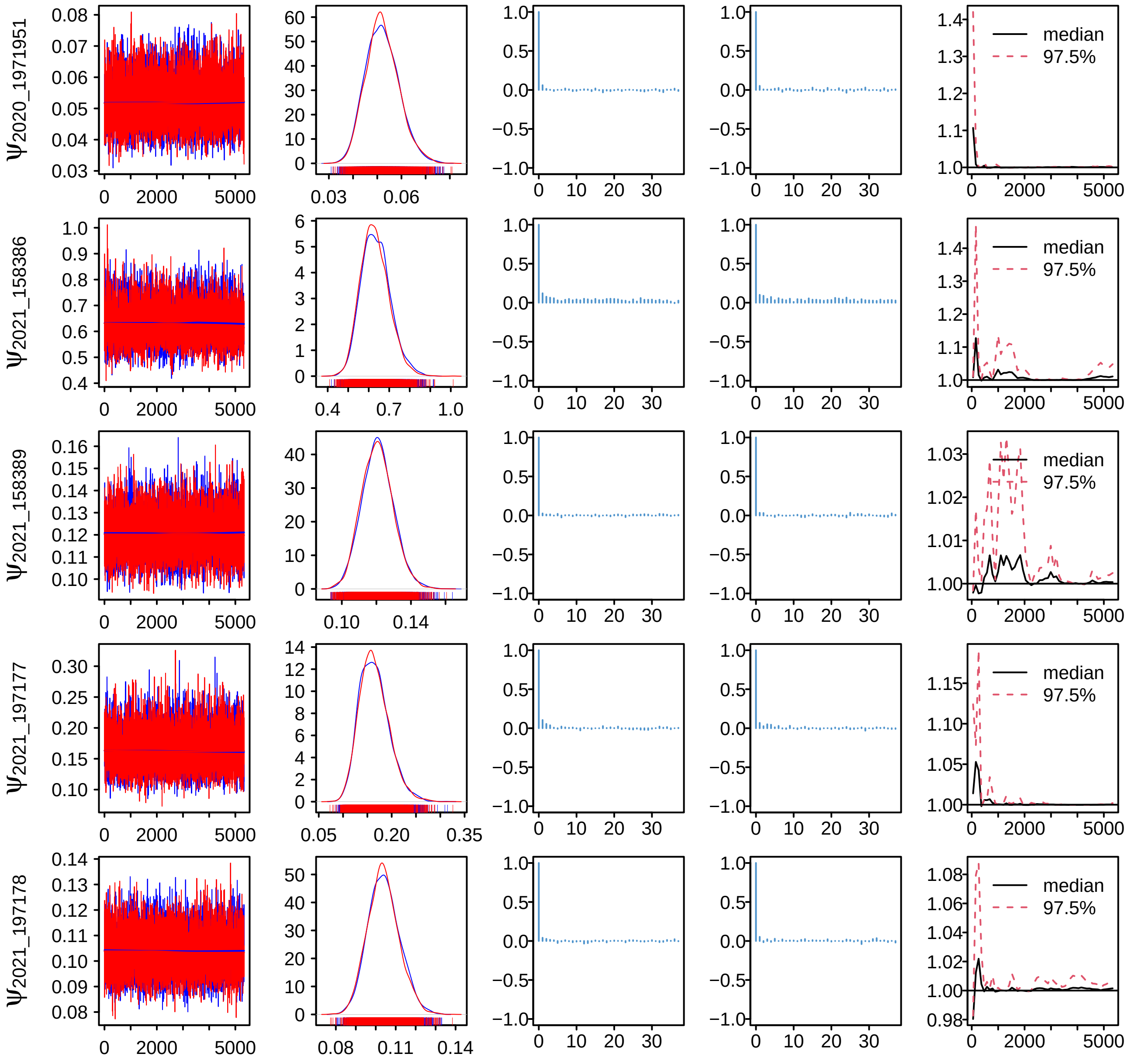

ψ2020_1971951
ψ2021_158386
ψ2021_158389
ψ2021_197177
ψ2021_197178
median
97.5%

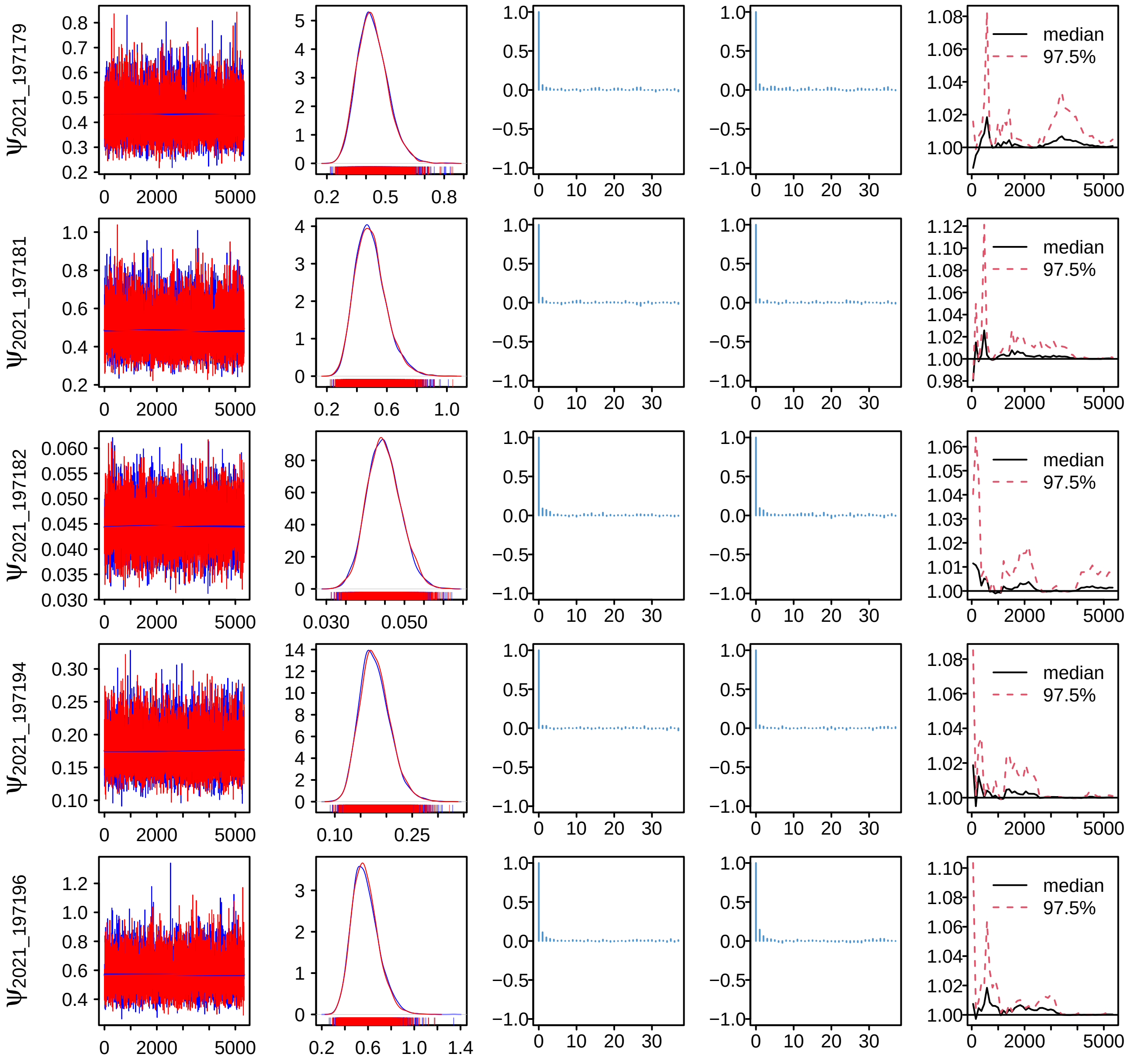

ψ_{2021_197179}
ψ_{2021_197181}
ψ_{2021_197182}
ψ_{2021_197194}
ψ_{2021_197196}
median
97.5%

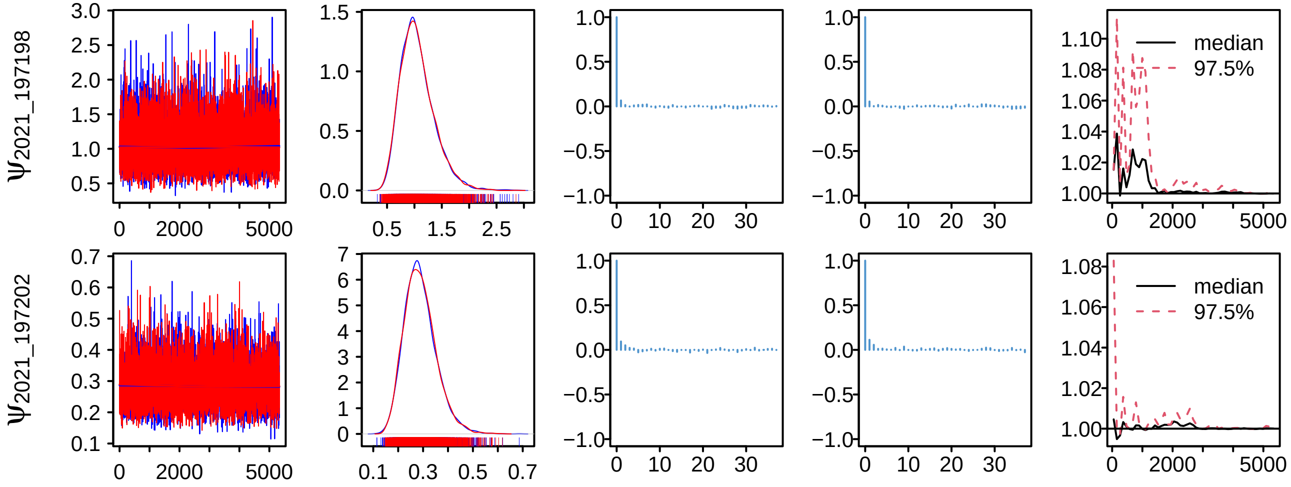

ψ_{2021_197198}
ψ_{2021_197202}
median
97.5%
median
97.5%
